# Design, Synthesis and Biological Evaluation of Aromatase Inhibitors Based on Sulfonates and Sulfonamides of Resveratrol

**DOI:** 10.3390/ph14100984

**Published:** 2021-09-27

**Authors:** Marialuigia Fantacuzzi, Marialucia Gallorini, Nicola Gambacorta, Alessandra Ammazzalorso, Zeineb Aturki, Marwa Balaha, Simone Carradori, Letizia Giampietro, Cristina Maccallini, Amelia Cataldi, Orazio Nicolotti, Rosa Amoroso, Barbara De Filippis

**Affiliations:** 1 Unit of Medicinal Chemistry, Department of Pharmacy, “G. d’Annunzio” University, 66100 Chieti, Italy; alessandra.ammazzalorso@unich.it (A.A.); simone.carradori@unich.it (S.C.); letizia.giampietro@unich.it (L.G.); cristina.maccallini@unich.it (C.M.); rosa.amoroso@unich.it (R.A.); 2 Unit of Anatomy, Department of Pharmacy, “G. d’Annunzio” University, 66100 Chieti, Italy; marialucia.gallorini@unich.it (M.G.); marwa.balaha@pharm.tanta.edu.eg (M.B.); amelia.cataldi@unich.it (A.C.); 3 Unit of Medicinal Chemistry, Department of Pharmacy, “A. Moro” University, 70121 Bari, Italy; nicola.gambacorta1@uniba.it (N.G.); orazio.nicolotti@uniba.it (O.N.); 4 Institute for Biological Systems (ISB), Italian National Research Council, Monterotondo, 00015 Rome, Italy; zeineb.aturki@cnr.it; 5 Department of Pharmaceutical Chemistry, Faculty of Pharmacy, Kafrelsheikh University, Kafr El Sheikh 33516, Egypt

**Keywords:** aromatase inhibitors, breast cancer, cytochromes P450, docking, resveratrol, stilbene, sulfonates, sulfonamides

## Abstract

A library of sulfonate and sulfonamide derivatives of Resveratrol was synthesized and tested for its aromatase inhibitory potential. Interestingly, sulfonate derivatives were found to be more active than sulfonamide bioisosteres with IC_50_ values in the low micromolar range. The sulfonate analogues **1b**–**c** and **1j** exhibited good in vitro antiproliferative activity on the MCF7 cell line, evidenced by MTT and LDH release assays. Structure–activity relationships suggested that electronic and lipophilic properties could have a different role in promoting the biological response for sulfonates and sulfonamides, respectively. Docking studies disclosed the main interactions at a molecular level of detail behind the observed inhibition of the more active compounds whose chemical stability has been evaluated with nano-liquid chromatography. Finally, **1b**–**c** and **1j** were highlighted as sulfonates to be further developed as novel and original aromatase inhibitors.

## 1. Introduction

Breast cancer is one of the leading causes of cancer-related deaths in women. It is treated principally by surgical methods, radiotherapy, and endocrine therapies [1]. Many tumors occur through estrogen-dependent mechanisms, and about 70% of breast cancer patients are estrogen receptor α (ERα) positive (ER+). Aromatase, a member of the cytochrome P450 family (CYP19), is responsible for a key step of the biosynthesis of estrogens. CYP19 converts C19 androgens (androstenedione and testosterone) to aromatic C18 estrogens (estradiol and estrone) via three consecutive hydroxylation reaction steps. It has been broadly reported that high levels of estrogens stimulate the hormone-dependent breast cancer (HDBC) and metastasis in both pre- and post-menopausal woman [2]. A reduction in estrogen levels by aromatase inhibition is crucial for the management of hormone-sensitive breast cancer. Under some situations (e.g., post-menopause), aromatase has a key role in estrogen production, and inhibitors have been shown to function as chemo-preventive agents in HDBC. In fact, estrogen deprivation is an effective therapeutic intervention that has been clinically established by the inhibition of the aromatase enzyme.

**Figure 1:**
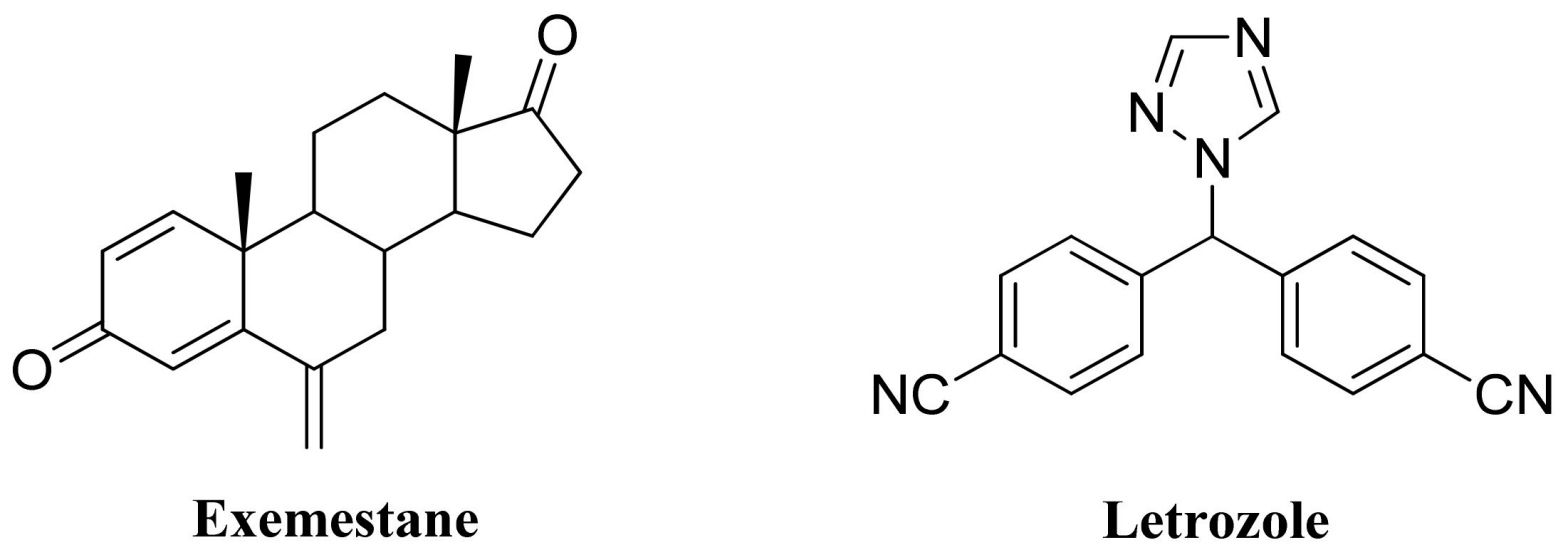
Chemical structures of the most representative steroidal and non-steroidal AIs.

Several aromatase inhibitors (AIs) have been developed including steroidal and non-steroidal compounds [3]. The first type of agents are androstenedione analogues, such as Exemestane (Figure 1), which binds to the active site of the aromatase via covalent interactions with the heme iron in the catalytic site. Non-steroidal AIs, such as Letrozole (Figure 1), interact reversibly with the active site of the aromatase through non-covalent interaction [4]. Non-steroidal derivatives generally own planar aromatic structures joined to an azole (imidazole or triazole) ring system (Figure 1) [5] and the heterocyclic nitrogen interacts with the heme iron of the aromatase enzyme [6].

Although their effectiveness is superior to that of Tamoxifen, which is the most used drug in endocrine therapy in ER+ breast cancer, the search for novel classes of AIs is still required because of their side effects, such as bone loss and cardiovascular disease, and of the potential resistance occurrence due to prolonged use. The discovery of potent non-steroidal aromatase inhibitors (NSAIs) provided with fewer side effects and cell resistance is thus extremely pursued [7,8]. The triazole ring system replacement and the proper functionalization of additional aromatic/cyclic moieties represent suitable strategies for addressing a more selective inhibition towards the aromatase enzyme. Recently, several structurally different NSAIs have been reported and a comprehensive pharmacophore was provided [8].

**Figure 2:**
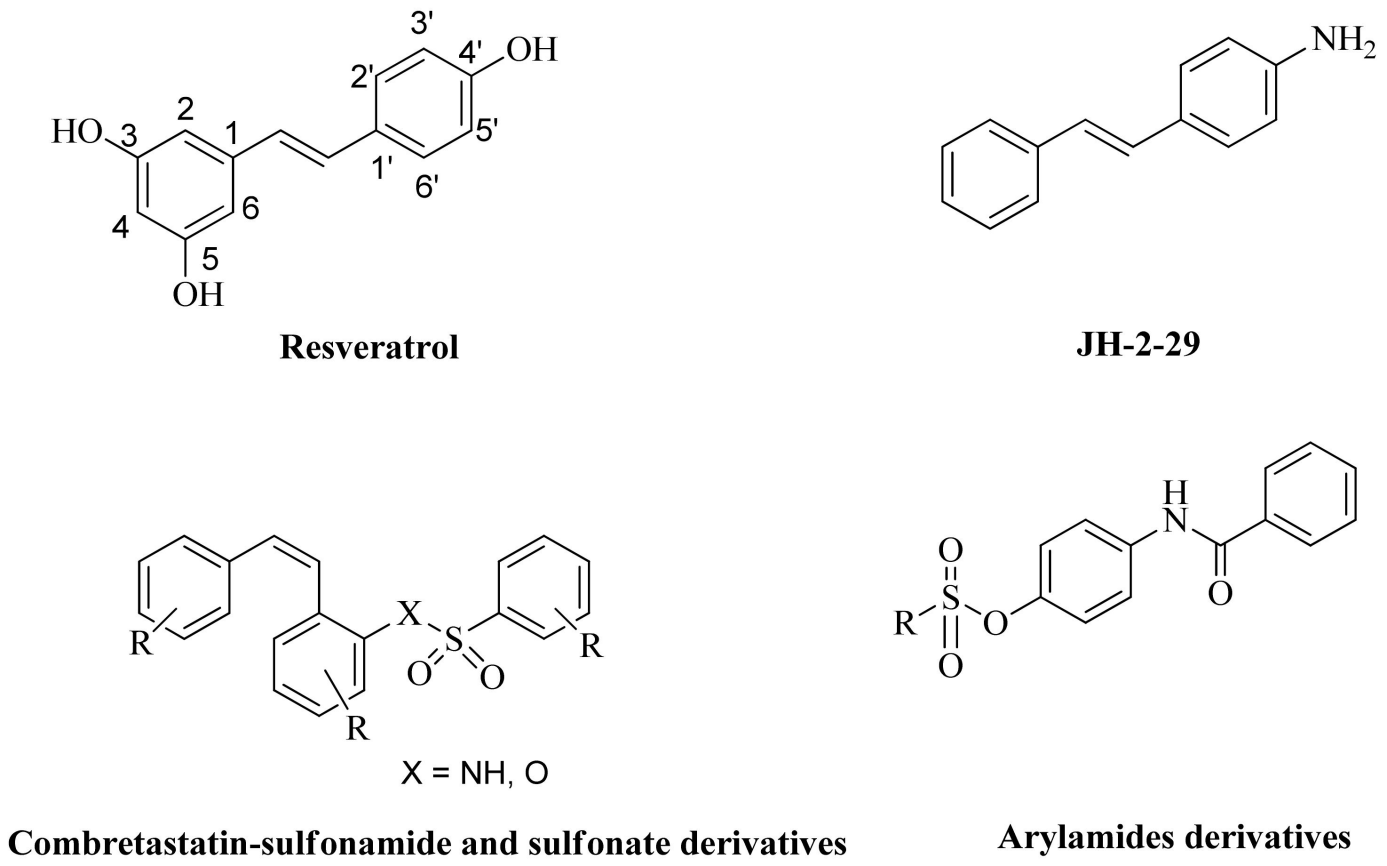
Structures of RSV, JH-2-29, and some sulfonate and sulfonamide RSV derivatives.

Among them, natural products represent lead structures for new drug discovery and continue to inspire the design of new potential drugs [9,10]. Resveratrol (trans-3,4′,5-trihydroxystilbene, RSV, Figure 2) is a polyphenolic phytoalexin found in grapes, peanuts, and mulberry. It has attracted the attention of biomedical researchers because of a plethora of beneficial physiological effects [11]. About 20 years ago, RSV was also identified as a potent cancer chemo-preventive agent acting on the three major stages of carcinogenesis (i.e., tumor initiation, promotion, and progression) [12]. Investigations in this field evidenced multiple intracellular targets of RSV affecting cell growth, inflammation, apoptosis, angiogenesis, tumor invasion, and metastasis [13,14]. Although RSV can modulate multiple stages of carcinogenesis [12], along with the relatively simple structure and low toxicity, it is not a suitable drug candidate due to its questionable pharmacokinetic profile [15]. It is rapidly and extensively metabolized and excreted; thus, the biological effects elicited by RSV may be largely attributed to its metabolites [16]. The presence of free hydroxyl groups that are highly conjugated in vivo causes a low tissue concentration [17]. For this reason, there is continuing interest in the design and synthesis of RSV derivatives with different substitution patterns with the aim to enhance potency, selectivity, and in vivo stability [18,19,20,21,22].

Aromatase activity is slightly affected by RSV, showing an IC_50_ of 80 μM [12,23]. In this respect, a series of RSV analogues displayed much greater inhibition [21,24]. A research work demonstrated that the introduction of the para-amino group into the trans-stilbene core of RSV proved essential for aromatase inhibitory activity. JH-2-29 (Figure 2) showed an IC_50_ of 22 μM, and similar results were obtained for its derivatives [11,25].

The sulfonamide core (Figure 2) represents a key motif in medicinal chemistry [26] and sulfonamide-containing compounds are extensively used drugs for the treatment of different illnesses, including cancers [27], diabetes [28], psychosis [29], and other central nervous system (CNS) disorders [30]. They show anti-inflammatory, antimicrobial [31], anticonvulsant, and antidepressant activities, as well as carbonic anhydrase inhibition [32]. Furthermore, the sulfonamide group generally imparts stability and crystallinity, and sulfonamide derivatives are easy to prepare and show a desirable pharmacokinetic profile as far as oral absorption and low side-effects are concerned. Notably, sulfonamide-based compounds have been described as potent aromatase inhibitors [33,34,35,36,37]. Molecular docking studies, conducted on different sulfonamide derivatives, highlighted that the sulfonamide group is involved in H-bonding with the active site of aromatase [38]. Surprisingly, few studies have instead addressed the sulfonate moiety, despite its importance for bioisosteric replacement in the rational design of new drugs [39]. Arylamide derivatives with sulfonate and sulfamate moieties (Figure 2) have been reported as inhibitors of steroid sulfatase enzyme [40], an important biological target involved in human breast carcinoma [41]. To the best of our knowledge, to date, only a few studies about the combination of stilbene core linked to sulfonamide or sulfonate bridge have been reported for the rational design of aromatase inhibitors. In 2010, a series of five organic and inorganic sulfated RSV metabolites were synthesized and assessed for their activities associated with chemo-preventive properties, such as aromatase inhibition [42]. In 2017, Rasala Mahesh described a study conducted on a series of combretastatin-sulfonamide and sulfonate conjugates as highly potent tubulin polymerization inhibitors (Figure 2) [43].

**Figure 3:**
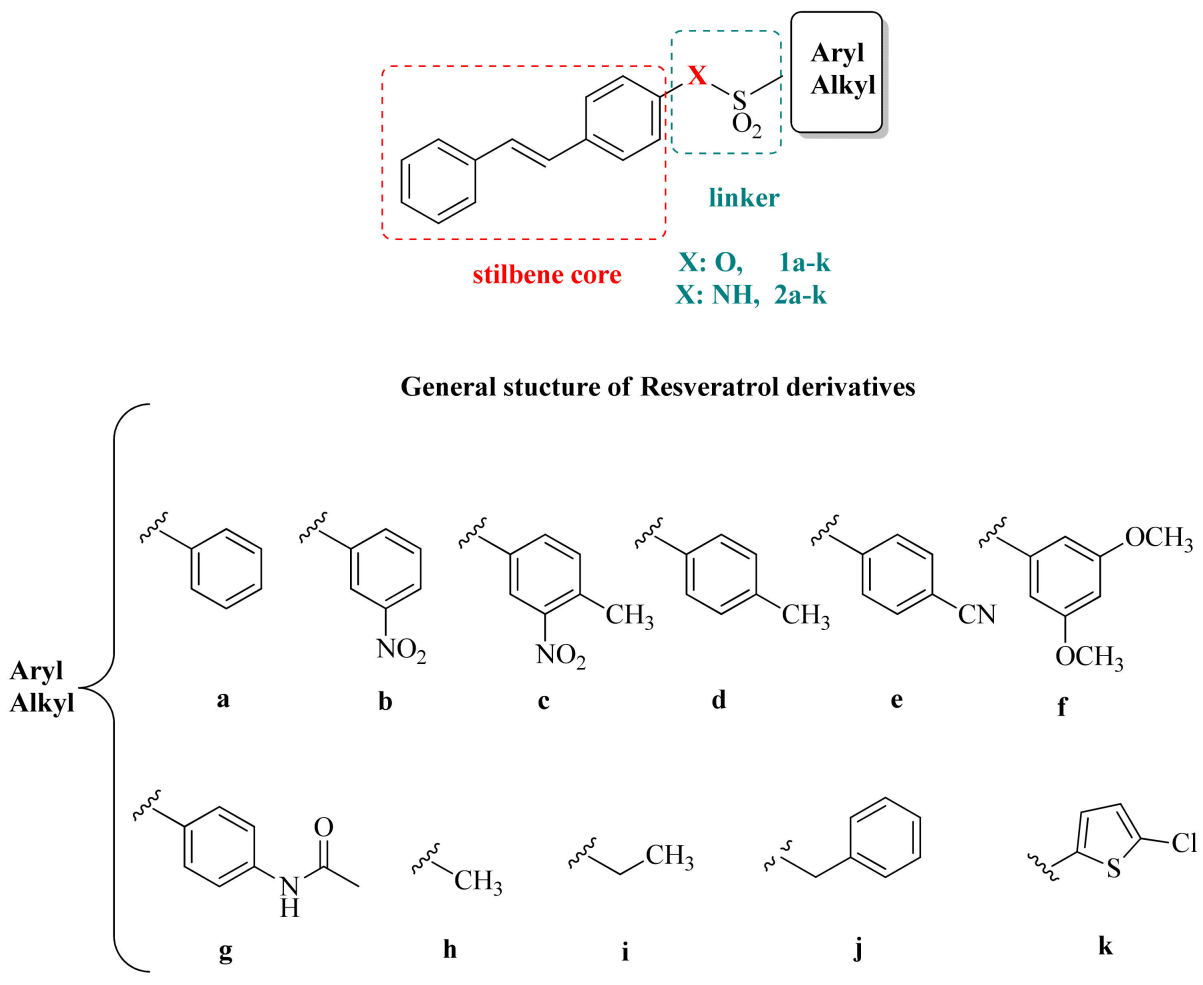
General structure of new sulfonate and sulfonamide derivatives of RSV; X = O, **1a**–**k**; X = NH, **2a**–**k**.

In the past few years, we focused on the synthesis of various series of RSV derivatives. Among them, stilbene hybrids of fibrates showed very interesting activity on many different targets such as the Peroxisome Proliferator-Activated Receptors (PPARs) [44,45,46,47,48]. To enlarge the biological potential of RSV derivatives, and to ameliorate the pharmacokinetic profile, the hydroxy groups in the 3 and 5 positions were substituted with hydrogens and different functional groups were inserted in the 4′-position. These RSV derivatives with low molecular weight and better solubility were tested for their different bioactivities [49,50,51]. Starting from these premises, we herein surmise that the removal the 3,5-dihydroxy moiety and the anchoring a sulfonamide or sulfonate linker on the 4′-OH of RSV could improve its anticancer activity as well as chemical stability. In our extensive study of new aromatase inhibitors [35,36,52,53,54], we designed and synthesized a series of 22 analogues of RSV in which the hydroxy groups in 3 and 5 were substituted by hydrogens and the hydroxy group in 4′ position was replaced by a sulfonate (**1a**–**k**, Figure 3) or a sulfonamide bridge (**2a**–**k**, Figure 3) to an aromatic ring, decorated with substituents covering different steric, hydrophobic, electronic and H-bonding properties or to the bioisostere thienyl ring (**1k** and **2k**) or to short alkyl chains (**1h**–**i** and **2h**–**i**).

These novel RSV derivatives were screened for in vitro aromatase inhibition. The effect on the catalytic activity (percentage of inhibition and IC_50_) of the human aromatase was measured by a fluorescence-based assay using Letrozole as a reference compound. Moreover, the cell viability and cytotoxicity were evaluated using MTT assay and LDH release assay over the MCF7 breast cancer cell line, comparing results with RSV. The interactions between the most active molecules and the catalytic site of the enzyme were analyzed through docking studies. Structure–activity analyses relating the relevance of the hydrophobic and electronic properties on the observed inhibitions were also carried out. A study of stability was conducted on the most active sulfonate compounds in comparison with the corresponding sulfonamides.

## 2. Results and Discussions

### 2.1. Chemistry

**Scheme 1:**
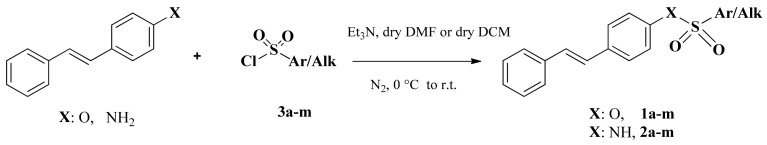
Synthesis of sulfonates **1a**–**k** and sulfonamides **2a**–**k**. Reagents and conditions: 4-hydroxystilbene or 4-aminostilbene, proper sulfonyl chlorides **3a**–**m**, triethylamine, dry DMF for sulfonates or dry DCM for sulfonamides, N_2_, 0 °C → r.t.

The synthesis of compounds **1a**–**k** and **2a**–**k** was made by the reaction of the proper commercial 4-hydroxystilbene (sulfonates **1a**–**k**) or 4-aminostilbene (sulfonamides **2a**–**k**) and the commercial aryl or alkylsulfonylchloride, in the presence of triethylamine in dry DMF for sulfonates, or dry DCM for sulfonamides, at 0 °C and in an anhydrous atmosphere. The mixture was allowed to reach room temperature for 6–12 h. The purification by liquid chromatography gave the purified compounds **1a**–**k** and **2a**–**k**. The reaction route is presented in Scheme 1.

### 2.2. Aromatase Inhibition Studies

The in vitro anti-aromatase activity of the compounds **1a**–**k** and **2a**–**k** was evaluated using a commercial fluorimetric assay kit (Aromatase-CYP19A Inhibitor Screening kit, BioVIsion) with Letrozole (IC_50_ = 1.9 nM) as reference drug, based on methods described elsewhere [55].

**Figure 4:**
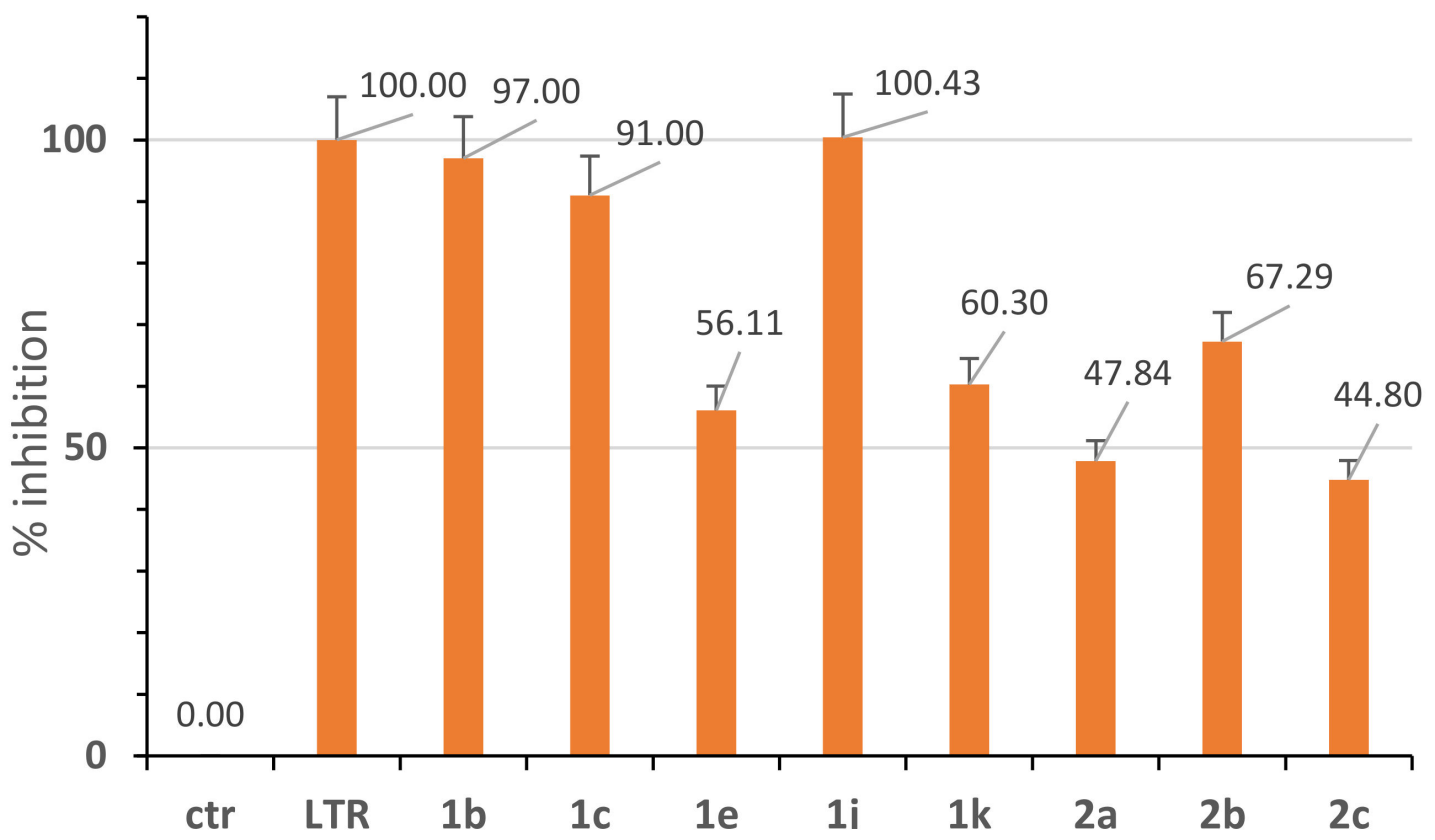
Aromatase inhibition results for new RSV derivatives. Letrozole, **1a**–**k** and **2a**–**k** were tested at 1 µM. Measurements are referred to the means ± standard deviations (S.D.) of three replicates.

New synthesized compounds were dissolved in acetonitrile (final concentration 1 µM), and the results were compared to Letrozole (100% aromatase inhibition) at the same concentration, and in the absence of an inhibitor (0% aromatase inhibition). The activity was measured each minute for 60 min and the value obtained for each compound after 10 min was chosen to calculate the percentage of the aromatase inhibition. Only compounds displaying inhibition percentages higher than 40 are reported in Figure 4.

**Figure 5:**
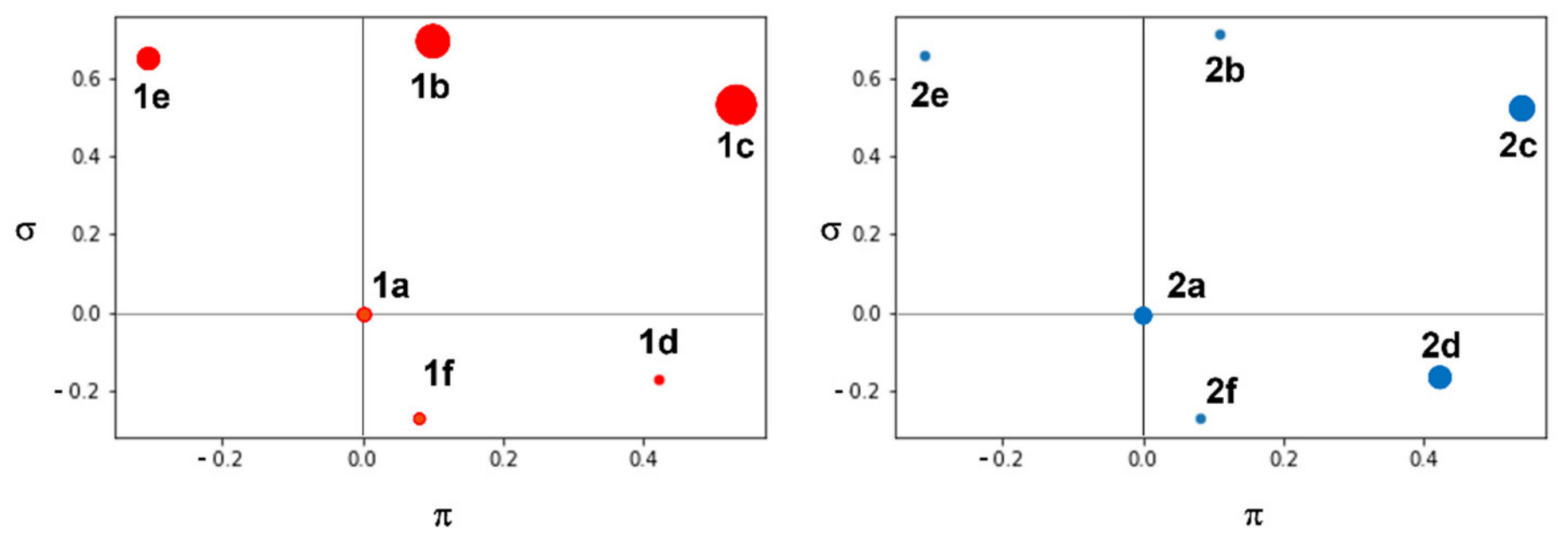
Parallel Craig plots for most active sulfonate (red circles) and sulfonamide (blue circles) derivatives on **left**- and **right**-hand sides, respectively. The circles are sized in proportion to the percentage of in vitro human aromatase inhibition.

On this basis, some preliminary structure–activity relationships can be derived. In general, sulfonates provided a better inhibition of the target enzyme compared to the sulfonamide bioisosteres: the number of active sulfonates is higher than sulfonamide ones. Generally, the presence of a substituted aromatic ring promoted the inhibition with respect to derivatives with methyl and ethyl substituents (**1h**–**i**, **2h**–**i**), capable only of rather limited inhibition. The effect of thienyl ring is different for the two series of compounds. The activity of the sulfonate **1k** is superior respect to the analogue **1a** that displays a very low activity but for the corresponding sulfonamide, the trend is inverse, making **2k** inactive with respect to **2a**. As far as aryl derivatives (**1a**–**g** and **2a**–**g**) are concerned, electron-withdrawing groups tend to award sulfonates compared to sulfonamides. For instance, the nitro group at the meta position determined a very high activity (compounds **1b** and **1c**). To a less extent, the inhibition potential of sulfonamides could benefit electron-donating groups, for instance, the methyl group at the para-position (**2c**–**d**). In this scenario, the cumulative contributions of substituents on the phenyl ring were then investigated by calculating Hammett σ and Hansch π constants in the attempt to provide a molecular rationale behind the different effects on bioactivity for the two sets of aryl derivatives (**1a**–**g** vs. **2a**–**g**) [56]. For the sake of clarity, it should be said that positive/negative Hammett σ constants account for electron-withdrawing/electron-donating effects while positive/negative Hansch π constants quantify the hydrophobic/hydrophilic contents. To better relate electronic and hydrophobic effects of the sulfonates and sulfonamides with the percentage of in vitro human aromatase inhibition, two parallel Craig plots were reported [57]. As far as sulfonate derivatives were concerned, the biological activity was mostly related to the presence of electron-withdrawing groups as shown on the left-hand side of Figure 5. As far as sulfonamide derivatives were concerned, the biological activity increased at the increment of the hydrophobicity content and irrespective of the electronic properties as shown on the right-hand side of Figure 5.

Based on the percentages of inhibition data, compounds **1b**, **1c**, and **1j** were prioritized for calculating the IC_50_ values. Concentration–response curves were calculated in the range 0.01–100 µM and the values are reported in Table 1. In this respect, we detected values of inhibition potency in a narrow low micromolar range, with IC_50_ values from 2.21 to 3.17 μM (Table 1). Although they do not reach the level of inhibition of the clinically used Letrozole, these compounds showed a marked improvement of the inhibitory activity on aromatase enzyme when compared to RSV [25], confirming their ability to inhibit the aromatase enzyme.

### 2.3. Viability and Cytotoxicity Assay

**Figure 6:**
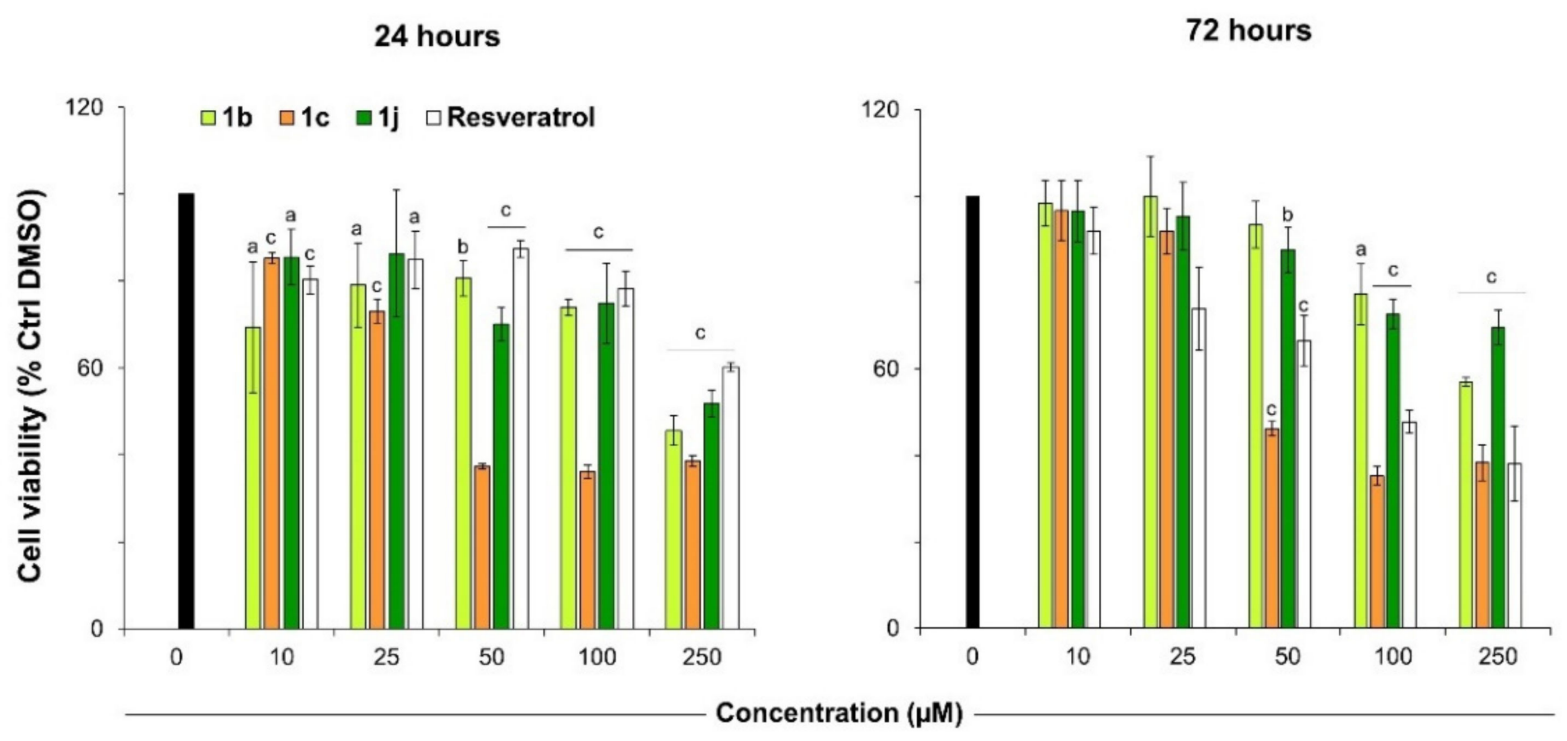
Cell metabolic activity of MCF7 human breast cancer cells in the presence of increasing concentrations of compounds **1b**, **1c**, **1j**, and **RSV**. Data shown are the means ± S.D. of three replicates and are expressed as percentages of cultures in the presence of vehicle (DMSO) set as 100%. a = *p* < 0.01; b = *p* < 0.001; c = *p* < 0.0001 between compounds and DMSO.

The most active compounds **1b**, **1c**, and **1j** underwent an in vitro evaluation on the MCF7 breast cancer cell line and normal human skin fibroblast (HFF-1) [58]. Cell metabolic activity (MTT assay) was evaluated on both the cell lines in the presence of increasing concentrations of compounds to demonstrate selectivity. The compounds were then biologically characterized in terms of cytotoxicity occurrence (LDH released) on MCF7 cells. RSV was included in the biological analyses on MCF7 cells as the reference compound. After 24 h of treatment, a dose-dependent decrease in cell metabolic activity can be registered for all the compounds with respect to DMSO alone. Notably, all compounds are already effective at the concentration of 10 µM, displaying a weak but significant reduction (around 20%) in terms of cell metabolic activity, like RSV (Figure 6). In details, compound **1c** shows the best performance in terms of metabolic activity reduction, being the relative percentages significantly lower than the sample with only the DMSO control and the other compounds of the series already at the dose of 50 µM (around 60% of metabolically active cells) compared to DMSO alone (100%) and RSV, slightly active at this concentration. Similar results were obtained also after 72 h of treatment. In this case, RSV shows a linear dose-dependent activity, whereas **1c** provides a more pronounced cytotoxicity with respect to RSV that showed similar behavior only at 250 μM and after 72 h of treatment.

The IC_50_ of **1b**, **1c**, and **1j** was measured in MCF7 cell line. The compounds were tested in the range of concentrations between 0 to 500 µM (500 µM not shown) and the IC_50_ was assessed at 24 and 72 h (Table 1). Accordingly, the IC_50_ calculated for compound **1c** is 29.23 µM after 24 h, 159.3 µM for compound **1b**, and even higher than 250 µM for compound **1j** and the reference RSV. Compound **1c** retained the same trend after longer exposure (72 h, IC_50_ of 39.95 µM) confirming **1c** as the most effective compound of the series.

Notably, **1c** displays the best MTT profiles, with extremely cytotoxicity already at the dose of 50 µM. It is plausible to assume that compound **1c** exerts the best performance on cells having the lowest IC_50_ after 24 and 72 h, with respect to the other compounds and RSV. On the contrary, compound **1j** seems to bind the aromatase enzyme better than **1c**, showing the best percentage of inhibition (100.43%) and the lower IC_50_ (2.21 µM), but this behavior is not retained in the cells. This fact could be referred to as chemoresistance mechanisms [59,60].

**Figure 7:**
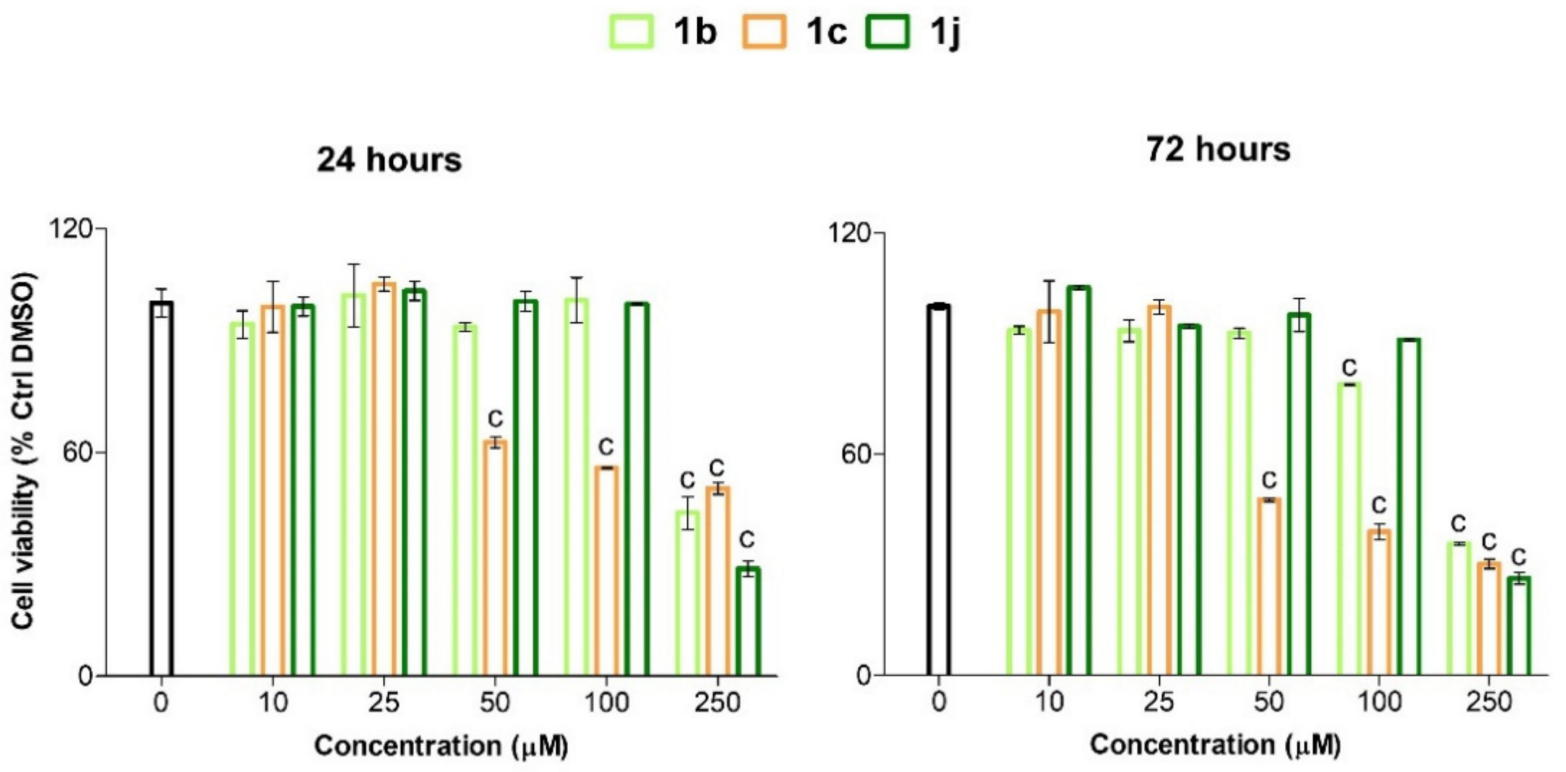
Cell metabolic activity of human skin fibroblasts (HFF-1) in the presence of increasing concentrations of compounds **1b**, **1c**, and **1j**. Data shown are the means ± S.D. of three replicates and are expressed as percentages of cultures in the presence of vehicle (DMSO) set as 100%. a = *p* < 0.01; b = *p* < 0.001; c = *p* < 0.0001 between compounds and DMSO.

A dose-dependent activity for the best compound **1c** was registered in HFF-1 cells but it reached a significant reduction in viability only at the maximum concentration and after 72 h of treatment. It should be also noted that **1c** shows the best selectivity index (SI) over normal fibroblasts assessed at 1.82 after 24 h, which it seems to not be able to maintain so efficiently after longer exposures (Figure 7). It has been widely reported that potent aromatase inhibitors, such as Letrozole, frequently cause adverse effects in the skin compartment. For instance, the aromatase inhibition can adversely affect cutaneous wound healing in the acute setting [61]. Further drug design studies on **1c** can, therefore, be conducted to improve this aspect.

**Figure 8:**
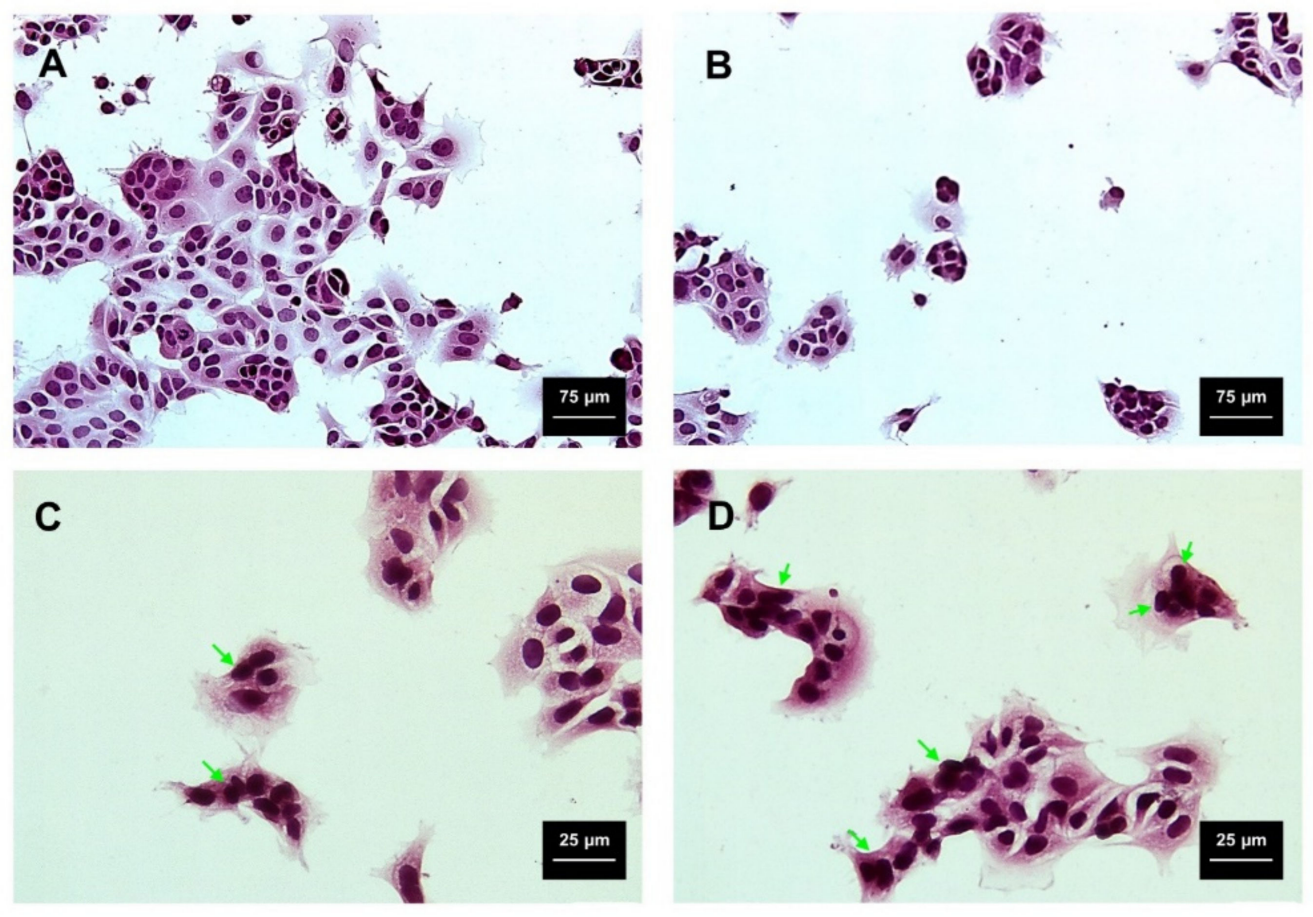
Hematoxylin/eosin (H&E) staining of MCF7 cells in the presence of compound **1c** at 50 µM after 72 h. **A** = 0 µM; **B**, **C**, and **D** = 50 µM compound **1c**. Magnification 200× (**A**,**B**) and 400× (**C**,**D**). The bar scales represent 1 cm = 75 µm and 25 µm, respectively. Cell nuclei are stained in purple, and the cytoplasm fraction is pink-stained. Green arrows indicate chromatin condensation.

The effectiveness on MCF7 cells of compound **1c** over the other compounds is confirmed by the hematoxylin/eosin staining (H&E) performed after 72 h of treatment at 50 µM. As shown in Figure 8, the cell number is dramatically decreased in the presence of the **1c** (panel **B**) with respect to cells treated with only DMSO (panel **A**). At a higher magnification (panels **C** and **D**), MCF7 cells show chromatin condensation (green arrows), as a typical sign of the initiation of cell death. These data confirm the ability of **1c** to reduce the vitality of cancer cells.

**Figure 9:**
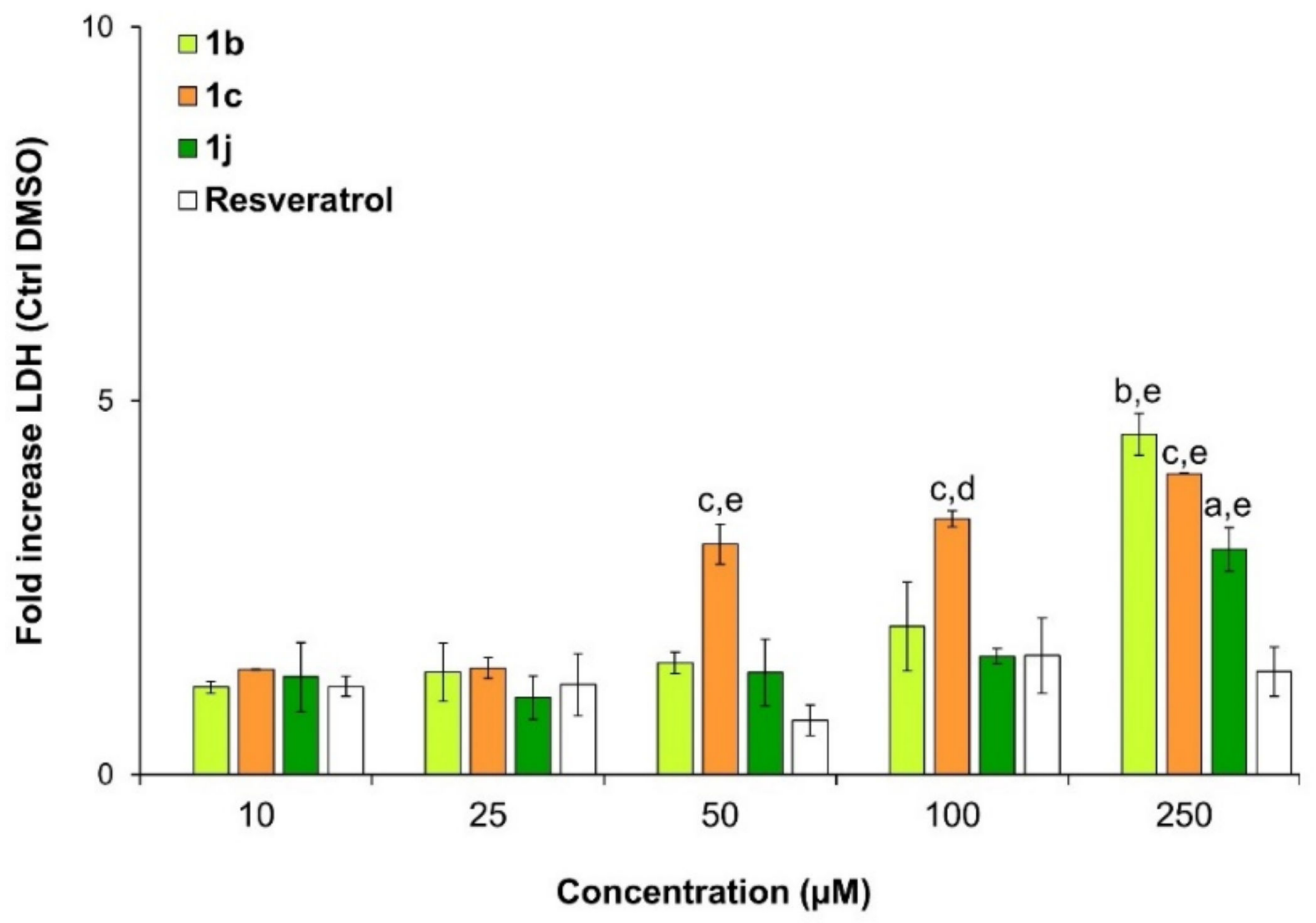
LDH released by MCF7 human breast cancer cells in the presence of increasing concentrations of compounds **1b**, **1c**, **1j**, and RSV after 24 h. Data shown are the means ± S.D. of three replicates and are expressed as the fold increase on the LDH released by cultures in the presence of vehicle (DMSO) set as 1 (data not shown). a = *p* < 0.01; b = *p* < 0.001; c = *p* < 0.0001 between compounds and DMSO; d = *p* < 0.01; e = *p* < 0.001 between compounds and RSV at the same concentration.

To assess whether the decrease in metabolic activity is a consequence of cytotoxic occurrence in the presence of compounds **1b**, **1c**, and **1j**, lactate dehydrogenase (LDH) released from MCF7 was measured in response to loading concentrations of 0–250 µM after a 24 h exposure. A significant increase in the LDH released is found in the presence of 50 µM of **1c**, being three folds more of the LDH released from cultures in the presence of vehicle (DMSO) set as 1 (not shown) and significantly higher with respect to RSV and other compounds at the same dose (Figure 9). After this concentration, the LDH released in the presence of **1c** increases in a dose-dependent manner, being almost five folds more than the one related to DMSO at 250 µM. As for compounds **1b** and **1c**, the release of LDH raises only at the highest concentration administered (250 µM). It is, therefore, plausible to assume that all tested compounds exert their anti-proliferative effect on MCF7 cells through cytotoxic induction and **1c** is the most promising compound, being active already at lower doses.

### 2.4. Molecular Docking Studies

**Figure 10:**
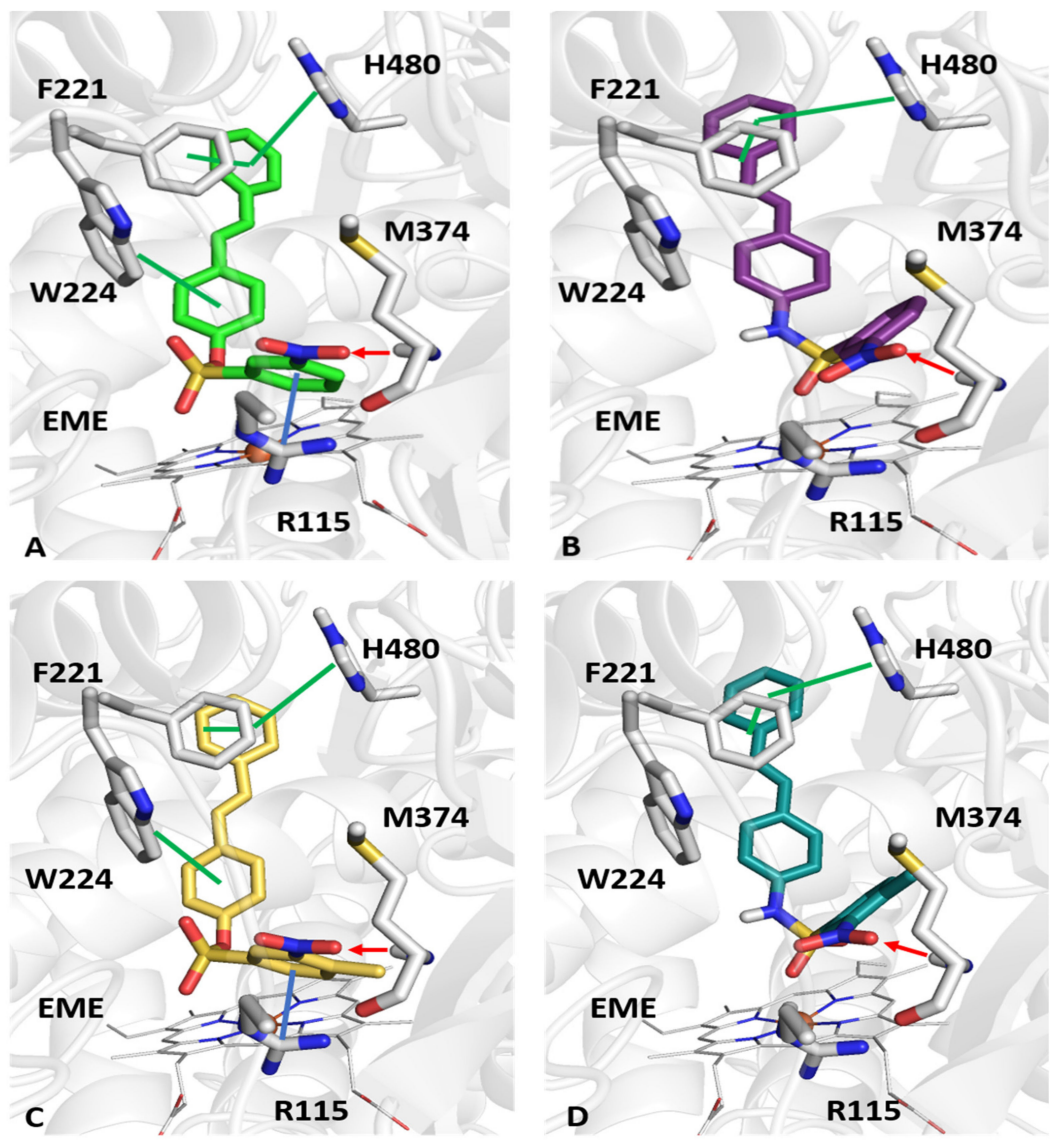
Zoomed in view at the aromatase CYP19A1 binding pocket. Panels (**A**–**D**) show the posing for compounds **1b**, **2b**, **1c**, and **2c**, which are depicted as green, purple, yellow, and deep teal sticks, respectively. π–π contacts and dipole interactions are reported as green and blue lines while hydrogen bonds are represented as red arrows.

Molecular docking analyses were performed to better understand at a molecular level of detail the experimental binding differences observed for sulfonate and sulfonamide derivatives towards aromatase. Our attention was mostly paid to study the differences between **1b** and **2b**, on one side, and the differences between **1c** and **2c**, on the other. First, we observed that similar docking scores were returned after QM docking calculations (i.e., −5.999 kcal/mol and −5.091 kcal/mol for **1b** and **2b**, respectively, and −6.089 kcal/mol and −5.183 kcal/mol for **1c** and **2c**, respectively). Such values reflected to some extent the trend of the in vitro human aromatase inhibition profiles, experimentally determined. As shown in Figure 10, sulfonate derivatives **1b** and **1c** could experience the same interactions. Noteworthy, the stilbene moiety and the phenyl ring of **1b** and **1c** establish π–π contacts with H480, F221, and W224 while the meta-nitro substituent can engage the main chain of M374 through a hydrogen bond. Interestingly, the sulfonate derivatives **1b** and **1c** could make also specific dipole interactions with R115. On the other hand, sulfonamides **2b** and **2c** could make π–π interactions with H480 and F221 by employing the stilbene structure; they establish a hydrogen bond by involving the meta-nitro substituent of the phenyl ring and the backbone of M374. Unlike sulfonates, sulfonamides were unable to properly engage W224 as well as R115, with this being behind their worse scoring values.

Although one of the major differences between sulfonamide and sulfonate derivatives is indeed the NH moiety, molecular docking was unsuccessful to model this feature as key for molecular recognition, at least considering the panel of the available data. This was also anticipated by the Craig plot (right-hand side) showing that the variation of electronic properties of sulfonamides cannot be causatively related to change of the observed biological activity.

### 2.5. Chemical Stability

To obtain information that would facilitate the subsequent development of these RSV derivatives, their chemical stability was evaluated. Aqueous solutions of the more active sulfonates **1b**–**c** and **1j** were prepared at 37 °C at different pH values, which mimic in vivo conditions. The results were compared with corresponding sulfonamides **2b**–**c** and **2j**. The chemical stability was evaluated developing an RP-nano-LC method. Advantages including low consumption of mobile and stationary phases and minimal sample volumes make this analytical tool more cost-effective and eco-friendlier with respect to the conventional HPLC. In addition, the reduction in particle diameter and the use of short chromatographic capillary column allow rapid analyses in isocratic elution mode. This can lead to saving analysis time since the column does not require a conditioning time between the chromatographic runs.

Due to the low solubility of some compounds in pure water, the study was performed in an aqueous solution containing ACN/H_2_O as mobile phase. These conditions led to the chromatographic separation of each compound within 6 min. The chemical stability was evaluated dissolving the studied compounds at a final concentration of 100 µg/mL with a solution of 10 mM hydrochloric acid buffer (pH 2.0, simulating a non-enzymatic gastric fluid), 10 mM phosphate buffer (pH 7.4, neutral conditions simulating the plasma), and 0.1 mM sodium hydroxide solution (pH 9, simulating a non-enzymatic intestinal fluid). The analyses were performed with a nano-LC apparatus at appropriate intervals for 72 h, maintaining the prepared samples at a temperature of 37 °C.

**Figure 11:**
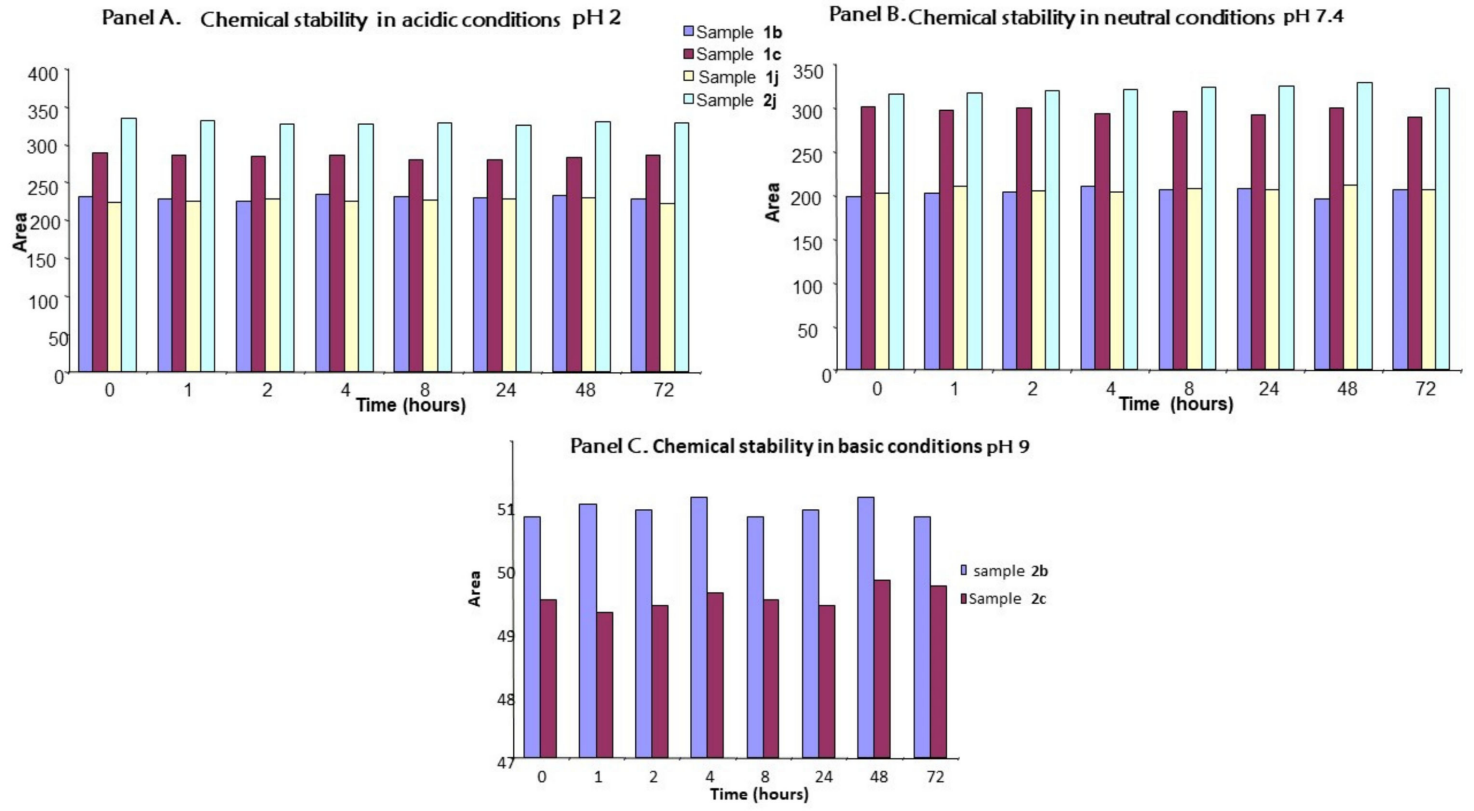
Area under the curves for compounds **1b**–**c**, **2b**–**c**, **1j**, and **2j**, in acidic (panel **A**), neutral (panel **B**), and basic (panel **C**) conditions.

Under acid-induced stress degradation, the samples **1b**–**c**, **1j**, and **2j** were stable over the entire range of time as shown by the value of the area under the curve (AUC) (Figure 11, panel A), while **2b**–**c** showed a degradation peak a few hours after the first injection (time h = 0, data not shown). Under neutral conditions, the same behavior was observed (Figure 11, panel B), confirming the chemical stability of **1b**–**c**, **1j**, and **2j**, and the partial degradation of **2b**–**c**. Under base-induced stress degradation studies, only **2b**–**c** were stable, obtaining a single chromatographic peak; the stability in the entire range of time is depicted in (Figure 11, panel C). It was found that the compounds **1b**–**c**, **1j**, and **2j** were highly degraded because of their precipitation in the basic solution. This precipitation is likely bound to the less acidic character of these compounds.

## 3. Materials and Methods

### 3.1. Chemistry

All chemical reagents for synthesis were purchased from Aldrich or Fluka, trans-4-hydroxystilbene from Carlo Erba Reagents and were used without further purification. Chemical reactions were monitored by thin-layer chromatography (TLC) on F254 silica gel 60 TLC plates and the analysis of the plates was carried out using a UV lamp 254/365 nm or by iodine vapor. Flash chromatography was performed on silica gel 60 (Merck). Melting points were determined in open capillary tubes on a Buchi apparatus and were uncorrected. Infrared spectra were recorded on a FT-IR 1600 PerkinElmer spectrometer, and ^1^H and ^13^C NMR spectra were recorded on a Varian instrument 300 MHz spectrometer using tetramethylsilane (TMS) as an internal reference, and chemical shifts are reported in ppm parts per million (ppm, δ units). Coupling constants are reported in units of Hertz (Hz). Splitting patterns are designed as s, singlet; d, doublet; t, triplet; q, quartet; dd, double doublet; m, multiplet; b, broad. Elemental analyses were recorded on a PerkinElmer 240 B micro-analyzer, obtaining results within ± 0.4% of the theoretical values. The purity of all compounds was over 95%. The following solvents and reagents have been abbreviated: chloroform (CHCl_3_), dichloromethane (DCM), dimethylformamide (DMF), ethanol (EtOH), ethyl acetate (EtOAc), methanol (MeOH). All reactions were carried out with the use of the standard techniques.

### 3.2. General Method for the Synthesis of Sulfonates ***1a**–**k***

The sulfonates were prepared by adding proper sulfonylchloride (0.6 mmol) in dry DMF (3 mL/mmol) to a solution of the 4-[(*E*)-2-phenylethenyl]phenol (0.1000 mg, 0.50 mmol) and triethylamine (0.6 mmol) in DMF dry (2 mL/mmol) at 0 °C. The mixture was allowed to react at room temperature for 6 to 24 h. After this time, DMF was evaporated in vacuo and the raw material was divided between NaOH 2 N solution (15 mL) and DCM (15 mL × 5 times). The organic phase was dried over sodium sulfate and concentrated at reduced pressure. The crude product was purified over silica gel (eluents CHCl_3_ 100% or a mixture of cyclohexane and EtOAc), as noted for individual sulfonates listed below.

#### 3.2.1. 4-[(E)-2-Phenylvinyl]phenylbenzenesulfonate **1a**

White solid, 51% yield, purified on silica gel, eluent Cyclohexane: EtOAc 7:3; m.p. 127–130 °C; ^1^H NMR (CDCl_3_) δ 6.96 (d, 2H, C*H*_Ar_, *J* = 9.0 Hz), 7.03 (s, 2H, C*H*C*H*), 7.35 (t, 2H, C*H*_Ar_, *J* = 7.2 Hz), 7.41 (d, 2H, C*H*_Ar_, *J* = 8.4 Hz), 7.48 (d, 3H, C*H*_Ar_, *J* = 6.9 Hz), 7.54 (d, 2H, C*H*_Ar_, *J* = 8.4 Hz), 7.68 (t, 1H, C*H*_Ar_, *J* = 5.1 Hz), 7.85 (d, 2H, C*H*_Ar_, *J* = 7.5 Hz); ^13^C NMR (CDCl_3_) δ 122.6, 126.5, 127.0, 127.5, 127.9, 128.5, 128.7, 129.1, 129.8, 134.2, 135.2, 136.4, 136.8, 148.6; IR (KBr) 3064.9, 3018.3, 1501.2, 1449.2, 1200.0, 1152.7 cm^−1^. Calcd for C_20_H_16_O_3_S: C, 71.41; H, 4.79. Found: C, 71.36; H, 4.80 (Figure S1).

#### 3.2.2. 4-[(E)-2-Phenylvinyl]phenyl 3-nitrobenzenesulfonate **1b**

White solid, 48% yield, purified on silica gel, eluent CHCl_3_ 100%; m.p. 152–155 °C; ^1^H NMR (CDCl_3_) δ 6.99 (d, 1H, C*H*_Ar_, *J* = 9.0 Hz), 7.04 (s, 2H, C*H*C*H*), 7.29 (d, 1H, C*H*_Ar_, *J* = 7.2 Hz), 7.36 (t, 2H, C*H*_Ar_, *J* = 7.2 Hz), 7.44 (d, 2H, C*H*_Ar_, *J* = 8.1 Hz), 7.49 (d, 2H, C*H*_Ar_, *J* = 6.9 Hz), 7.77 (t, 1H, C*H*_Ar_, *J* = 8.4 Hz), 8.16 (d, 1H, C*H*_Ar_, *J* = 8.4 Hz), 8.53 (dd, 1H, C*H*_Ar_, *J*_1–2_ = 1.2 Hz, *J*_2–3_ = 7.2 Hz), 8.72 (s, 1H, C*H*_Ar_); ^13^C NMR (CDCl_3_) δ 122.3, 123.7, 126.6, 126.7, 127.8, 128.1, 128.6, 128.7, 130.0, 130.3, 130.6, 133.9, 136.6, 137.0, 148.1, 149.4; IR (KBr) 3095.7, 2925.1, 1537.6, 1367.0, 1186.8 cm^−1^. Calcd for C_20_H_15_NO_5_S: C, 62.98; H, 3.96; N, 3.67. Found: C, 62.74; H, 3.96; N, 3.67.

#### 3.2.3. 4-[(E)-2-Phenylvinyl]phenyl 4-methyl-3-nitrobenzenesulfonate **1c**

Yellow solid, 51% yield, purified on silica gel, eluent Cyclohexane: EtOAc 8:2; m.p. 124–126 °C; (CDCl_3_) δ 2.70 (s, 3H, C*H*_3_), 7.00 (dd, 2H, C*H*C*H*, *J*_1–2_ = 14.4, *J*_2–3_ = 9.0), 7.04 (s, 2H, C*H*_Ar_), 7.36 (t, 3H, C*H*_Ar_, *J* = 7.5 Hz), 7.44 (d, 2H, C*H*_Ar_, *J* = 8.7 Hz), 7.49 (d, 2H, C*H*_Ar_, *J* = 6.9 Hz), 7.53 (d, 1H, C*H*_Ar_, *J* = 13.8 Hz), 7.92 (dd, 1H, C*H*_Ar_, *J*_1–2_ = 1.5 Hz, *J*_2–3_ = 6.6 Hz), 8.45 (d, 1H, C*H*_Ar_, *J* = 1.8 Hz); ^13^C NMR (CDCl_3_) δ 20.8, 122.4, 124.9, 126.2, 126.6, 127.2, 127.7, 128.0, 128.6, 128.7, 130.2, 132.0, 133.9, 136.6, 136.9, 140.2, 148.2; IR (KBr) 3430.3, 3082,7, 2932.7, 1380.4, 1181.7 cm^−1^. Calcd for C_21_H_17_NO_5_S: C, 63.79; H, 4.33; N, 3.54. Found: C, 63.56; H, 4.33; N, 3.55.

#### 3.2.4. 4-[(E)-2-Phenylvinyl]phenyl 4-methylbenzenesulfonate **1d**

White solid, 53% yield, purified on silica gel, eluent CHCl_3_ 100%; m.p. 190–191 °C; ^1^H NMR (CDCl_3_) δ 2.45 (s, 3H, CH_3_), 6.95 (d, 2H, C*H*_Ar_, *J* = 9.0 Hz), 7.03 (s, 2H, C*H*C*H*), 7.32 (q, 5H, *CH*_Ar_, *J*_1–2_ = 7.2 Hz, *J*_2–3_ = 8.1 Hz), 7.40 (d, 2H, C*H*_Ar_, J = 8.7 Hz), 7.48 (d, 2H, C*H*_Ar_, *J* = 6.9 Hz), 7.40 (d, 2H, C*H*_Ar_, *J* = 8.7 Hz), 7.71 (d, 2H, C*H*_Ar_, *J* = 8.4 Hz); ^13^C NMR (CDCl_3_) δ 21.7, 122.6, 126.5, 127.1, 127.4, 127.9, 128.5, 129.7, 129.7, 129.7, 135.9, 136.8, 144.3, 148.7, 163.2; IR (KBr) 3449.3, 2927.6, 1590.7, 1 496.9, 1374.0, 1167.4 cm^−1^. Calcd for C_21_H_18_O_3_S: C, 71.98; H, 5.18. Found: C, 71.71; H, 5.17.

#### 3.2.5. 4-[(E)-2-Phenylvinyl]phenyl 4-cyanobenzenesulfonate **1e**

Light brown solid, 55% yield, purified on silica gel, eluent CHCl_3_ 100%; m.p. 150–151 °C; ^1^H NMR (CDCl_3_) δ 6.95 (d, 2H, C*H*_Ar_, *J* = 9.0 Hz), 7.04 (s, 2H, C*H*C*H*), 7.29 (d, 1H, C*H*_Ar_, *J* = 7.8 Hz), 7.36 (t, 2H, C*H*_Ar_, *J* = 7.2 Hz), 7.44 (d, 2H, C*H*_Ar_, *J* = 6.3 Hz), 7.49 (d, 2H, C*H*_Ar_, *J* = 7.5 Hz), 7.83 (d, 2H, C*H*_Ar_, *J* = 8.4 Hz), 7.96 (d, 2H, C*H*_Ar_, *J* = 8.9 Hz); ^13^C NMR (CDCl_3_) δ 116.8, 118.0, 122.3, 126.7, 126.6, 127.7, 128.1, 127.7, 129.1, 130.3, 132.9, 136.6, 139.0, 139.3, 148.2; IR (neat) 3490.0, 2233.6, 1394.2, 1167.8 cm^−1^. Calcd for C_21_H_15_NO_3_S: C, 69.79; H, 4.18; N, 3.88. Found: C, 69.68; H, 4.15; N, 3.87.

#### 3.2.6. 4-[(E)-2-Phenylvinyl]phenyl 4-(acetylamino)benzenesulfonate **1f**

White solid, 38% yield, purified on silica gel, eluent Cyclohexane: EtOAc 3:7; m.p. 160–162 °C; ^1^H NMR (Acetone-d6) δ 2.11 (s, 3 H), 12.04 (d, 2 H, C*H*_Ar_, *J* = 8.7 Hz), 12.30 (dd, 2H, C*H*C*H*, *J*_1–2_ = 22.2 Hz, *J*_2–3_ = 16.5 Hz), 12.39 (t, 1 H, C*H*_Ar_, *J* = 9.0 Hz), 12.53 (t, 2 H, C*H*_Ar_, *J* = 6.0 Hz), 12.65 (d, 2 H, C*H*_Ar_, *J* = 9.0 Hz), 12.73 (d, 2 H, C*H*_Ar_, *J* = 9.0 Hz), 13.70 (broad, 1 H, NH); ^13^C NMR (Acetone*_d_*) δ 28.7, 131.5, 132.4, 132.6, 132.8, 133.8, 133.1, 133.4, 133.8, 138.8, 138.9, 142.5, 142.6,148.6, 173.9; IR (KBr) 3016.6, 1592.3, 1512.1, 1257.2 cm^−1^. Calcd for C_22_H_19_NO_4_S: C, 67.16; H, 4.87; N, 3.56. Found: C, 67.02; H, 4.87; N, 3.54.

#### 3.2.7. 4-[(E)-2-Phenylvinyl]phenyl 2,4-dimethoxybenzenesulfonate **1g**

White solid, 75% yield, purified on silica gel, eluent CHCl_3_ 100%; m.p. 141–142 °C; ^1^H NMR (CDCl_3_) δ 3.84 (s, 3H, C*H*_3_), 3.96 (s, 3H, C*H*_3_), 7.41 (d, 1H, C*H*_Ar_, *J* = 9.0 Hz), 7.49 (d, 1H, C*H*_Ar_, *J* = 1.8 Hz), 7.97 (s, 2H, C*H*C*H*), 8.02 (d, 2H, C*H*_Ar_, *J* = 8.1 Hz), 8.21 (d, H, C*H*_Ar_, *J* = 7.5 Hz), 8.30 (t, 2H, C*H*_Ar_, *J* = 7.5 Hz), 8.35 (d, 3H, C*H*_Ar_, *J* = 8.7 Hz), 8.42 (d, 2H, C*H*_Ar_, *J* = 8.1 Hz), 8.65 (d, 1H, C*H*_Ar_, *J* = 8.7 Hz); ^13^C NMR (CDCl_3_) δ 56.7, 57.2, 100.3, 105.3, 123.3, 127.1, 127.4, 128.2, 128.3, 128.8, 129.6, 130.4, 134.8, 137.0, 137.8, 150.0, 160.2, 167.0; IR (KBr) 3372.0, 2835.6, 1599.8, 1215.8 cm^−1^. Calcd for C_22_H_20_O_5_S: C, 66.65; H, 5.08. Found: C, 66.54; H, 5.07.

#### 3.2.8. 4-[(E)-2-Phenylvinyl]phenylmethanesulfonate **1h**

White solid, 94% yield, purified on silica gel, eluent CHCl_3_ 100%; m.p. 179–180 °C; ^1^H NMR (CDCl_3_) δ 3.15 (s, 3H, C*H*_3_), 6.28 (s, 2H, C*H*C*H*), 6.38 (dd, 3H, C*H*_Ar_, *J*_1–2_ = 1.8 Hz, *J*_2–3_ = 8.7 Hz), 6.57 (t, 2H, C*H*_Ar_, *J*_1–2_ = 7.2 Hz), 6.73 (t, 4H, C*H*_Ar_, *J*_1–2_ = 9.0 Hz); ^13^C NMR (CDCl_3_) δ 36.55, 121.45, 125.82, 126.18, 127.07, 127.22, 127.95, 129.23, 136.01, 147.5; IR (neat) 2938.1, 1499.9, 1367.9, 1174.4 cm^−1^. Calcd for C_15_H_14_O_3_S: C, 65.67; H, 5.16. Found: C, 65.53; H, 5.15.

#### 3.2.9. 4-[(E)-2-Phenylvinyl]phenyl ethanesulfonate **1i**

White solid, 61% yield, purified on silica gel, eluent CHCl_3_ 100%; m.p. 112–114 °C; ^1^H NMR (CDCl_3_) δ 1.55 (t, 3H, C*H*_3_, *J* = 7.5 Hz), 3.29 (q, 2H, C*H*_2_, *J*_1–2_ = 7.5 Hz, *J*_2–3_ = 7.2 Hz), 7.08 (s, 2H, C*H*C*H*), 7.28 (d, 3H, C*H*_Ar_, *J* = 1.8 Hz), 7.37 (t, 2H, C*H*_Ar_, *J*_1–2_ = 7.2 Hz), 7.52 (t, 4H, C*H*_Ar_, *J* = 9.0 Hz); ^13^C NMR (CDCl_3_) δ 8.27, 45.03, 122.23, 126.59, 127.05, 127.80, 127.98, 128.75, 129.89, 136.56, 136.85, 148.2; IR (KBr) 2991.2, 1359.4, 1198.2, 1173.2, 1151.2 cm^−1^. Calcd for C_16_H_16_O_3_S: C, 66.64; H, 5.59. Found: C, 66.53; H, 5.59.

#### 3.2.10. 4-[(E)-2-Phenylvinyl]phenyl phenylmethanesulfonate **1j**

White solid, 33% yield, purified on silica gel, eluent Cyclohexane: EtOAc 7:3; m.p. 139–140 °C; ^1^H NMR (CDCl_3_) δ 4.51 (s, 2H, C*H*_2_), 7.06 (s, 2H, C*H*C*H*), 7.11 (d, 2H, C*H*_2_, *J* = 8.7 Hz), 7.37–7.39 (m, 2H, C*H*_Ar_), 7.41–7.52 (m, 10H, C*H*_Ar_); ^13^C NMR (CDCl_3_) δ 56.74, 122.28, 126.59, 127.16, 127.75, 127.98, 128.76, 129.04, 129.34, 129.84, 129.89, 130.90, 136.50, 136.83, 148.3; IR (KBr) 3353.5, 3288.5, 3026.4, 1513.8, 1391.3 cm^−1^. Calcd for C_21_H_18_O_3_S: C, 71.98; H, 5.18. Found: C, 71.89; H, 5.18.

#### 3.2.11. 4-[(E)-2-Phenylvinyl]phenyl 5-chlorothiophene-2-sulfonate **1k**

White solid, 60 % yield, purified on silica gel, eluent Cyclohexane: EtOAc 8:2; m.p. 160–162 °C; ^1^H NMR (CDCl_3_) δ 6.99 (d, 2H, C*H*_Ar_, *J* = 3.0), 7.11 (dd, 2H, C*H*C*H*, *J*_1–2_ = 17.4, *J*_2–3_ = 8.7), 7.12 (d, 2H, C*H*_Ar_, *J* = 7.8), 7.38 (t, 2H, C*H*_Ar_, *J* = 7.8), 7.51–7.55 (m, 3H, C*H*_Ar_), 7.57 (d 2H, C*H*_Ar_, *J* = 4.2); ^13^C NMR (CDCl_3_) δ 122.41, 126.61, 126.66, 126.91, 126.9, 128.06, 128.76, 130.12, 135.08, 136.75, 136.89, 139.61, 148.55. Calcd for C_18_H_13_ClO_3_S_2_: C, 57.36; H, 3.48. Found: C, 57.49; H, 3.45.

### 3.3. General Method for the Synthesis of Sulfonamides ***2a**–**k***

To a solution of 4-[(E)-2-phenylethenyl]aniline (96.6 mg, 0.51 mmol) and Et_3_N (0.61 mmol) in dry DCM (3 mL/mmol), the suitable sulfonylchloride was slowly added (0.61 mmol) at 0 °C and in nitrogen atmosphere. The mixture was allowed to react at room temperature and after 22–26 h, the mixture was quenched with water (5 mL), the organic solvent was evaporated in vacuo, and the raw material was divided between brine (15 mL) and DCM (15 mL × 5). The organic phase was dried over anhydrous Na_2_SO_4_. The crude product was purified by flash chromatography on silica gel or preparative TLC as noted for individual sulfonamides listed below.

#### 3.3.1. N-{4-[(E)-2-Phenylvinyl]phenyl}benzenesulfonamide **2a**

White solid, 45% yield, purified on silica gel, eluent Cyclohexane: EtOAc 6:4; m.p. 160–162 °C; ^1^H NMR (CDCl_3_) δ 2.09 (broad, ΝH) 7.59 (d, 1H, C*H*_Ar_, *J* = 8.1 Hz), 7.70 (dd, 2H, C*H*C*H*, *J*_2–3_ = 5.5 Hz, *J*_1–2_ = 17.1 Hz), 7.97 (t, 2H, C*H*_Ar_, *J* = 6.9 Hz), 8.5 (d, 3H, C*H*_Ar_, *J* = 10.2 Hz), 8.15 (t, 4H, C*H*_Ar_, *J* = 7.8 Hz), 8.28 (t, 1H, C*H*_Ar_, *J* = 7.8 Hz), 8.55 (d, 3H, C*H*_Ar_, *J* = 7.5 Hz); ^13^C NMR (CDCl_3_) δ 122.6, 126.5, 127.0, 127.4, 127.9, 128.5, 128.7, 129.1, 129.8, 134.2, 135.3, 136.4, 136.8, 140.0; IR (KBr): 3064.9, 3018.3, 1501.2, 1449.2, 1200.0, 1152.7 cm^−1^. Calcd for C_20_H_17_NO_2_S: C, 71.62; H, 5.11; N, 4.18. Found: C, 71.50; H, 5.12; N, 4.18.

#### 3.3.2. 3-Nitro-N-{4-[(E)-2-phenylvinyl]phenyl}benzenesulfonamide **2b**

White solid, 38%, purified on silica gel, eluent Cyclohexane: EtOAc 7:3; m.p. 210–211 °C; ^1^H NMR (CDCl_3_) δ 6.98 (d, 1H, C*H*_Ar_, *J* = 9.0 Hz), 7.04 (s, 2H, C*H*C*H*), 7.29 (d, 1H, C*H*_Ar_, *J* = 7.2 Hz), 7.38 (t, 2H, C*H*_Ar_, *J* = 7.2 Hz), 7.42 (d, 2H, C*H*_Ar_, *J* = 8.1 Hz), 7.49 (d, 2H, C*H*_Ar_, *J* = 6.9) Hz, 7.77 (t, 1H, C*H*_Ar_, *J* = 8.4 Hz), 8.15 (d, 1H, C*H*_Ar_, *J* = 8.4 Hz), 8.52 (dd, 1H, C*H*_Ar_, *J*_1–2_ = 1.2 Hz, *J*_2–3_ = 7.2 Hz), 8.72 (s, 1H, C*H*_Ar_); ^13^C NMR (CDCl_3_) δ 122.3, 123.7, 126.6, 126.7, 127.8, 128.1, 128.6, 128.7, 130.0, 130.3, 130.6, 133.9, 136.6, 137.0, 137.4, 148.1; IR (KBr) 3109.0, 2919.2, 1534.6, 1387.3, 1176.7 cm^−1^. Calcd for C_20_H_16_N_2_O_4_S: C, 63.14; H, 4.24; N, 7.36. Found: C, 63.00; H, 4.26; N, 7.31.

#### 3.3.3. 4-Methyl-3-nitro-N-{4-[(E)-2-phenylvinyl]phenyl}benzenesulfonamide **2c**

White solid, 35% yield, purified on silica gel, eluent Cyclohexane: EtOAc 8:2; m.p. 246–247 °C; ^1^H NMR (CDCl_3_) δ 2.75 (s, 3H, C*H*_3_), 7.03 (d, 2H, C*H*, *J* = 8.1 Hz), 7.13 (dd, 2H, C*H*C*H*, *J*_1–2_ = 15.9 Hz, *J*_2–3_ = 9.9 Hz), 7.25 (s, 2H, C*H*_Ar_), 7.38 (t, 2H, C*H*_Ar_, *J* = 6.9 Hz), 7.53 (t, 3H, C*H*_Ar_, *J* = 8.7 Hz), 7.60 (d, 1H, C*H*_Ar_, *J* = 8.1 Hz), 8.10 (dd, 1H, C*H*_Ar_, *J*_1–2_ = 1.8 Hz, *J*_1–3_ = 8.3 Hz), 8.47 (d, 1H, C*H*_Ar_, *J* = 1.5 Hz); ^13^C NMR (CDCl_3_) δ 21.7, 126.7, 127.0,127.2, 128.1, 128.5, 128.7, 129.5, 130.8, 131.7, 133.1, 136.6, 139.2, 143.0, 144.9; IR (KBr) 3107.7, 2930.0, 1525.7, 1390.2, 1169.4 cm^−1^. Calcd for C_21_H_18_N_2_O_4_S: C, 63.94; H, 4.60; N, 7.10. Found: C, 63.90; H, 4.59; N, 7.09.

#### 3.3.4. 4-Methyl-N-{4-[(E)-2-phenylvinyl]phenyl}benzenesulfonamide **2d**

Orange solid, 35 % yield, purified on silica gel, eluent CHCl_3_ 100%; m.p. 182–183 °C [62]; ^1^H NMR (CDCl_3_) δ 2.47 (s, 3H, C*H*_3_), 7.01 (d, 2H, C*H*_Ar_, *J* = 8.4 Hz), 7.11 (dd, 2H, C*H*, *J*_1–2_ = 16.5 Hz, *J*_2–3_ = 3.9 Hz), 7.25–7.40 (m, 4H, C*H*_Ar_), 7.48 (d, 2H, C*H*_Ar_, *J* = 8.1 Hz), 7.51 (d, 1H, C*H*_Ar_, *J* = 7.5 Hz), 7.83 (d, 4H, C*H*_Ar_, *J* = 8.1 Hz); ^13^C NMR (CDCl_3_) δ 21.7, 126.7, 127.1, 127.2, 128.1, 128.5, 128.7, 129.6, 130.8, 131.8, 133.0, 136.6, 136.7, 139.2, 145.0; IR (KBr) 3028.4, 1379.0, 1161.4 cm^−1^. Calcd for C_21_H_19_NO_2_S: C, 72.18; H, 5.48; N, 4.01. Found: C, 72.17; H, 5.46; N, 4.02.

#### 3.3.5. 4-Cyano-N-{4-[(E)-2-phenylvinyl]phenyl}benzenesulfonamide **2e**

White solid, 48% yield, purified on silica gel, eluent CHCl_3_ 100%; m.p. 238–239 °C; ^1^H NMR (CDCl_3_) δ 6.96 (d, 1H, C*H*_Ar_, *J* = 8.1 Hz), 7.12 (dd, 2H, C*H*C*H*, *J*_1–2_ = 16.5 Hz, *J*_2–3_ = 11.7 Hz), 7.30–7.41 (m, 3H, C*H*_Ar_), 7.52 (d, 3H, C*H*_Ar_, *J* = 8.1 Hz), 7.88 (d, 3H, C*H*_Ar_, *J* = 8.1 Hz), 8.08 (d, 3H, C*H*_Ar_, *J* = 8.4 Hz); ^13^C NMR (CDCl_3_) δ 117.9, 117.9, 126.5, 126.8, 127.5, 128.4, 128.8, 129.2, 131.4, 131.6, 131.7, 132.9, 136.4, 140.2, 142.8; IR (KBr) 3490.0, 2233.6, 1394.2, 1167.8 cm^−1^. Calcd for C_21_H_16_N_2_O_2_S: C, 69.98; H, 4.47; N, 7.77. Found: C, 69.86; H, 4.48; N, 7.78.

#### 3.3.6. N-{4-[({4-[(E)-2-Phenylvinyl]phenyl}amino)sulfonyl]phenyl}acetamide **2f**

White solid, 32% yield, purified on silica gel, eluent CHCl_3_: MeOH 9.5: 0.5; m.p. 230–231 °C; ^1^H NMR (Acetone-d6) δ 2.08 (s, 3H, C*H*_3_), 7.15 (s, 2H, C*H*C*H*), 7.23 (t, 3H, C*H*_Ar_, *J* = 9.0 Hz), 7.34 (t, 2H, C*H*_Ar_, *J* = 6.9 Hz), 7.49 (d, 2H, C*H*_Ar_, *J* = 8.1 Hz), 7.56 (d, 2H, C*H*_Ar_, *J* = 6.9 Hz), 7.74 (t, 4H, C*H*_Ar_, *J*_1–2_ = 8.7 Hz, *J*_2–3_ = 3.6 Hz), 9.02 (s, broad, NH), 9.51 (s, broad, NH); ^13^C NMR (Acetone*_d_*) δ 23.4, 118.4, 120.8, 126.3, 127.2, 127.4, 127.6, 127.8, 128.2, 128.6, 133.7, 137.3, 137.4, 143.5, 143.7, 168.6; IR (KBr) 3345.0, 3135.6, 2922.1, 1682.0, 1592.3, 1508.4 cm^−1^. Calcd for C_22_H_20_N_2_O_3_S: C, 67.33; H, 5.14; N, 7.14. Found: C, 67.23; H, 5.16; N, 7.12.

#### 3.3.7. 2,4-Dimethoxy-N-{4-[(E)-2-phenylvinyl]phenyl}benzenesulfonamide **2g**

Light brown solid, 32% yield, purified on silica gel, eluent CHCl_3_ 100%; m.p. 213–215 °C; ^1^H NMR (CDCl_3_) δ 3.77 (s, 3H, CH_3_), 3.99 (s, 3H, CH_3_), 6.46 (s, 1H, C*H*_Ar_), 6.94 (d, 3H, C*H* + C*H*_Ar_, *J* = 14.7 Hz), 7.04 (d, 2H, C*H*_Ar_, *J* = 9.0 Hz), 7.27 (d, 3H, C*H* + C*H*_Ar_, *J* = 17.1 Hz), 7.32–7.35 (m, 2H, C*H*_Ar_), 7.44 (d, 2H, C*H*_Ar_, *J* = 7.2 Hz), 7.75 (d, 1H, C*H*_Ar_, *J* = 9.0 Hz); ^13^C NMR (CDCl_3_) δ 55.6, 56.4, 99.4, 104.4, 121.3, 126.4, 127.2, 127.5, 127.6, 128.3, 128.6, 132.8, 134.2, 136.1, 137.1, 165.0, 176.5; IR (KBr) 3372.0, 2956.1, 1599.8, 1476.1,1215.8 cm^−1^. Calcd for C_22_H_21_NO_4_S: C, 66.82; H, 5.35; N, 3.54. Found: C, 67,01; H, 5.37; N, 3.53.

#### 3.3.8. N-{4-[(E)-2-Phenylvinyl]phenyl}methanesulfonamide **2h**

White solid, 39% yield, purified on silica gel, eluent CHCl_3_ 100%; m.p. 250–251 °C; ^1^H NMR (CDCl_3_) δ 3.15 (s, 3H, C*H*_3_), 7.08 (s, 2H, C*H*C*H*), 7.27 (dd, 3H, C*H*_Ar_, *J*_1–2_ = 2.4 H, Hz, *J*_2–3_ = 1.8 Hz), 7.37 (t, 2H, C*H*_Ar_, *J*_1–2_ = 7.5 Hz), 7.53 (t, 4H, C*H*_Ar_, *J*_1–2_ = 8.7 Hz); ^13^C NMR (CDCl_3_) δ 42.6, 126.7, 126.9, 127.5, 128.2, 128.7, 130.8, 131.2, 132.0, 139.8; IR (KBr): 3020.1, 2941.2, 1372.4, 1340.7, 1157.6 cm^−1^. Calcd for C_15_H_15_NO_2_S: C, 65.91; H, 5.53; N, 5.12. Found: C, 65.80; H, 5.55; N, 5.12.

#### 3.3.9. N-{4-[(E)-2-Phenylvinyl]phenyl}ethanesulfonamide **2i**

White solid, 35% yield, purified on silica gel, eluent CHCl_3_ 100%; m.p. 230–231 °C; ^1^H NMR (CDCl_3_) δ 1.49 (t, 3H, C*H*_3_, *J* = 7.5 Hz), 1.56 (s, broad, NH), 3.61 (q, 2H, C*H*_2_, *J*_1–2_ = 7.2 Hz, *J*_2–3_ = 7.2 Hz), 7.12 (dd, 2H, C*H*, *J*_1–2_ = 15.9 Hz, *J*_2–3_ = 4.2 Hz), 7.30 (d, 1H, C*H*_Ar_, *J* = 4.5 Hz), 7.38 (t, 4H, *J*_1–2_ = 3.6 Hz, *J*_2–3_ = 4.5 Hz), 7.52 (d, 2H, C*H*_Ar_, *J* = 6.9 Hz), 7.57 (d, 2H, C*H*_Ar_, *J* = 8.1 Hz); ^13^C NMR (CDCl_3_) δ 7.8, 50.0, 126.7, 126.9, 127.3, 128.2, 128.7, 131.0, 131.3, 132.4, 136.6, 139.5; IR (KBr) 3042.1, 2988.8, 1346.6, 1148.3 cm^−1^. Calcd for C_16_H_17_NO_2_S: C, 66.87; H, 5.96; N, 4.87. Found: C, 66.76; H, 5.94; N, 4.88.

#### 3.3.10. 1-Phenyl-N-{4-[(E)-2-phenylvinyl]phenyl}methanesulfonamide **2j**

Yellow solid, 36% yield, purified on silica gel, eluent CHCl_3_ 100%; m.p. 170–171 °C; ^1^H NMR (CDCl_3_) δ 4.35 (s, 2H, C*H*_2_), 6.26 (broad, N*H*), 7.07 (s, 2H, C*H*C*H*), 7.12 (d, 2H, C*H*_Ar_, *J* = 8.1 Hz), 7.27–7.29 (m, 3H, C*H*_Ar_), 7.34–7.37 (m, 5H, C*H*_Ar_), 7.52 (t, 4H, C*H*_Ar_, *J* = 3.0 Hz); ^13^C NMR (CDCl_3_) δ 57.3, 120.1, 126.4, 127.4, 127.6, 127.7, 128.4, 128.6, 128.7, 128.9, 129.0, 130.8, 134.2, 135.9, 137.0; IR (KBr): 3353.5, 3288.5, 3026.4, 1513.8, 1391.3 cm^−1^. Calcd for C_21_H_19_NO_2_S: C, 72.18; H, 5.48; N, 4.01. Found: C, 72.08; H, 5.49; N, 4.01.

#### 3.3.11. 5-Chloro-N-{4-[(E)-2-phenylvinyl]phenyl}thiophene-2-sulfonamide **2k**

White solid, 37% yield, purified by preparative TLC on silica gel, eluent Cyclohexane: EtOAc 7:3; m.p. 200–202 °C; ^1^H NMR (CDCl_3_) δ 1.21 (broad, ΝH) 6.99 (d, 2H, C*H*_Ar_, *J* = 3.0), 7.11 (dd, 2H, C*H*C*H*, *J*_1–2_ = 17.4 Hz, *J*_2–3_ = 8.7 Hz), 7.12 (d, 2H, C*H*_Ar_, *J* = 7.8 Hz), 7.38 (t, 2H, C*H*_Ar_, *J* = 7.8 Hz), 7.51–7.55 (m, 3H, C*H*_Ar_), 7.57 (d 2H, C*H*_Ar_, *J* = 4.2 Hz); ^13^C NMR (CDCl_3_) δ 110.0, 126.7, 126.9, 127.4, 128.3, 128.8, 131.3, 132.1, 135.2, 136.5, 136.6, 139.9, 140.4; IR (KBr): 3028.7, 1947.9, 1391.6, 1158.1 cm^−1^. Calcd for C_18_H_14_ClNO_2_S_2_: C, 57.51; H, 3.75; N, 3.73. Found: C, 57.36; H, 3.75; N, 3.72.

### 3.4. Biological Assays

#### 3.4.1. Aromatase Activity Inhibition Assay and Calculation of the IC_50_

The aromatase inhibitory activity was determined using a commercial fluorimetric assay kit (Aromatase-CYP19A Inhibitor Screening kit, BioVision, Milpitas, CA, USA), as reported elsewhere [35,36]. The assay utilizes a fluorogenic aromatase substrate that is converted into a highly fluorescent metabolite detected in the visible range (Ex/Em 488/527 nm). According to the manufacturer specifications, each test compound and Letrozole were incubated for 10 min at 37 °C to allow test ligands to interact with aromatase. During the incubation, the Aromatase Substrate/NADP+ mixture was prepared, and the reaction was started by adding 30 µL of this solution to each well (aside from the background control), reaching a final reaction volume of 100 µL/well. The fluorescence emission (527 nm) was measured immediately (within 1 min) by means of a fluorometer equipped with a 488 nm filter (GloMax^®^-Multi Detection System, Promega, Madison, WI, USA) and afterwards monitored for 60 min. To determine the IC_50_ values for the best tested inhibitors (**1b**, **1c**, and **1j**), 5X test compound solutions were prepared in a range of concentrations (0.05–500 µM in ACN) to generate a multi-point dose–response curve (0.01–100 µM). The assay was performed in the same experimental conditions used for the initial screening of compounds. Dose–response curves were fitted with GraphPad Prism 5.0 (GraphPad Software, San Diego, CA, USA).

#### 3.4.2. Cell Viability Evaluation

##### Cell Viability Assay (MTT)

Human skin fibroblasts (HFF-1, CVCL_3285) and human breast cancer MCF7 (HTB-22™, CVCL_0031) were purchased from ATCC and maintained in Dulbecco’s modified Eagle’s medium (DMEM) high glucose (EuroClone, Milan, Italy) supplemented with 10% of fetal bovine serum (FBS) and 1% of penicillin/streptomycin (Gibco—Thermo Fisher Scientific, MD, USA).

Cell metabolic activity of HFF-1 and MCF7 cells was assessed by MTT (3-(4,5-Dimethylthiazol-2-yl)-2,5-Diphenyltetrazolium Bromide) test (Sigma Aldrich, Milan, Italy). HFF-1 and MCF7 cells were seeded (0.5 × 104 and 0.1 × 10^5^/well, respectively) in a 96-well tissue culture-treated plate (Falcon^®^, Corning Incorporated, Corning, NY, USA) and incubated in the presence of loading concentrations of compounds **1b**, **1c**, and **1j** (0–250 µM) for 24 and 72 h. After the established exposure times, cells were incubated with MTT (0.5 mg/mL) and the optical density was measured as reported elsewhere [63]. Results were expressed as the percentage of cells in the presence of vehicle (DMSO) set as 100% and each experiment was performed in triplicate (*n* = 3). Concentration–response curves and IC_50_ were fitted and calculated with GraphPad Prism 5.0 (GraphPad Software, San Diego, CA, USA).

##### Cytotoxicity Test (Lactate Dehydrogenase Release)

MCF7 cells were seeded and stimulated as previously described for the MTT test. After the exposure time, cell supernatants were collected, centrifuged at 450× *g* for 4 min, and stored on ice. In order to quantify the cytotoxicity of loading concentrations of compounds **1b**, **1c**, and **1j** (0–250 µM), the CytoTox 96^®^ Non-Radioactive Cytotoxicity Assay (Promega Corporation, Madison, WI, USA) was performed as previously reported [64]. The results were normalized on MTT absorbances and expressed as fold increases on the LDH released by cultures in the presence of vehicle (DMSO) set as 1.

##### Hematoxylin/Eosin Staining

MCF7 cells were seeded (7 × 10^4^ cells/well) in well glasses (Millicell^®^ EZ slide, Merck Millipore, Burlington, MA, USA). After 24 h from seeding, cells were exposed to compound **1c** at 50 µM for 72 h. Next, supernatants were removed, and cells were washed twice with PBS with calcium and magnesium (Euro Clone, Milan, Italy). MCF7 cells were fixed in the presence of *p*-formaldehyde 4% and stained with hematoxylin/eosin as reported elsewhere [65].

##### Statistics

Data are presented as mean values ± standard deviations (S.D.) of three independent experiments. Statistical differences were determined by one-way ANOVA and post hoc Tukey multiple comparison tests using the Prism software (version 5.0, GraphPad Software, San Diego, CA, USA). *p* values < 0.05 were considered statistically significant.

### 3.5. Molecular Modeling

Compounds **1b**–**c** and **2b**–**c** were docked on and the 3D crystal structure of aromatase CYP19A1 available from the Protein Data Bank with the code 3EQM [66]. The protein preparation wizard [67,68] available in the Schrodinger Suite was used for refining side chains, adding missing residues, removing water molecules, and minimizing the protein structure. The Ligprep tool [69] was employed to determine all the possible tautomers and protonation states at physiological pH as well as to generate ring conformations and chiral centers. The sampling grid was positioned on the cognate ligand center of mass, setting the inner and outer boxes of 15 Å × 15 Å × 15 Å and 27 Å × 27 Å × 27 Å, respectively, and the van der Waals scaling factor equal to 0.7. To strengthen the validity of docking protocol, redocking analysis was performed on the cognate ligand within the aromatase binding site. Cognate ligands moved back to the original positions with Root Mean Square Deviations (RMSD) accounting for all the heavy atoms equal to 0.76 Å. The QM-polarized ligand docking [70] was carried out to better address scoring and posing through the calculation of single-point energy for each complex based on semiempirical NDDO methods [71,72]. Such protocol enhances docking accuracy compared to standard docking settings.

### 3.6. Chemical Stability

To evaluate the chemical stability, nano-liquid chromatographic (nano-LC) analyses were carried out using a laboratory assembled instrumentation employing a Spectra System P2000 conventional gradient HPLC pump, a UV-vis on-column detector, Spectra Focus PC1000 (both from Thermo Separation Products, San Jose, CA, USA), and a modified injection valve equipped with an external loop of 50 µL (Enantiosep GmbH, Münster, Germany). Detection wavelength was set at 206 nm, and data were collected using ClarityTM Advanced Chromatography Software (Data Apex Ltd., Prague, Czech Republic).

To reduce the flow rate from µ- to nL/min, the HPLC pump delivering MeOH continuously and the injector were connected to a passive split-flow system. For this purpose, both the HPLC pump and injection valve were joined to a stainless-steel T-piece (Vici, Valco, Houston, TX, USA) by means of 500 µm id stainless steel tubes with lengths of 50 and 5 cm, respectively. The third entrance of the T-piece was connected to the MeOH reservoir of the pump through a fused silica capillary (50 µm id × 50 cm), achieving continuous recycling of the organic solvent. To minimize the dead volume and consequently reduce the band broadening effect, the capillary column was directly inserted into the modified injector, which was used for both sample loading and mobile phase reservoir. Samples and mobile phases were introduced into the capillary column through the injection valve by filling the loop with the sample solutions, switching the valve for the appropriate time, and then flushing the loop with the mobile phase. The analysis started immediately after positioning the injector device in the injection mode. When the mobile phase had to be changed, it was directly introduced into the modified injector, significantly reducing the consumption of organic solvents [73]. In the optimized conditions, the flow rate of the column was about 450 nL/min. Nano-LC experiments were performed in uncoated fused silica capillaries (100 μm internal diameter) from Composite Metal Services, Hallow, UK), packed in our laboratory following the slurry packing procedure as described previously [74]. The capillary column was packed with ChromSpher C18, 3 μm particle size (Varian, Palo Alto, CA, USA) for 15 cm.

Chromatographic conditions and standards preparation were as follows: the nano-LC analyses were performed in the capillary column employing as mobile phase a mixture of ACN/H_2_O (75/15% *v*/*v*) containing a 10 mM sodium hydrocarbonate buffer, pH 8.5. Stock solutions of the compounds **1b**–**c**, **1j**, **2b**–**c**, and **2j** (1 mg/mL) were prepared in ACN.

The chemical stability was evaluated dissolving the studied compounds at a final concentration of 100 µg/mL with a solution of: (a) 10 mM hydrochloric acid buffer (pH 2.0), (acidic conditions, simulating a non-enzymatic gastric fluid); (b) 10 mM phosphate buffer (pH 7.4), (neutral conditions, simulating a non-enzymatic intestinal fluid); (c) 0.1 mM sodium hydroxide solution (pH 9). The analyses were performed with a nano-LC apparatus at appropriate intervals for 72 h, maintaining the prepared samples at a temperature of 37 °C.

## 4. Conclusions

We designed, synthesized, and assayed two series of RSV derivatives for their capability to inhibit aromatase, a key enzyme involved in BC. These compounds join the stilbene core of RSV and aromatic or aliphatic moieties through a sulfonate or a sulfonamide linker. The sulfonates were more effective in aromatase inhibition assays—they provided the highest percentage inhibition values and the highest number of active derivatives. Three of them, compounds **1b**–**c** and **1j**, showed very high enzymatic inhibition, with percentages ranking from 91.00% to 100.00%, compared to the reference drug Letrozole, and a value of IC_50_ in the micromolar range better than the parent compound RSV. Dose-dependent cytotoxic activity on MCF7 and LDH release was experimentally observed. Importantly, 1c is twentyfold more potent than RSV in inhibiting the aromatase and shows better cytotoxic activity towards MCF7 cell line; these data were confirmed by chromatin condensation in the same cells. Molecular docking studies highlighted the putative molecular interactions responsible for inhibition in agreement with experimental data. Structural features that improve the biological activity of these RSV derivatives are different for the two series. In general, the presence of a substituted aromatic ring is essential for the activity with respect to aliphatic or thienyl derivatives. Studies conducted on the sulfonates and the corresponding sulfonamide bioisosteres revealed that the former are most affected by the electronic properties of aromatic substituents, while more lipophilic substituents could improve the inhibitory activity of the latter. The good stability was found in acidic and neutral conditions with rapid and cost-effective nano-LC experiments. The structure–activity relationship reveals a wealth of information for future rational design and the best compounds identified in this study represent promising starting points and could be leads for the development of new drug candidates for BC treatment.

## Figures and Tables

**Table 1:** In vitro human aromatase inhibition profile for **1b**, **1c**, and **1j**.

Cpd		Aromatase IC_50_ (μM) ^a,b^	MCF7 Cells IC_50_ (μM) ^c^	
			24 h	72 h
**1b**	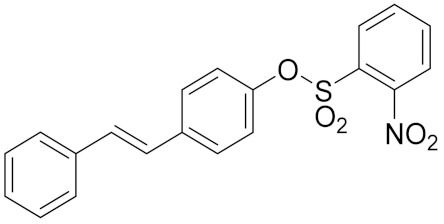	2.51	159.3 ± 9.9	240.3 ± 6.7
**1c**	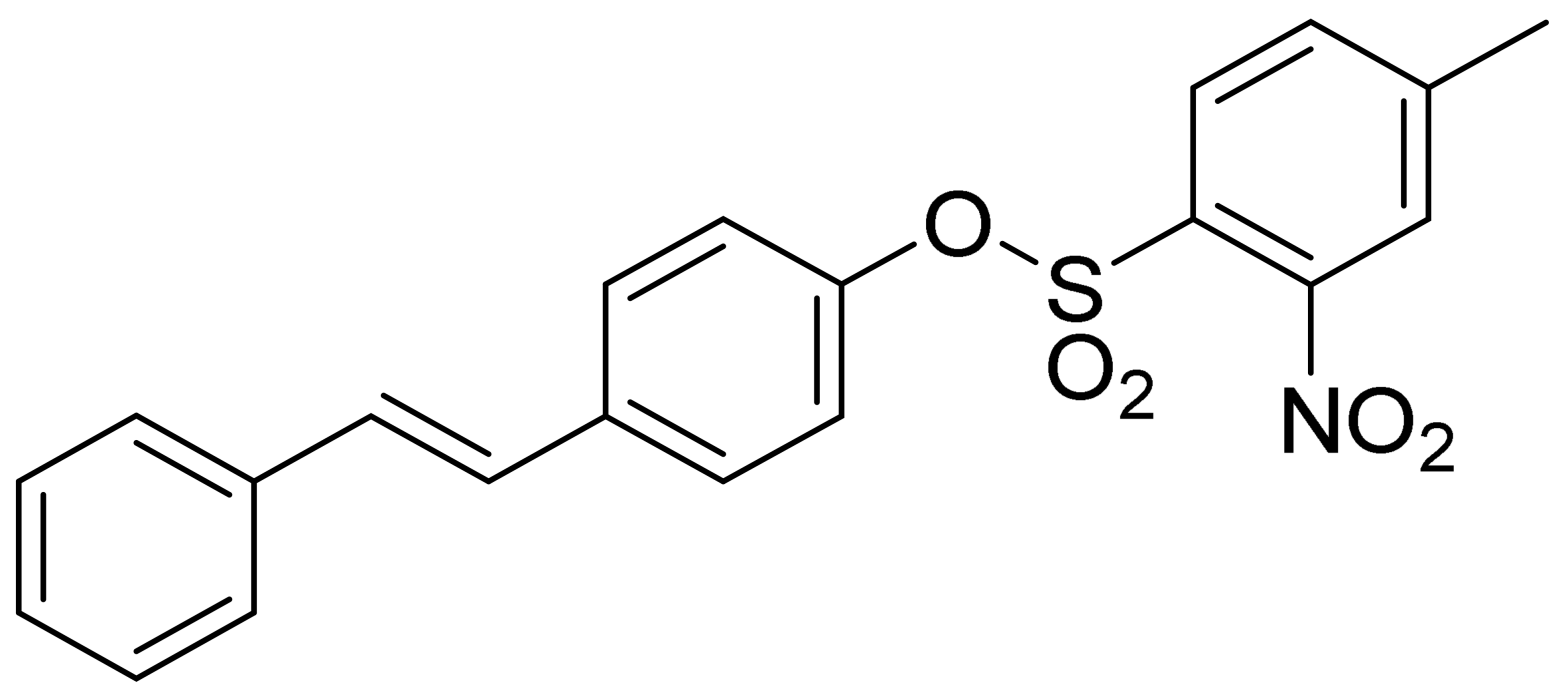	3.17	29.2 ± 1.9	39.9 ± 2.1
**1j**	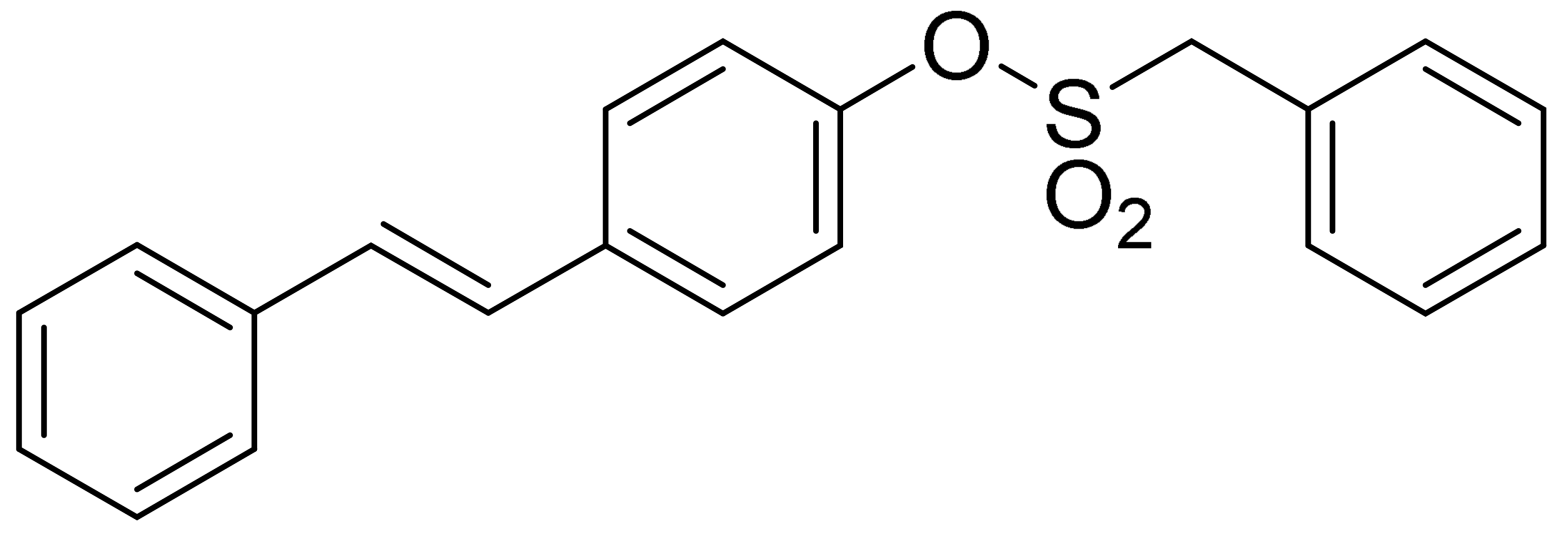	2.21	>250	>250
**RSV**		80 ^d^	>250	83.0 ± 3.8

^a^ Compounds were tested at concentrations from 0.01 to 100 μM; ^b^ the values represent the mean of triplicate determinations ± standard deviation; ^c^ the IC_50_ in MCF7 cell line. The compounds were tested in the range of concentrations between 0 to 500 µM and the IC_50_ assessed at 24 and 72 h; ^d^ [25].

## Data Availability

Data is contained within the article and Supplementary Materials.

## Supplementary Materials

The NMR spectra of compounds **1a**–**k**, **2a**–**k**, raw data for IC_50_ and HPLC chromatograms for compounds **1b**–**c** and **1j** are available online at https://www.mdpi.com/article/10.3390/ph14100984/s1. Figure S1: 4-[(E)-2-phenylvinyl]phenylbenzenesulfonate **1a**; Figure S2: 4-[(E)-2-phenylvinyl]phenyl 3-nitrobenzenesulfonate **1b**; Figure S3: 4-[(E)-2-phenylvinyl]phenyl 4-methyl-2-nitrobenzenesulfonate **1c**; Figure S4: 4-[(E)-2-phenylvinyl]phenyl 4-methylbenzenesulfonate **1d**; Figure S5: 4-[(E)-2-phenylvinyl]phenyl 4-cyanobenzenesulfonate **1e**; Figure S6: 4-[(E)-2-phenylvinyl]phenyl 4-(acetylamino)benzenesulfonate **1f**; Figure S7: 4-[(E)-2-phenylvinyl]phenyl 2,4-dimethoxybenzenesulfonate **1g**; Figure S8: 4-[(E)-2-phenylvinyl]phenylmethanesulfonate **1h**; Figure S9: 4-[(E)-2-phenylvinyl]phenyl ethanesulfonate **1i**; Figure S10: 4-[(E)-2-phenylvinyl]phenyl phenylmethanesulfonate **1j**; Figure S11: 4-[(E)-2-phenylvinyl]phenyl 5-chlorothiophene-2-sulfonate **1k**; Figure S12: N-{4-[(E)-2-phenylvinyl]phenyl}benzenesulfonamide **2a**; Figure S13: 3-nitro-N-{4-[(E)-2-phenylvinyl]phenyl}benzenesulfonamide **2b**; Figure S14: 4-methyl-3-nitro-N-{4-[(E)-2-phenylvinyl]phenyl}benzenesulfonamide **2c**; Figure S15: 4-methyl-N-{4-[(E)-2-phenylvinyl]phenyl}benzenesulfonamide **2d**; Figure S16: 4-cyano-N-{4-[(E)-2-phenylvinyl]phenyl}benzenesulfonamide **2e**; Figure S17: N-{4-[({4-[(E)-2-phenylvinyl]phenyl}amino)sulfonyl]phenyl}acetamide **2f**; Figure S18: 2,4-dimethoxy-N-{4-[(E)-2-phenylvinyl]phenyl}benzenesulfonamide **2g**; Figure S19: N-{4-[(E)-2-phenylvinyl]phenyl}methanesulfonamide **2h**; Figure S20: N-{4-[(E)-2-phenylvinyl]phenyl}ethanesulfonamide **2i**; Figure S21: 1-phenyl-N-{4-[(E)-2-phenylvinyl]phenyl}methanesulfonamide **2j**; Figure S22: 5-chloro-N-{4-[(E)-2-phenylvinyl]phenyl}thiophene-2-sulfonamide **2k**; Figure S23: Chromatograms of the active compounds **1b**–**c** and **1j** in neutral conditions; Table S1: Raw data for the IC50 calculation related to MCF7 cells in the presence of increasing concentrations of compounds **1b**, **1c**, **1j** and Resveratrol for 24 and 72 h.
