# Supplementary material for: Design, Synthesis and Biological Evaluation of Aromatase Inhibitors Based on Sulfonates and Sulfonamides of Resveratrol
Source: Pharmaceuticals (Basel). 2021 Sep 27;14(10):984. doi: 10.3390/ph14100984 (PMC8537897; doi:10.3390/ph14100984)

Design, Synthesis and Biological Evaluation of Aromatase Inhibitors based on Sulfonates and Sulfonamides of Resveratrol

Marialuigia Fantacuzzi[a,§,*], Marialucia Gallorini[b,§], Nicola Gambacorta[c], Alessandra Ammazzalorso [a], Zeineb Aturki[d], Marwa Bahala[b,e], Simone Carradori [a], Letizia Giampietro[a], Cristina Maccallini[a], Amelia Cataldi[b], Orazio Nicolotti[c], Rosa Amoroso[a], Barbara De Filippis[a,*]

[a] *Unit of Medicinal Chemistry, Department of Pharmacy, "G. d'Annunzio" University, Chieti, Italy*

[b] *Unit of Anatomy, Department of Pharmacy, "G. d'Annunzio" University, Chieti, Italy*

[c] *Unit of Medicinal Chemistry, Department of Department of Pharmacy, "A. Moro" University, Bari, Italy*

[d] *Institute for Biological Systems (ISB), Italian National Research Council, Monterotondo, Rome, Italy*

[e] *Department of Pharmaceutical Chemistry, Faculty of Pharmacy, Kafrelsheikh University, Kafr El Sheikh 33516, Egypt*

**Correspondence: B.D.F., barbara.defilippis@unich.it; M.F., marialuigia.fantacuzzi@unich.it.*

§These authors contributed equally to the work

Supplemental Table of Contents

Spectra of compound:

Figure S1 *4-[(E)-2-phenylvinyl]phenylbenzenesulfonate **1a*** *4*

Figure S2 *4-[(E)-2-phenylvinyl]phenyl 3-nitrobenzenesulfonate **1b*** *6*

Figure S3 *4-[(E)-2-phenylvinyl]phenyl 4-methyl-2-nitrobenzenesulfonate **1c*** *8*

Figure S4 *4-[(E)-2-phenylvinyl]phenyl 4-methylbenzenesulfonate **1d*** *10*

Figure S5 *4-[(E)-2-phenylvinyl]phenyl 4-cyanobenzenesulfonate **1e*** *12*

Figure S6 *4-[(E)-2-phenylvinyl]phenyl 4-(acetylamino)benzenesulfonate **1f*** *14*

Figure S7 *4-[(E)-2-phenylvinyl]phenyl 2,4-dimethoxybenzenesulfonate **1g*** *16*

Figure S8 *4-[(E)-2-phenylvinyl]phenylmethanesulfonate **1h*** *18*

Figure S9 *4-[(E)-2-phenylvinyl]phenyl ethanesulfonate* **1i** *20*

Figure S10 *4-[(E)-2-phenylvinyl]phenyl phenylmethanesulfonate* **1j** *22*

Figure S11 *4-[(E)-2-phenylvinyl]phenyl 5-chlorothiophene-2-sulfonate* **1k** *24*

Figure S12 *N-{4-[(E)-2-phenylvinyl]phenyl}benzenesulfonamide* ***2a*** *26*

Figure S13 *3-nitro-N-{4-[(E)-2-phenylvinyl]phenyl}benzenesulfonamide* ***2b*** *28*

Figure S14 *4-methyl-3-nitro-N-{4-[(E)-2-phenylvinyl]phenyl}benzenesulfonamide* ***2c*** *30*

Figure S15 *4-methyl-N-{4-[(E)-2-phenylvinyl]phenyl}benzenesulfonamide* ***2d*** *32*

Figure S16 *4-cyano-N-{4-[(E)-2-phenylvinyl]phenyl}benzenesulfonamide* ***2e*** *34*

Figure S17 *N-{4-[({4-[(E)-2-phenylvinyl]phenyl}amino)sulfonyl]phenyl}acetamide* ***2f*** *36*

Figure S18 *2,4-dimethoxy-N-{4-[(E)-2-phenylvinyl]phenyl}benzenesulfonamide* ***2g*** *38*

Figure S19 *N-{4-[(E)-2-phenylvinyl]phenyl}methanesulfonamide* ***2h*** *40*

Figure S20 *N-{4-[(E)-2-phenylvinyl]phenyl}ethanesulfonamide* ***2i*** *42*

Figure S21 *1-phenyl-N-{4-[(E)-2-phenylvinyl]phenyl}methanesulfonamide* **2j** *44*

Figure S22 *5-chloro-N-{4-[(E)-2-phenylvinyl]phenyl}thiophene-2-sulfonamide* **2k** *46*

Table S1. *48*

Figure S23: *Chromatograms of the active compounds* ***1b-c*** *and* ***1j*** *in neutral conditions 49*

Figure S1: ^{1}H and ^{13}C NMR spectrum (300 MHz, C*D*Cl3) of *4-[(E)-2-phenylvinyl]phenylbenzenesulfonate* **1a**

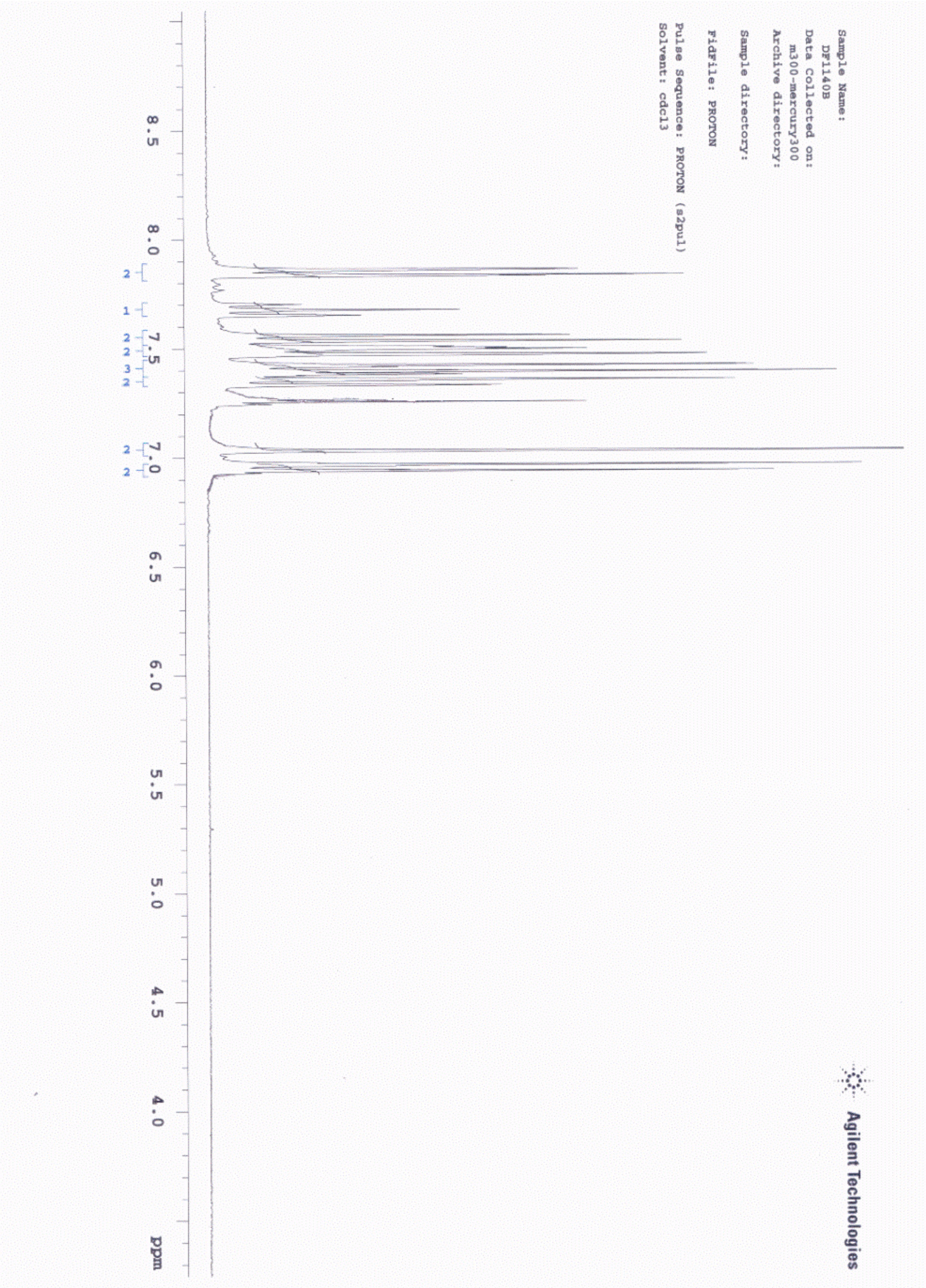

Sample Name:
DF1140B
Data Collected on:
m300-mercury300
Archive directory:

Sample directory:

FidFile: CARBON

Pulse Sequence: CARBON (s2pul)
Solvent: cdcl3
Data collected on: Mar 11 2021

Operator: defil

Relax. delay 1.000 sec
Pulse 45.0 degrees
Acq. time 0.868 sec
Width 18867.9 Hz
1826 repetitions
OBSERVE C13, 75.4847602 MHz
DECOUPLE H1, 300.1991980 MHz
Power 38 dB
continuously on
WALTZ-16 modulated
DATA PROCESSING
Line broadening 0.5 Hz
FT size 32768
Total time 1 hr, 4 min

Agilent Technologies

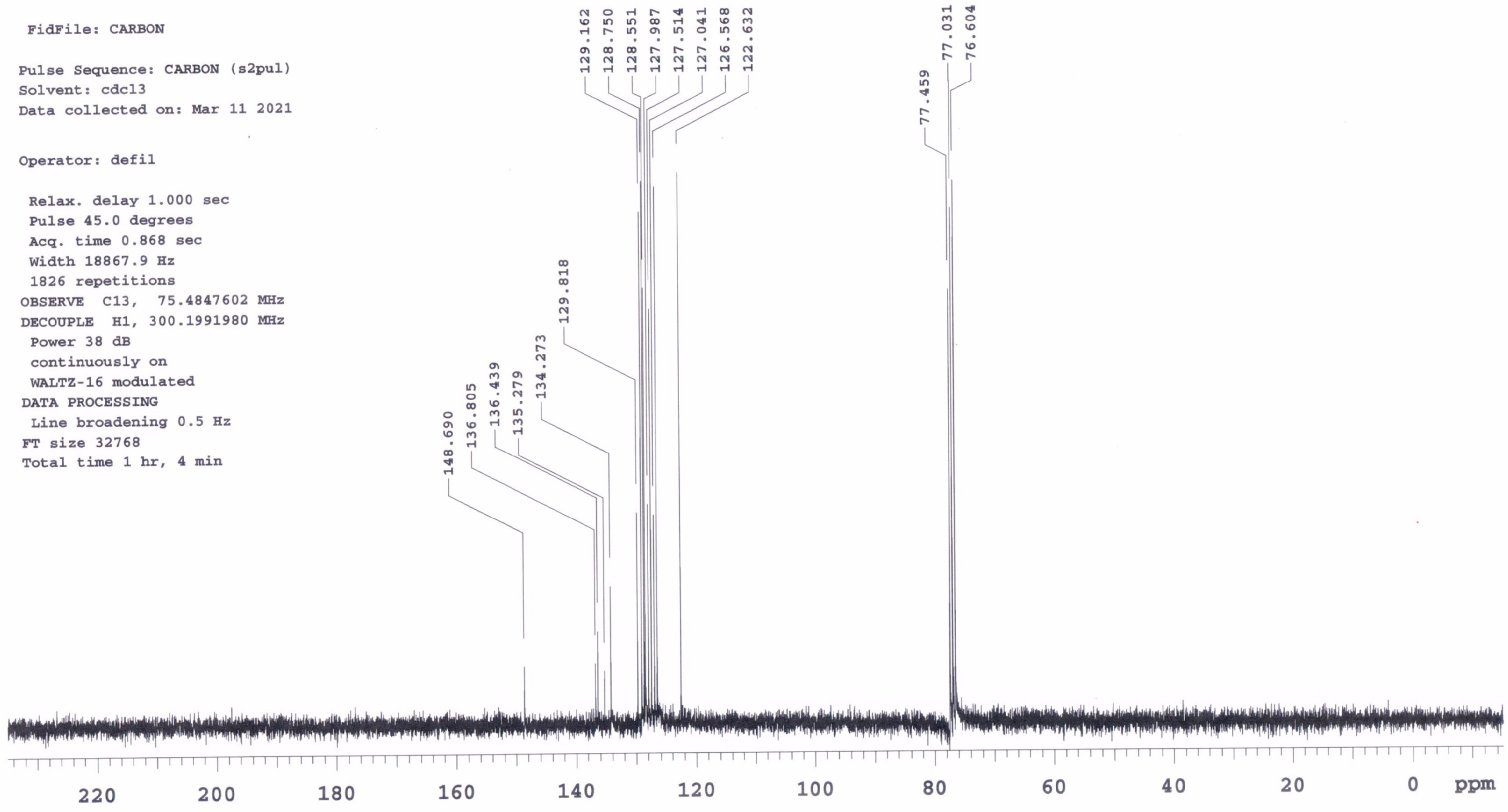

Figure S2: ^{1}H and ^{13}C NMR spectrum (300 MHz, $CDCl_3$) of 4-[(E)-2-phenylvinyl]phenyl 3-nitrobenzenesulfonate **1b**

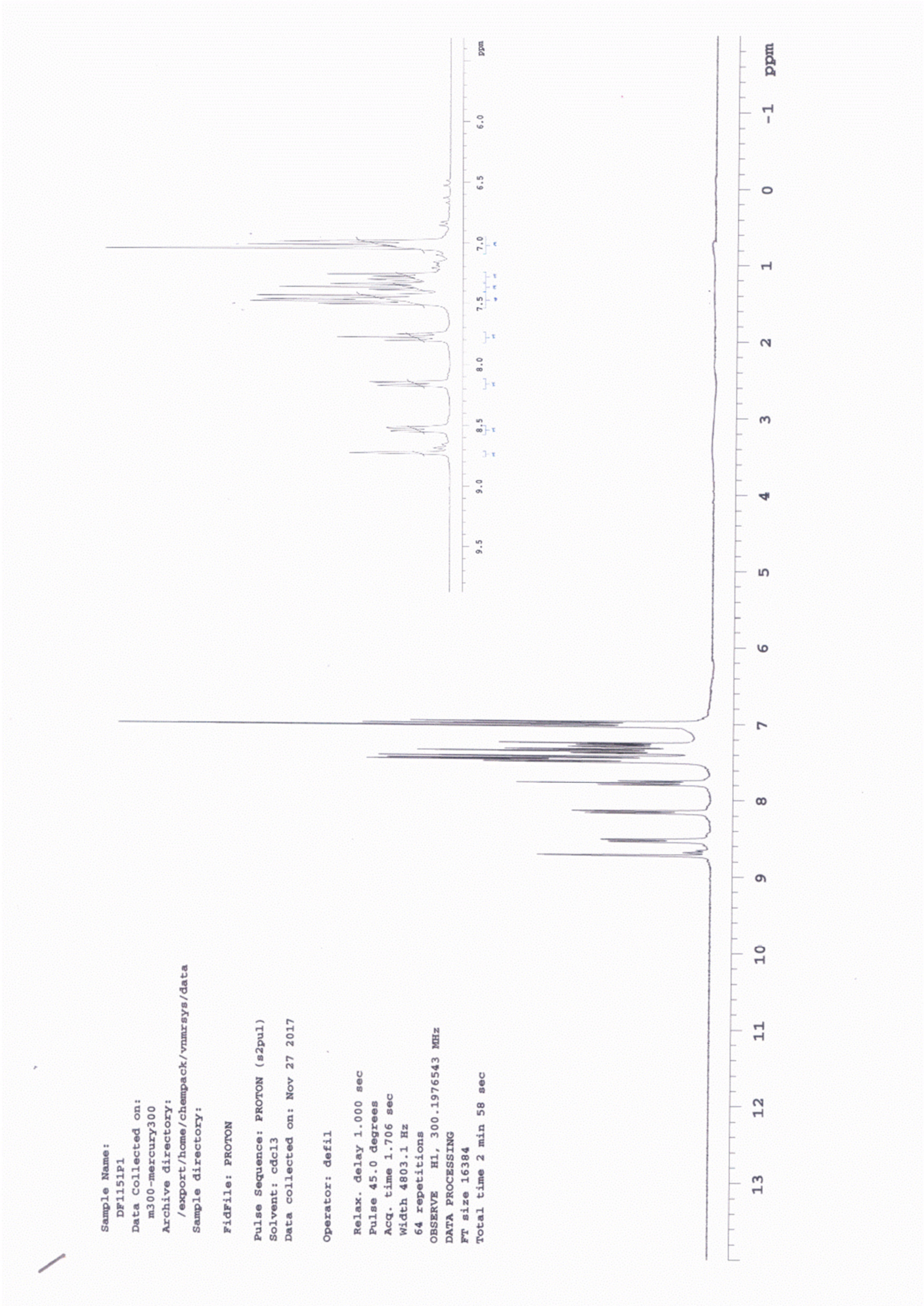

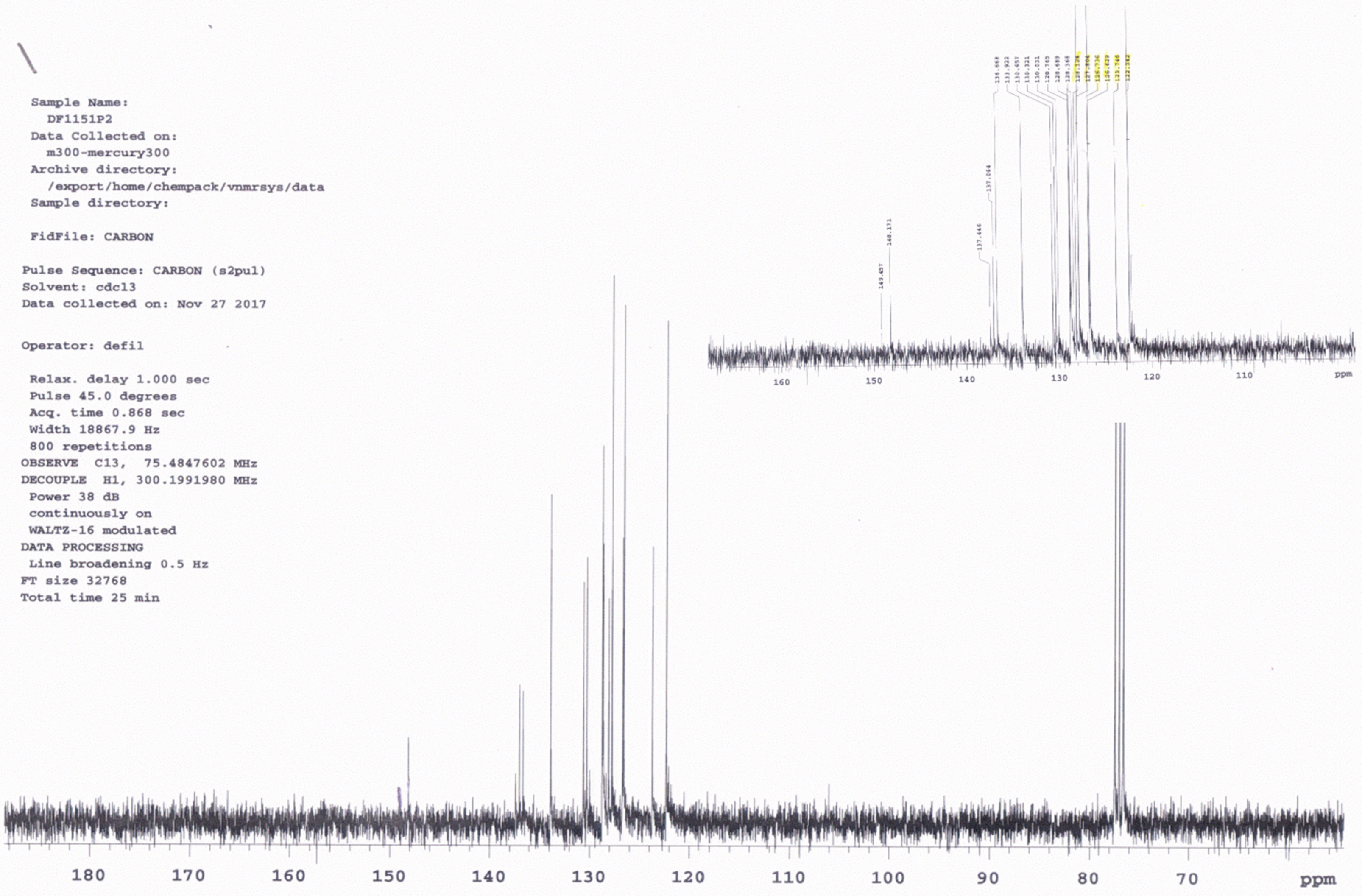
Sample Name:
DF1151P2
Data Collected on:
m300-mercury300
Archive directory:
/export/home/chempack/vnmrsys/data
Sample directory:
FidFile: CARBON
Pulse Sequence: CARBON (s2pul)
Solvent: cdcl3
Data collected on: Nov 27 2017
Operator: defil
Relax. delay 1.000 sec
Pulse 45.0 degrees
Acq. time 0.868 sec
Width 18867.9 Hz
800 repetitions
OBSERVE C13, 75.4847602 MHz
DECOUPLE H1, 300.1991980 MHz
Power 38 dB
continuously on
WALTZ-16 modulated
DATA PROCESSING
Line broadening 0.5 Hz
FT size 32768
Total time 25 min
149.457
148.171
137.446
137.044
136.668
133.922
130.657
130.321
130.031
128.765
128.689
128.368
128.126
127.804
126.736
126.629
123.746
122.382
160
150
140
130
120
110
ppm
180
170
160
150
140
130
120
110
100
90
80
70
ppm

Figure S3: ^{1}H NMR (300 MHz, $CDCl_3$) and ^{13}C spectrum (300 MHz, $CDCl_3$) of *4-[(E)-2-phenylvinyl]phenyl 4-methyl-2-nitrobenzenesulfonate* **1c**

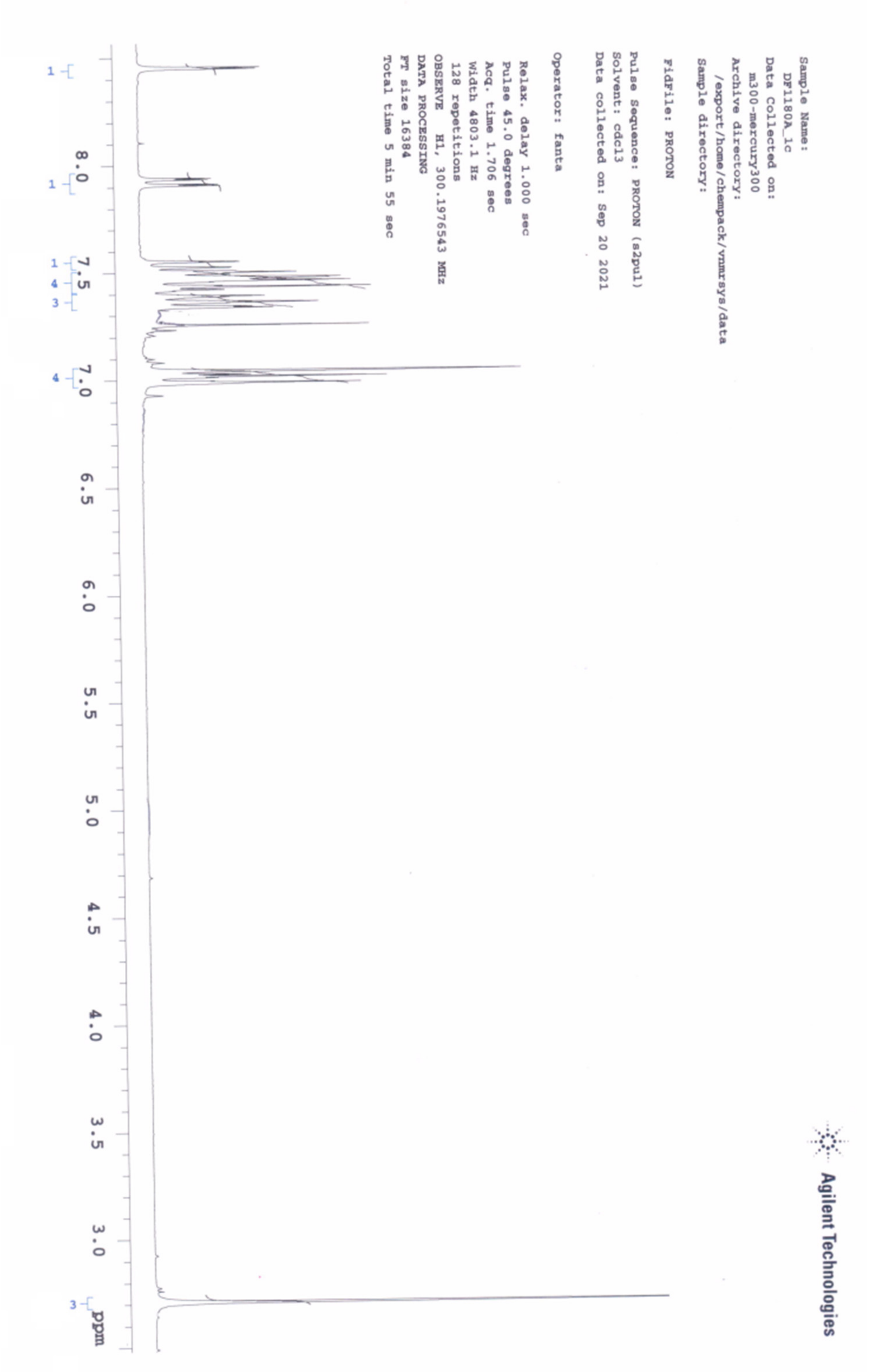

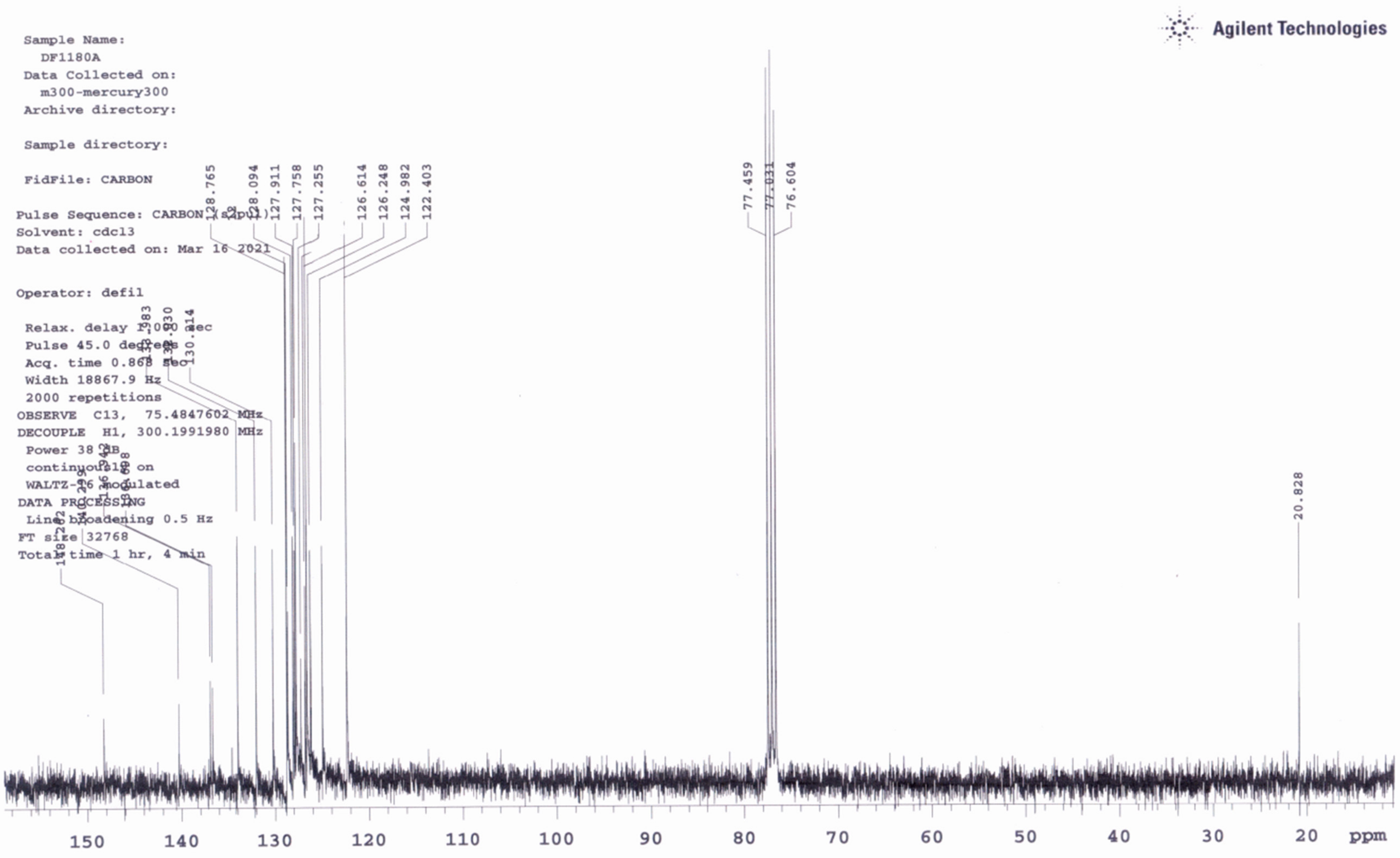

Sample Name:
DF1180A
Data Collected on:
m300-mercury300
Archive directory:
Sample directory:
FidFile: CARBON
Pulse Sequence: CARBON (s2pul)
Solvent: cdcl3
Data collected on: Mar 16 2021
Operator: defil
Relax. delay 1.000 sec
Pulse 45.0 degrees
Acq. time 0.868 sec
Width 18867.9 Hz
2000 repetitions
OBSERVE C13, 75.4847602 MHz
DECOUPLE H1, 300.1991980 MHz
Power 38 dB
continuously on
WALTZ-16 modulated
DATA PROCESSING
Line broadening 0.5 Hz
FT size 32768
Total time 1 hr, 4 min
Agilent Technologies
130.214
128.765
128.094
127.911
127.758
127.255
126.614
126.248
124.982
122.403
77.459
77.031
76.604
20.828
150
140
130
120
110
100
90
80
70
60
50
40
30
20
ppm

Figure S4: ^{1}H NMR and ^{13}C spectrum (300 MHz, CDCl$_3$) of *4-[(E)-2-phenylvinyl]phenyl 4-methylbenzenesulfonate* ***1d***

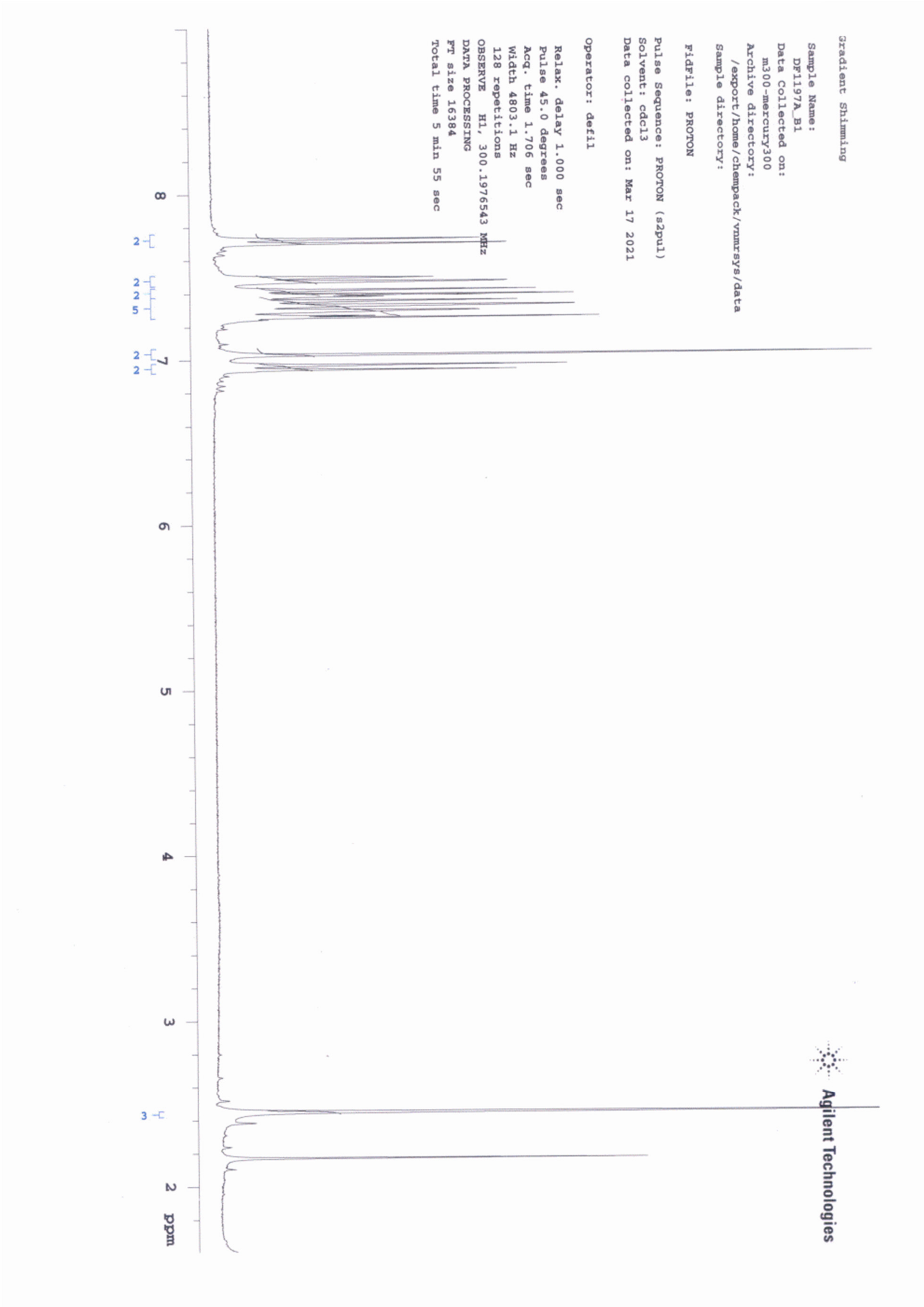

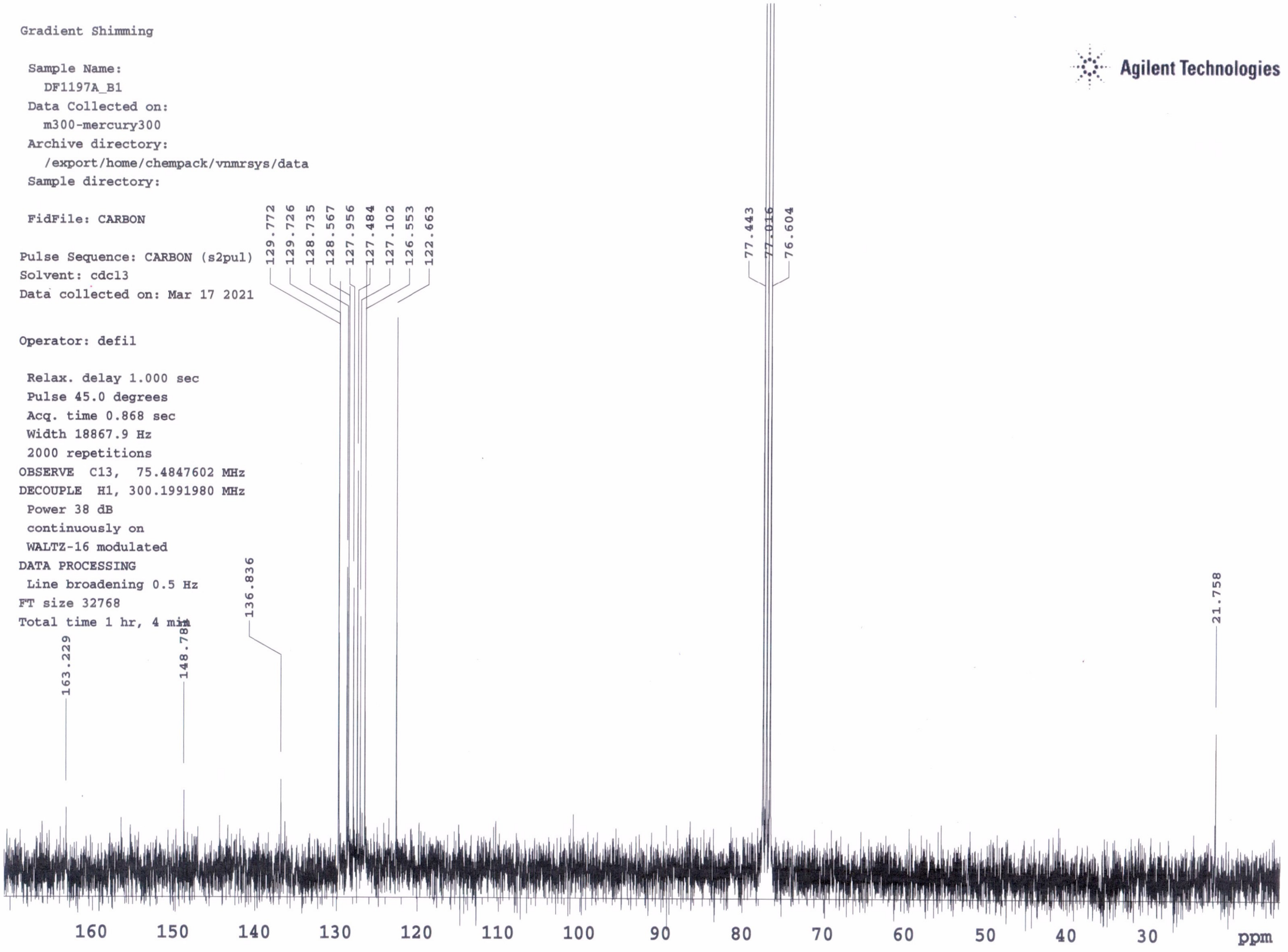

Gradient Shimming
Sample Name:
DF1197A_B1
Data Collected on:
m300-mercury300
Archive directory:
/export/home/chempack/vnmrsys/data
Sample directory:
FidFile: CARBON
Pulse Sequence: CARBON (s2pul)
Solvent: cdcl3
Data collected on: Mar 17 2021
Operator: defil
Relax. delay 1.000 sec
Pulse 45.0 degrees
Acq. time 0.868 sec
Width 18867.9 Hz
2000 repetitions
OBSERVE C13, 75.4847602 MHz
DECOUPLE H1, 300.1991980 MHz
Power 38 dB
continuously on
WALTZ-16 modulated
DATA PROCESSING
Line broadening 0.5 Hz
FT size 32768
Total time 1 hr, 4 min
Agilent Technologies
163.229
148.78
136.836
129.772
129.726
128.735
128.567
127.956
127.484
127.102
126.553
122.663
77.443
77.016
76.604
21.758
160
150
140
130
120
110
100
90
80
70
60
50
40
30
ppm

Figure S5*:* ^{1}H NMR and ^{13}C spectrum (300 MHz, C*D*Cl$_3$) of *4-[(E)-2-phenylvinyl]phenyl 4-cyanobenzenesulfonate* ***1e***

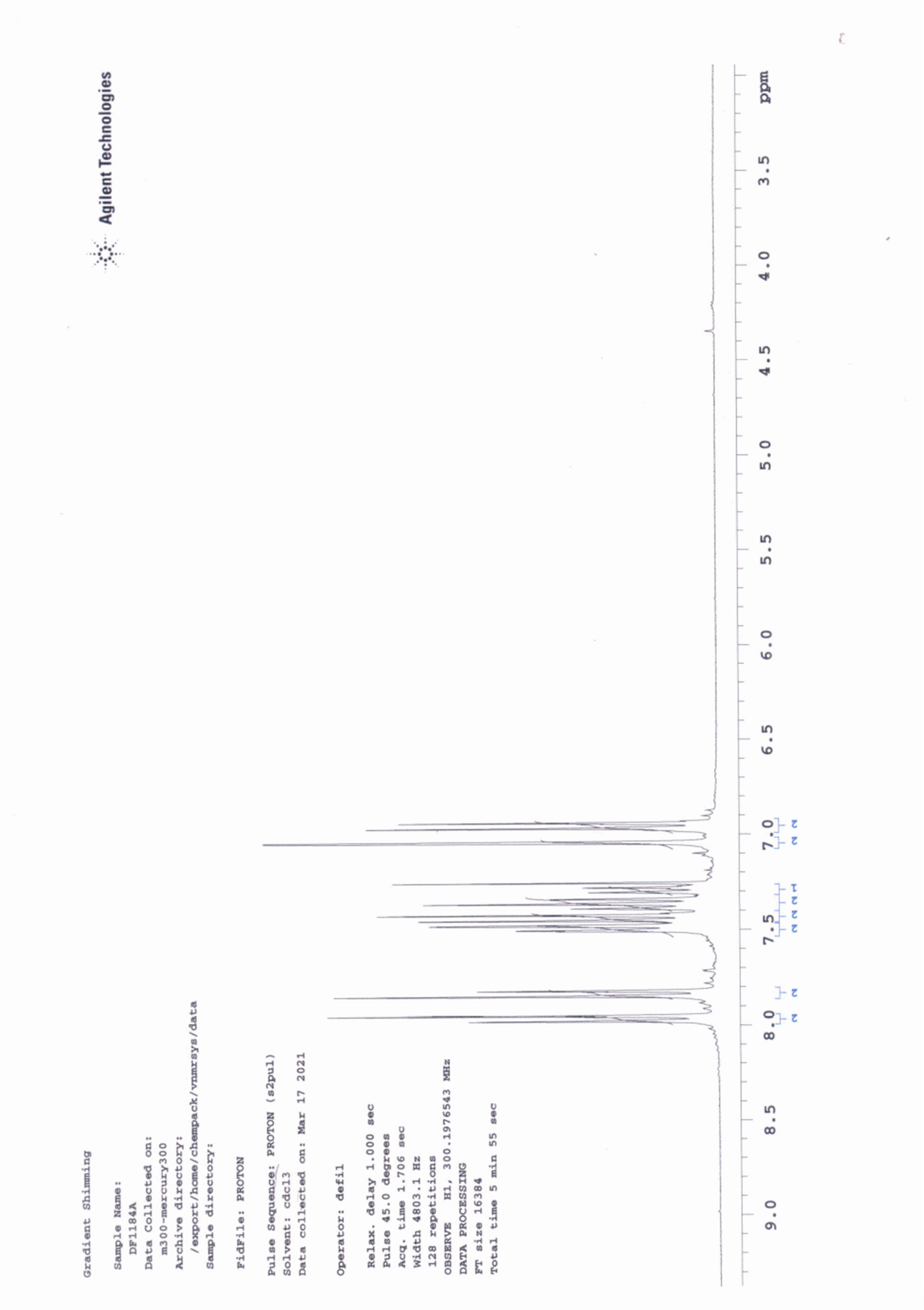

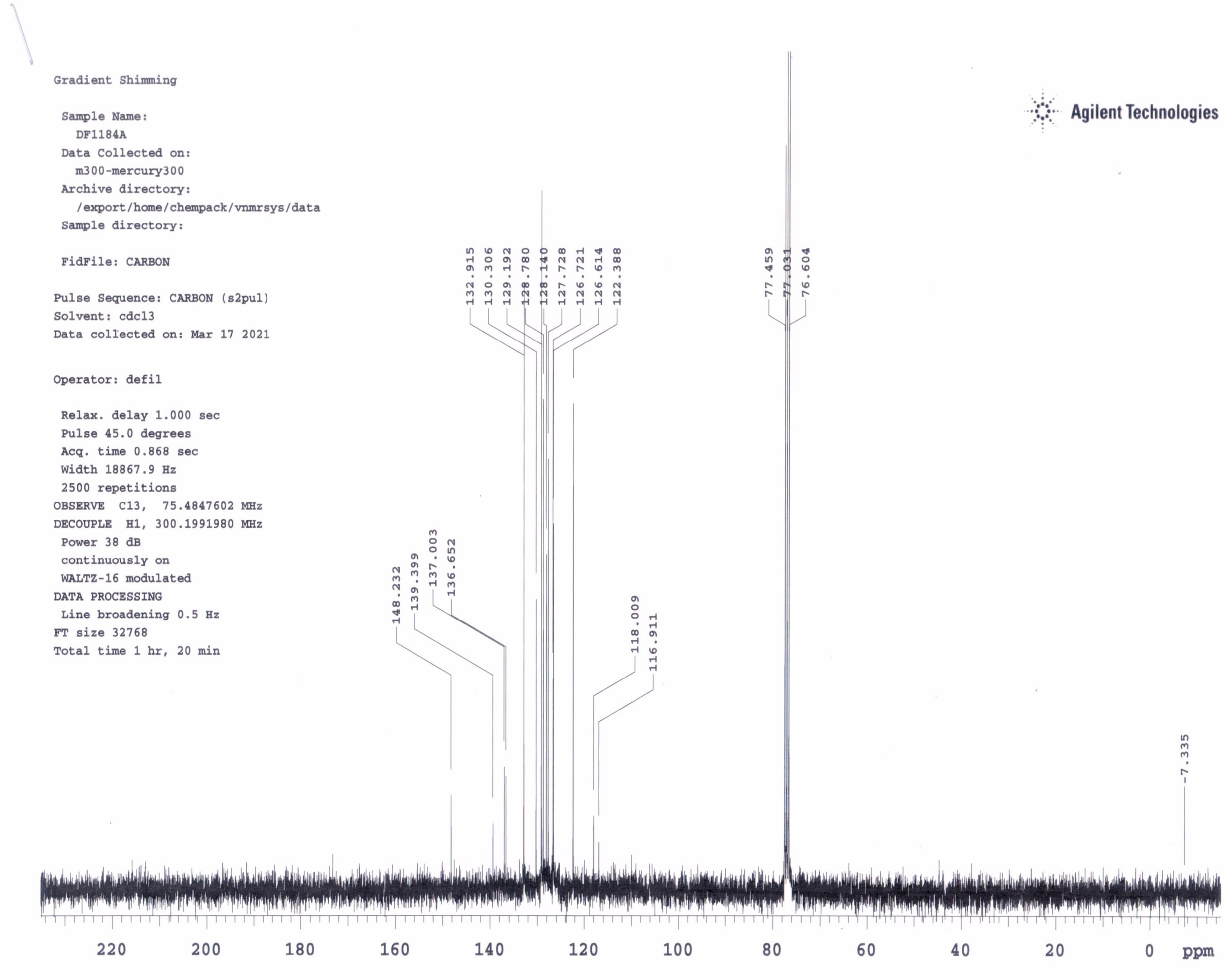

Gradient Shimming
Sample Name:
DF1184A
Data Collected on:
m300-mercury300
Archive directory:
/export/home/chempack/vnmrsys/data
Sample directory:
FidFile: CARBON
Pulse Sequence: CARBON (s2pul)
Solvent: cdcl3
Data collected on: Mar 17 2021
Operator: defil
Relax. delay 1.000 sec
Pulse 45.0 degrees
Acq. time 0.868 sec
Width 18867.9 Hz
2500 repetitions
OBSERVE C13, 75.4847602 MHz
DECOUPLE H1, 300.1991980 MHz
Power 38 dB
continuously on
WALTZ-16 modulated
DATA PROCESSING
Line broadening 0.5 Hz
FT size 32768
Total time 1 hr, 20 min
Agilent Technologies
148.232
139.399
137.003
136.652
132.915
130.306
129.192
128.780
128.140
127.728
126.721
126.614
122.388
118.009
116.911
77.459
77.031
76.604
-7.335
220
200
180
160
140
120
100
80
60
40
20
0
ppm

Figure S6: ^{1}H NMR and ^{13}C spectrum (300 MHz, Acetone-*d*) of *4-[(E)-2-phenylvinyl]phenyl 4-(acetylamino)benzenesulfonate* ***1f***

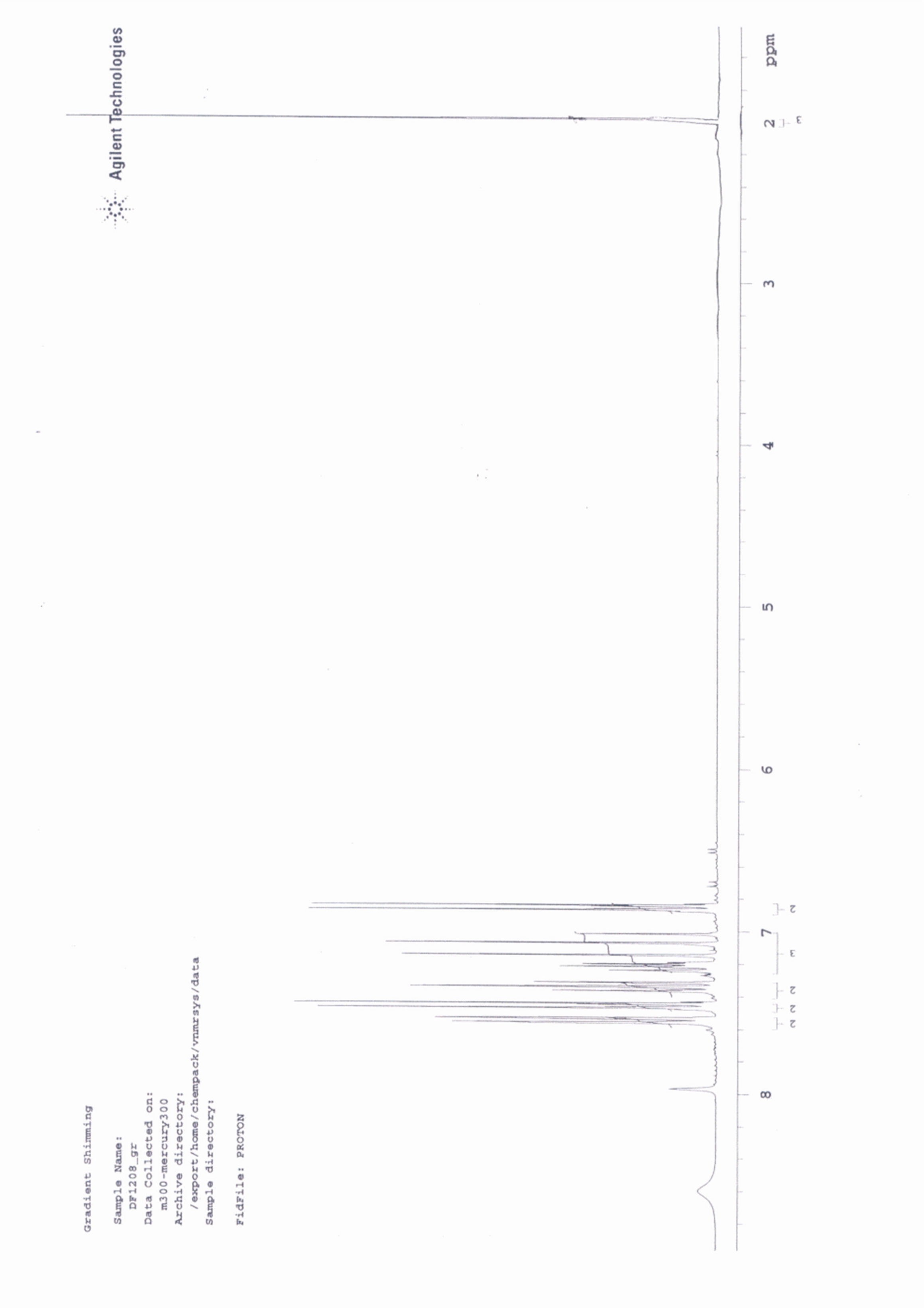

Gradient Shimming

Sample Name:

Data Collected on:
m300-mercury300
Archive directory:
/export/home/chempack/vnmrsys/data
Sample directory:

FidFile: CARBON

Pulse Sequence: CARBON (s2pul)
Solvent:
Data collected on: Jul 11 2018

Operator: defil

Relax. delay 1.000 sec
Pulse 45.0 degrees
Acq. time 0.868 sec
Width 18867.9 Hz
1200 repetitions
OBSERVE C13, 75.4847602 MHz
DECOUPLE H1, 300.1991980 MHz
Power 38 dB
continuously on
WALTZ-16 modulated
DATA PROCESSING
Line broadening 0.5 Hz
FT size 32768
Total time 38 min

Agilent Technologies

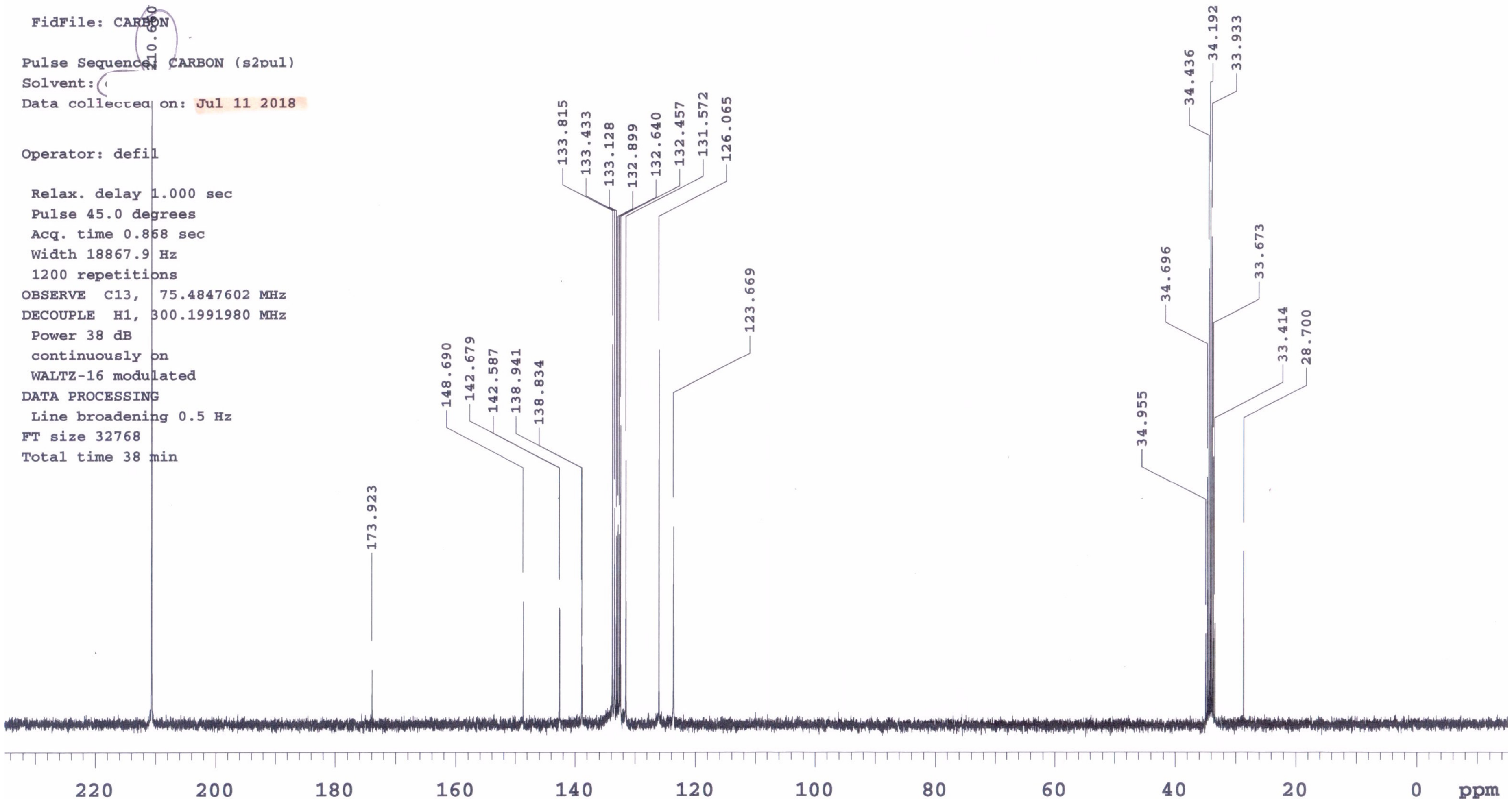

Figure S7*:* ^{1}H NMR and ^{13}C spectrum (300 MHz, C*D*Cl3) of *4-[(E)-2-phenylvinyl]phenyl 2,4-dimethoxybenzenesulfonate* ***1g***

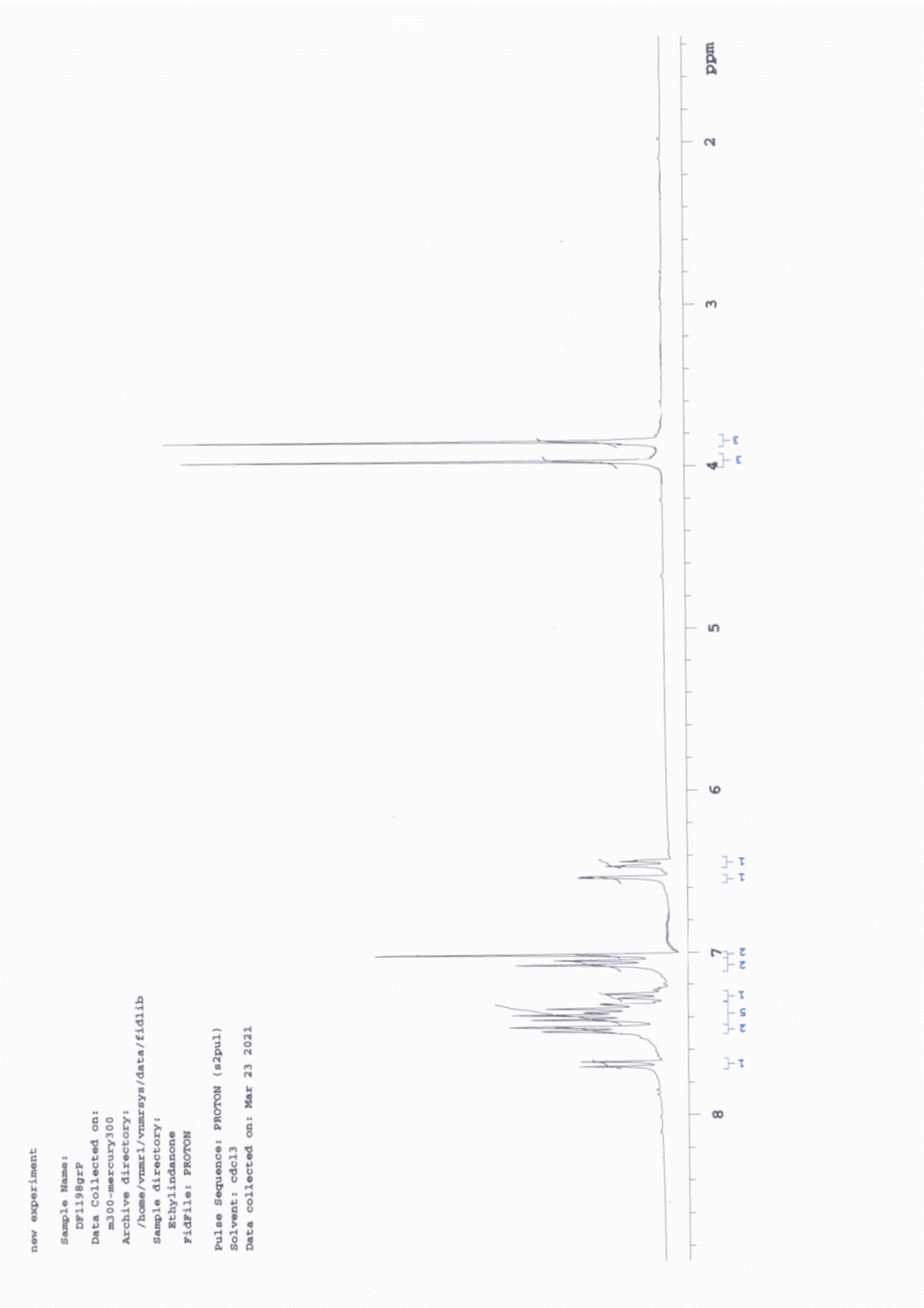

Gradient Shimming

Sample Name:
DF1198grezzo
Data Collected on:
m300-mercury300
Archive directory:
/export/home/chempack/vnmrsys/data
Sample directory:

FidFile: CARBON

Pulse Sequence: CARBON (s2pul)
Solvent: cdcl3
Data collected on: May 3 2018

Operator: defil

Relax. delay 1.000 sec
Pulse 45.0 degrees
Acq. time 0.868 sec
Width 18867.9 Hz
797 repetitions
OBSERVE C13, 75.4847602 MHz
DECOUPLE H1, 300.1991980 MHz
Power 38 dB
continuously on
WALTZ-16 modulated
DATA PROCESSING
Line broadening 0.5 Hz
FT size 32768
Total time 25 min

Agilent Technologies

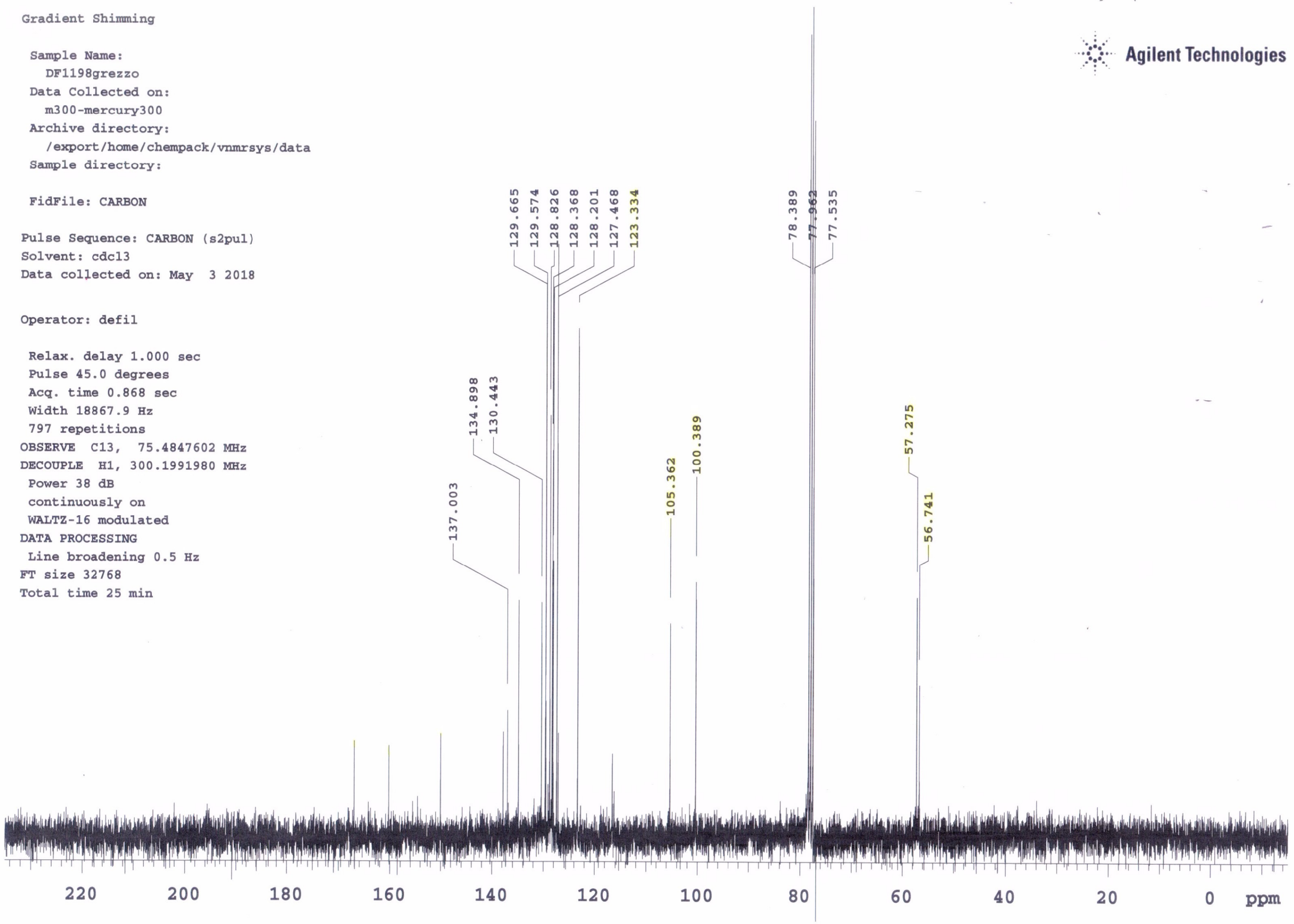

Figure S8: ^{1}H NMR and ^{13}C spectrum (300 MHz, C*D*Cl$_3$) of *4-[(E)-2-phenylvinyl]phenylmethanesulfonate* ***1h***

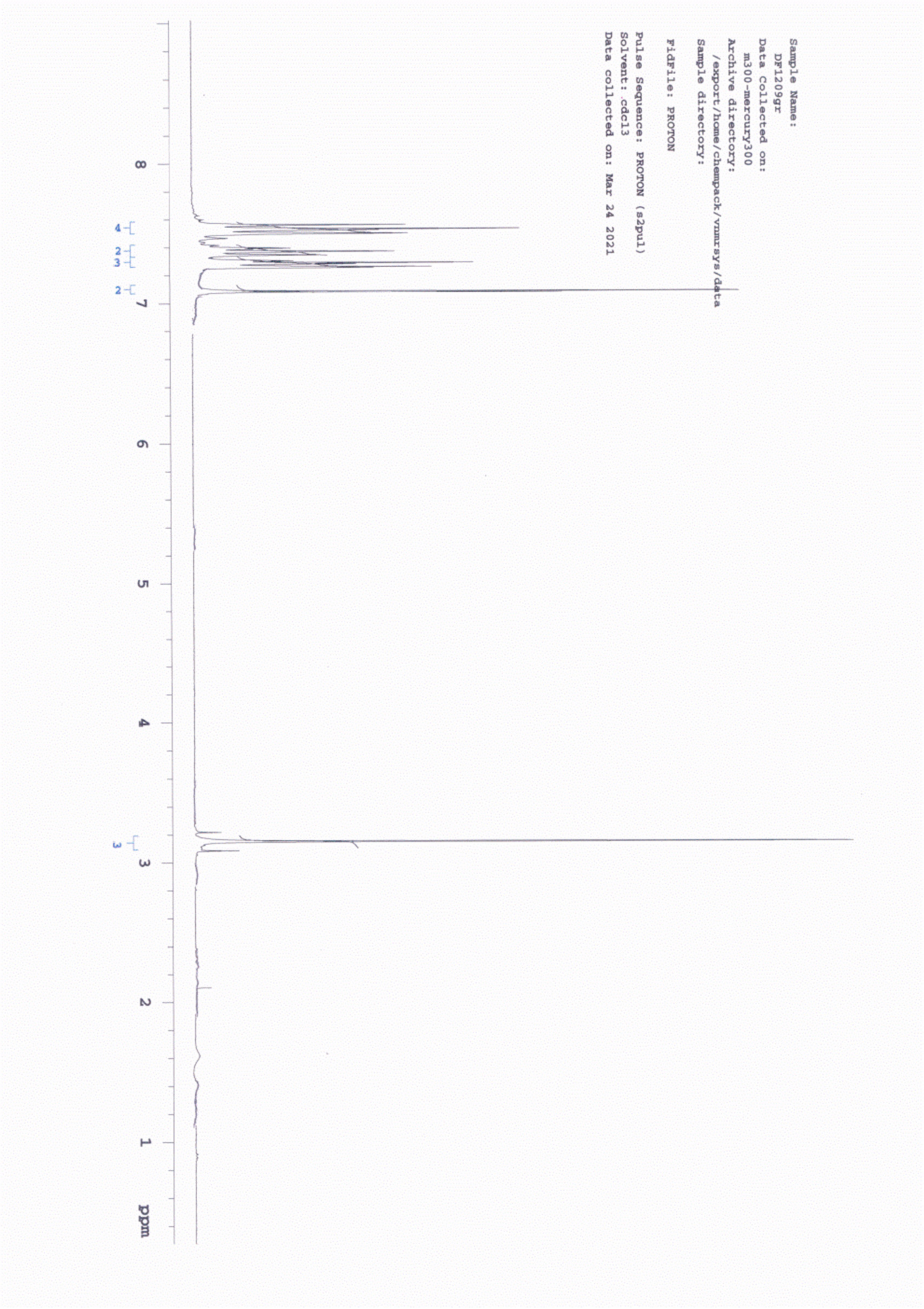

Agilent Technologies

Sample Name:
DF1209gr
Data Collected on:
m300-mercury300
Archive directory:
/export/home/chempack/vnmrsys/data
Sample directory:

FidFile: CARBON

Pulse Sequence: CARBON (s2pul)
Solvent: cdcl3
Data collected on: Mar 24 2021

Operator: fanta

Relax. delay 1.000 sec
Pulse 45.0 degrees
Acq. time 0.868 sec
Width 18867.9 Hz
2000 repetitions
OBSERVE C13, 75.4847602 MHz
DECOUPLE H1, 300.1991980 MHz
Power 38 dB
continuously on
WALTZ-16 modulated
DATA PROCESSING
Line broadening 0.5 Hz
FT size 32768
Total time 1 hr, 4 min

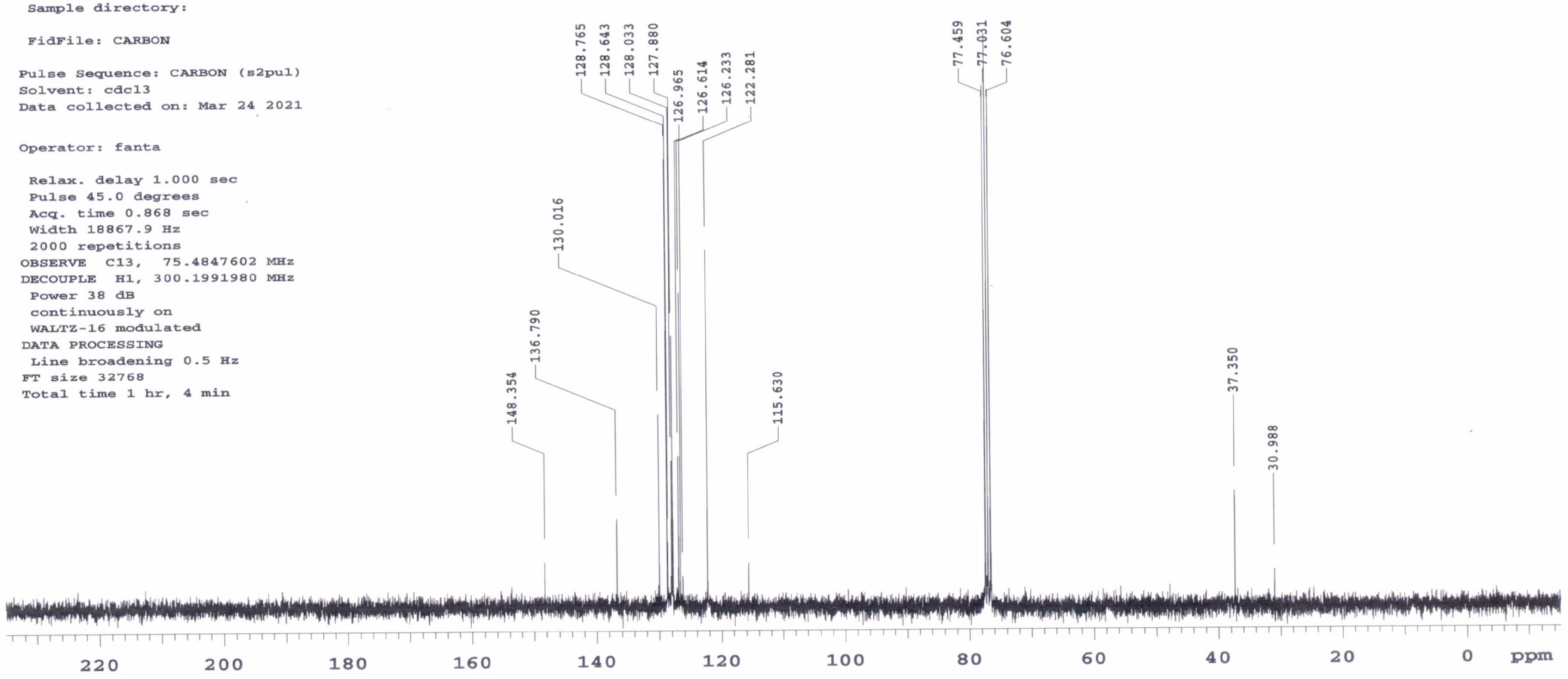

Figure S9: ^{1}H NMR and ^{13}C spectrum (300 MHz, $CDCl_3$) of *4-[(E)-2-phenylvinyl]phenyl ethanesulfonate* **1i**

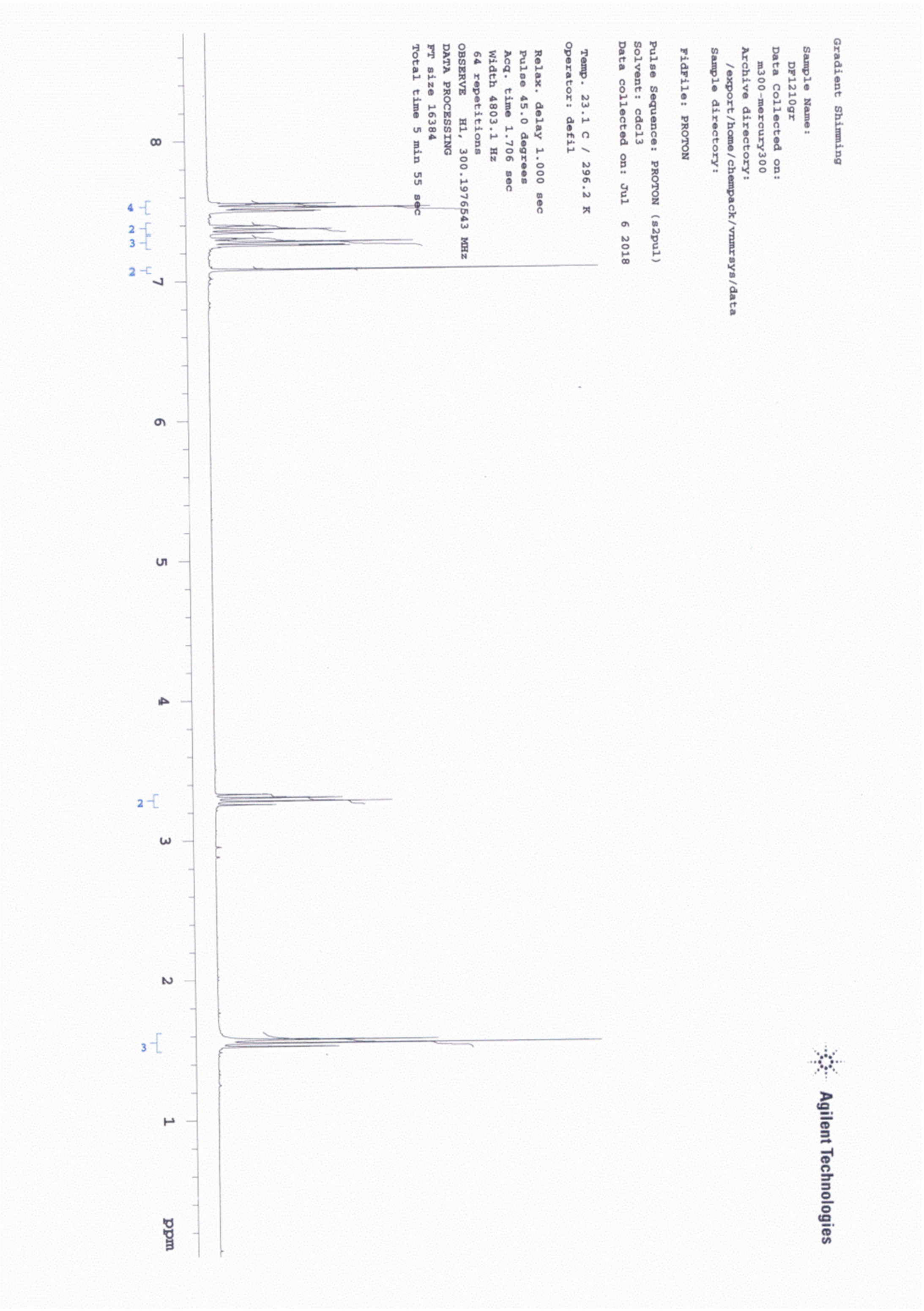

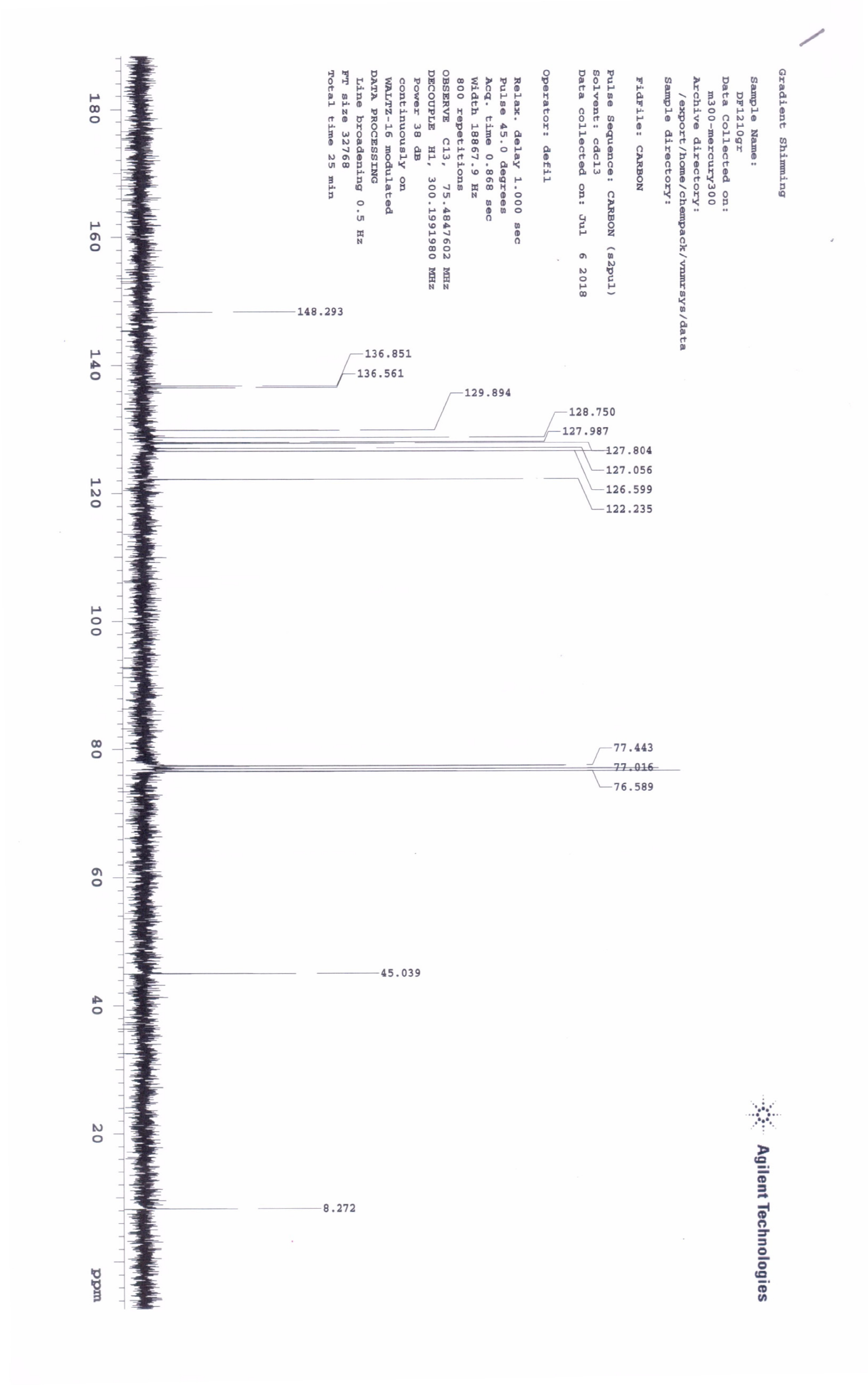
Gradient Shimming
Sample Name:
DF1210gr
Data Collected on:
m300-mercury300
Archive directory:
/export/home/chempack/vnmrsys/data
Sample directory:
FidFile: CARBON
Pulse Sequence: CARBON (s2pul)
Solvent: cdcl3
Data collected on: Jul 6 2018
Operator: defil
Relax. delay 1.000 sec
Pulse 45.0 degrees
Acq. time 0.868 sec
Width 18867.9 Hz
800 repetitions
OBSERVE C13, 75.4847602 MHz
DECOUPLE H1, 300.1991980 MHz
Power 38 dB
continuously on
WALTZ-16 modulated
DATA PROCESSING
Line broadening 0.5 Hz
FT size 32768
Total time 25 min
148.293
136.851
136.561
129.894
128.750
127.987
127.804
127.056
126.599
122.235
77.443
77.016
76.589
45.039
8.272
180
160
140
120
100
80
60
40
20
ppm
Agilent Technologies

Figure S10: [1]NMR and ^{13}C spectrum (300 MHz, CDCl3) of *4-[(E)-2-phenylvinyl]phenyl phenylmethanesulfonate* **1j**

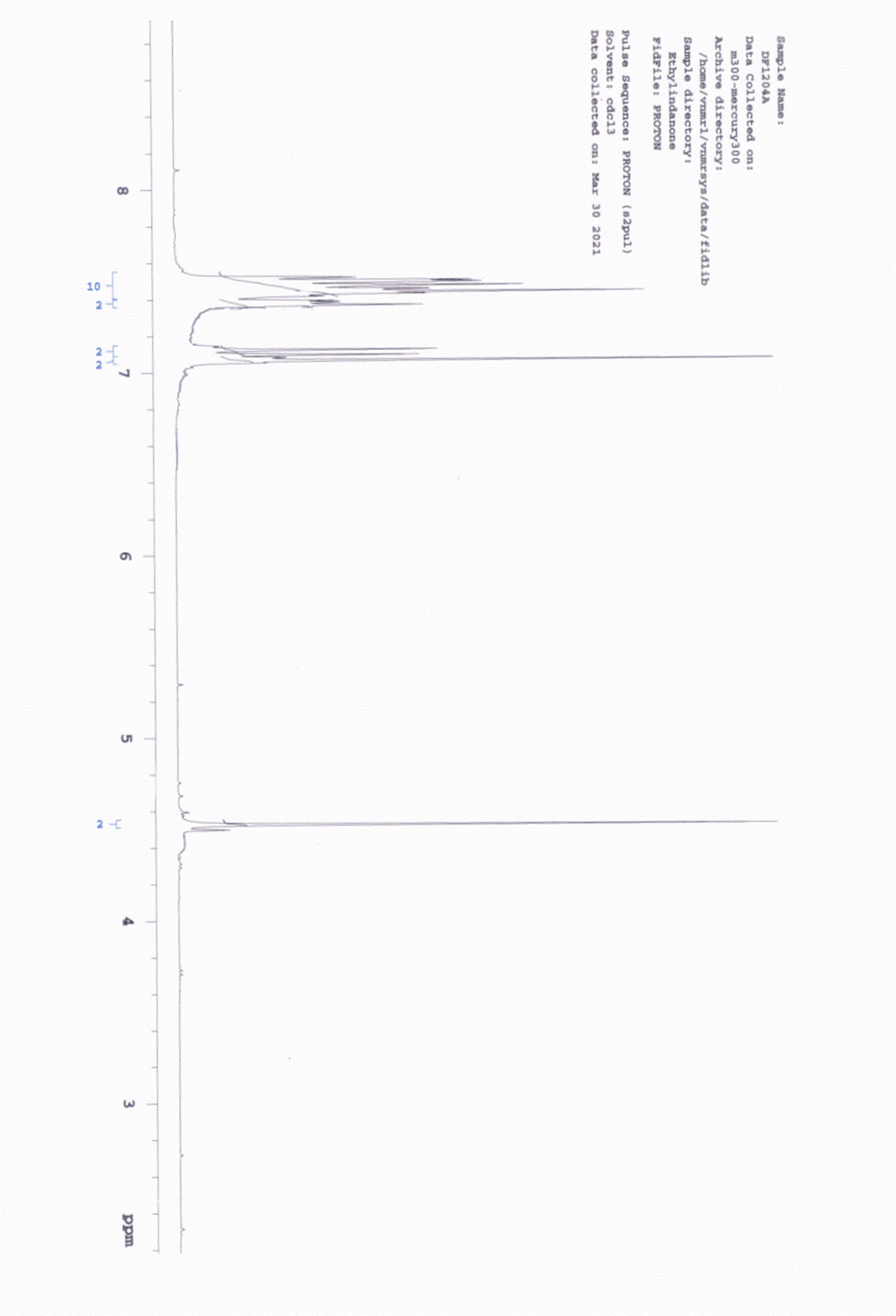

new experiment

Sample Name:
DF1204A
Data Collected on:
m300-mercury300
Archive directory:
/home/vnmr1/vnmrsys/data/fidlib
Sample directory:
Ethylindanone
FidFile: CARBON

Pulse Sequence: CARBON (s2pul)
Solvent: cdcl3
Data collected on: Mar 30 2021

Operator: defil

Relax. delay 1.000 sec
Pulse 45.0 degrees
Acq. time 0.868 sec
Width 18867.9 Hz
1344 repetitions
OBSERVE C13, 75.4847602 MHz
DECOUPLE H1, 300.1991980 MHz
Power 38 dB
continuously on
WALTZ-16 modulated
DATA PROCESSING
Line broadening 0.5 Hz
FT size 32768
Total time 1 hr, 4 min

Agilent Technologies

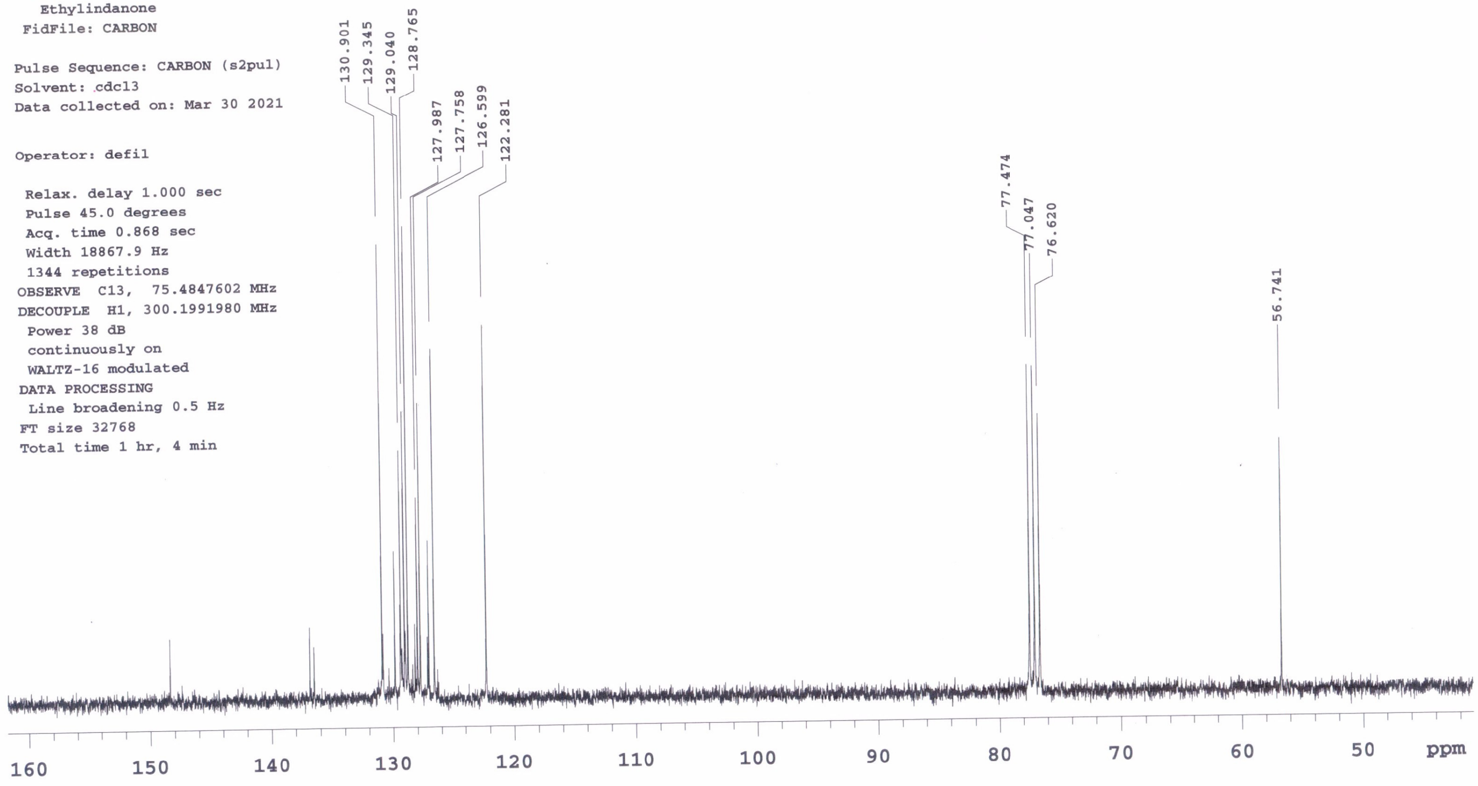

Figure S11: ^{1}H NMR and ^{13}C spectrum (300 MHz, $CDCl_3$) of *4-[(E)-2-phenylvinyl]phenyl 5-chlorothiophene-2-sulfonate* **1k**

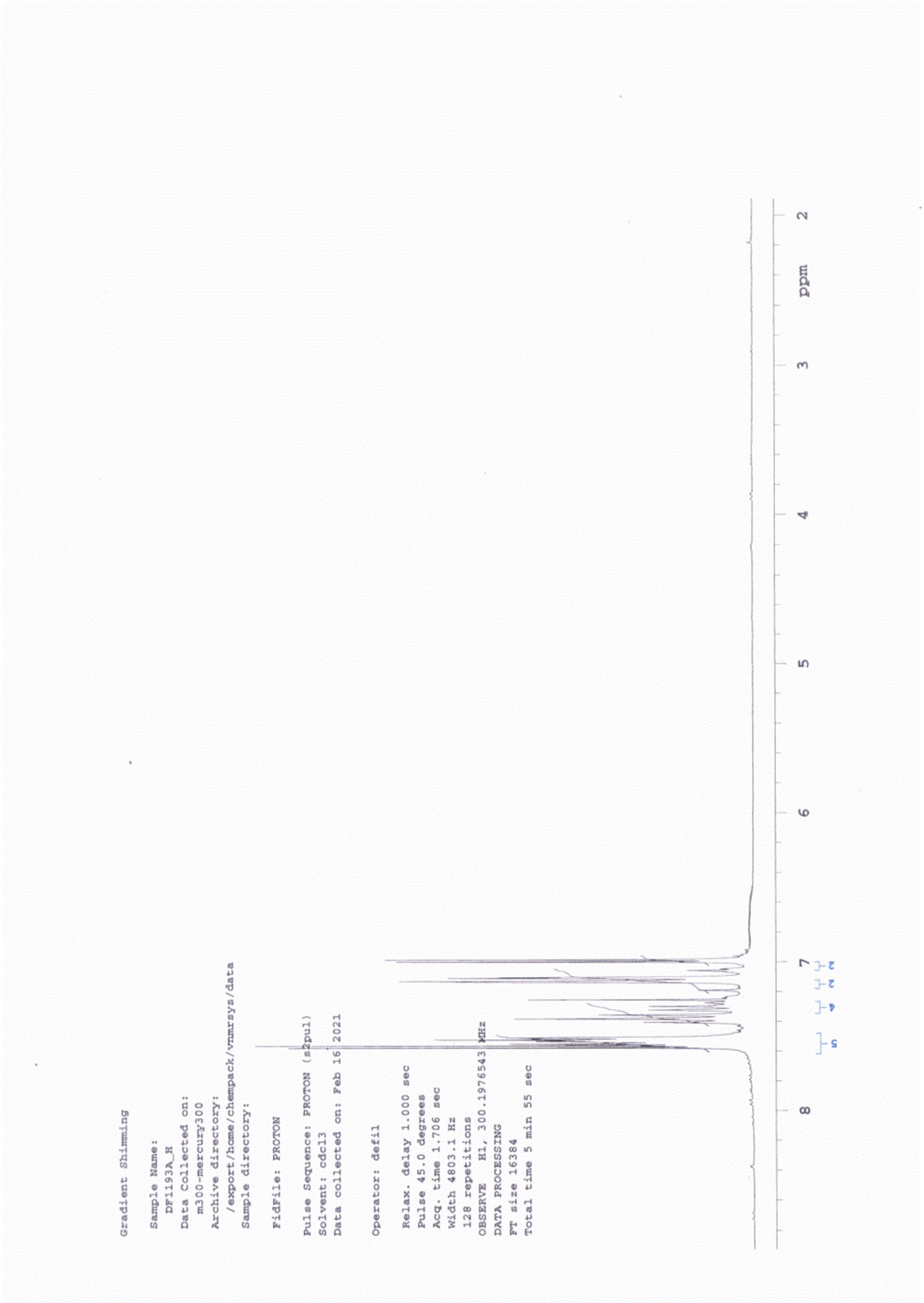

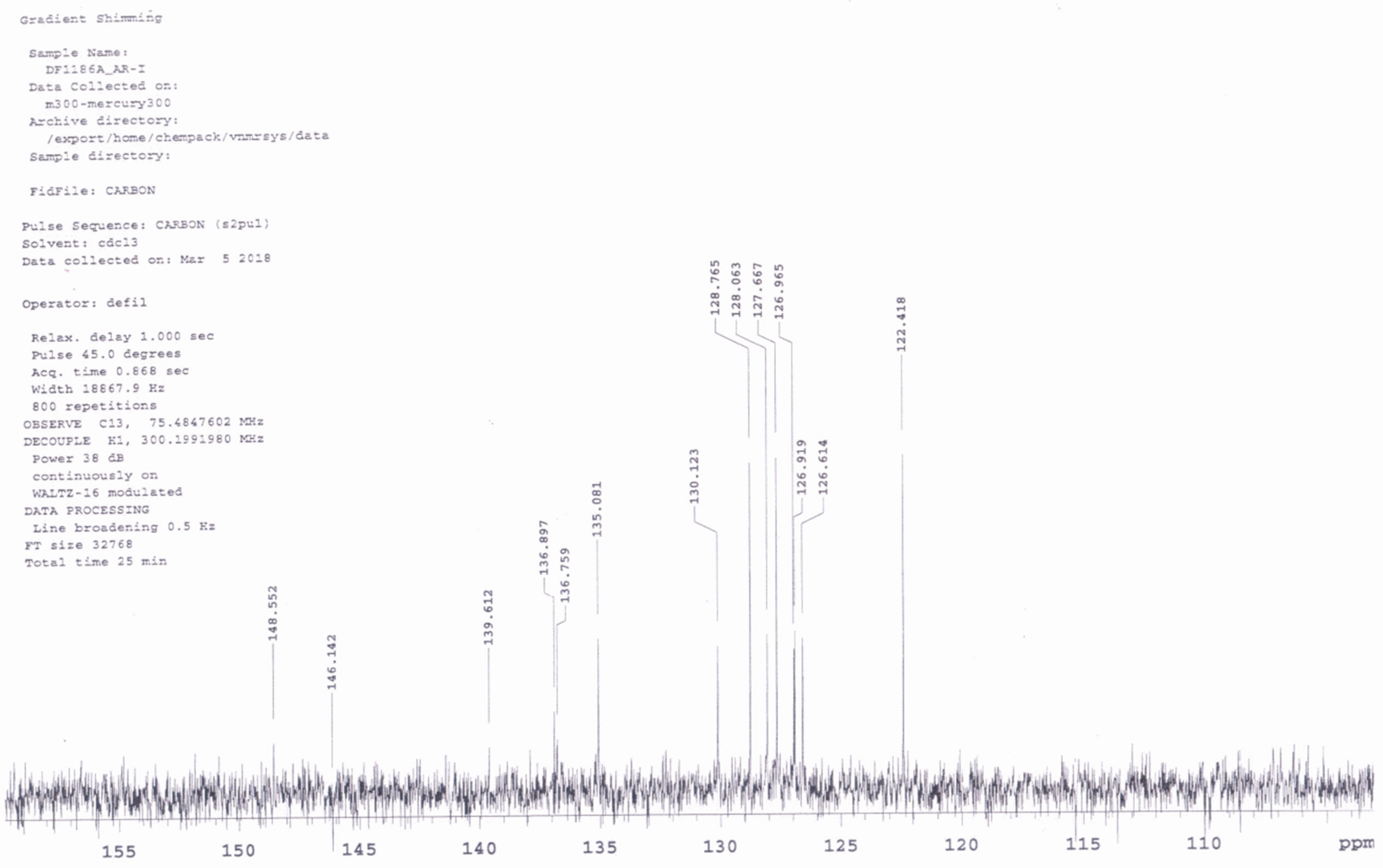

Gradient Shimming
Sample Name:
DF1186A_AR-I
Data Collected on:
m300-mercury300
Archive directory:
/export/home/chempack/vnmrsys/data
Sample directory:
FidFile: CARBON
Pulse Sequence: CARBON (s2pul)
Solvent: cdcl3
Data collected on: Mar 5 2018
Operator: defil
Relax. delay 1.000 sec
Pulse 45.0 degrees
Acq. time 0.868 sec
Width 18867.9 Hz
800 repetitions
OBSERVE C13, 75.4847602 MHz
DECOUPLE H1, 300.1991980 MHz
Power 38 dB
continuously on
WALTZ-16 modulated
DATA PROCESSING
Line broadening 0.5 Hz
FT size 32768
Total time 25 min
148.552
146.142
139.612
136.897
136.759
135.081
130.123
128.765
128.063
127.667
126.965
126.919
126.614
122.418
155
150
145
140
135
130
125
120
115
110
ppm

Figure S12: ^{1}H NMR and ^{13}C spectrum (300 MHz, C*D*Cl$_3$) of *N-{4-[(E)-2-phenylvinyl]phenyl} benzenesulfonamide* ***2a***

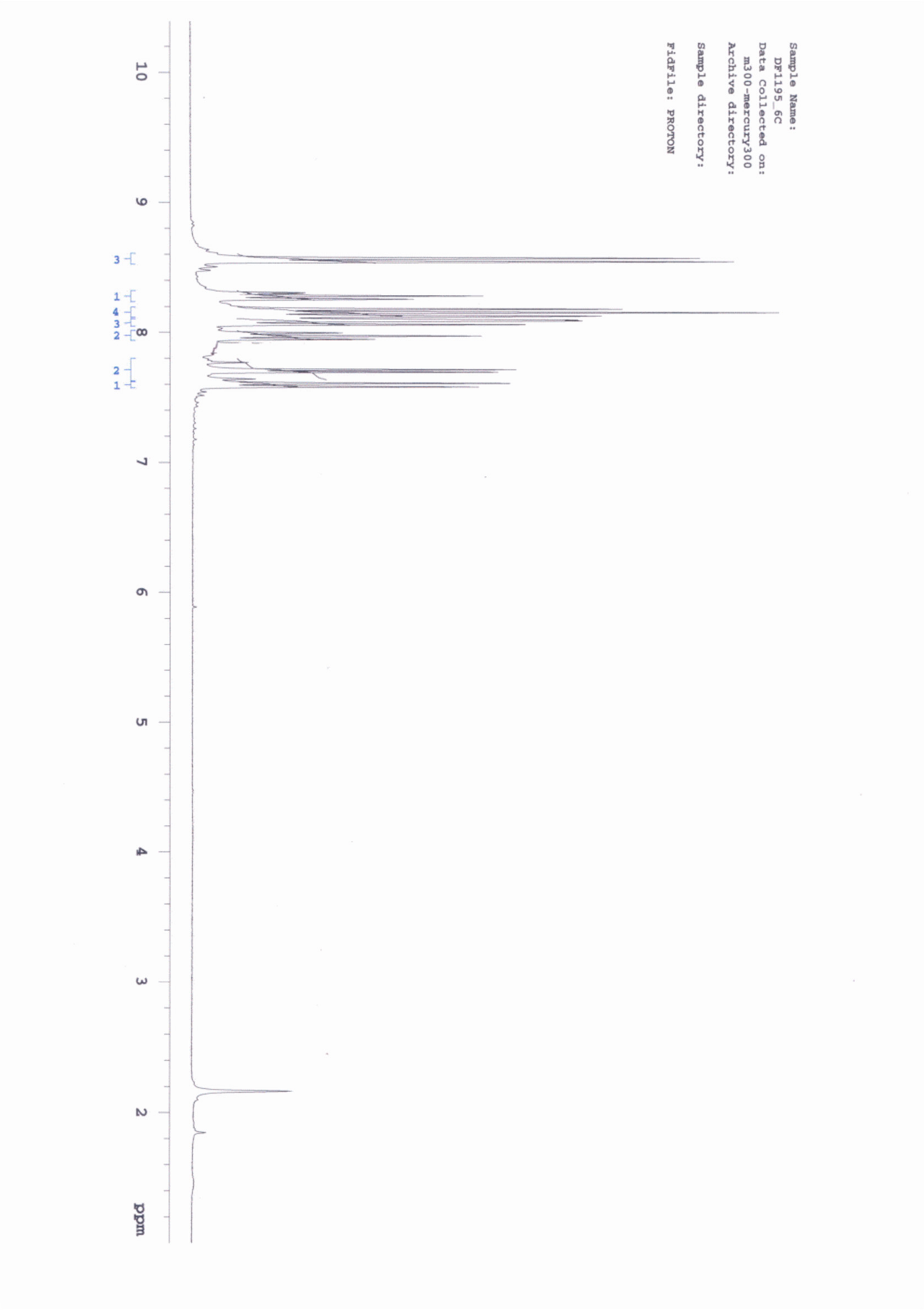

Agilent Technologies

Sample Name:
DF1195_6C
Data Collected on:
m300-mercury300
Archive directory:

Sample directory:

FidFile: CARBON

Pulse Sequence: CARBON (s2pul)
Solvent: cdcl3
Data collected on: Mar 10 2021

Operator: defil

Relax. delay 1.000 sec
Pulse 45.0 degrees
Acq. time 0.868 sec
Width 18867.9 Hz
2341 repetitions
OBSERVE C13, 75.4847602 MHz
DECOUPLE H1, 300.1991980 MHz
Power 38 dB
continuously on
WALTZ-16 modulated
DATA PROCESSING
Line broadening 0.5 Hz
FT size 32768
Total time 1 hr, 20 min

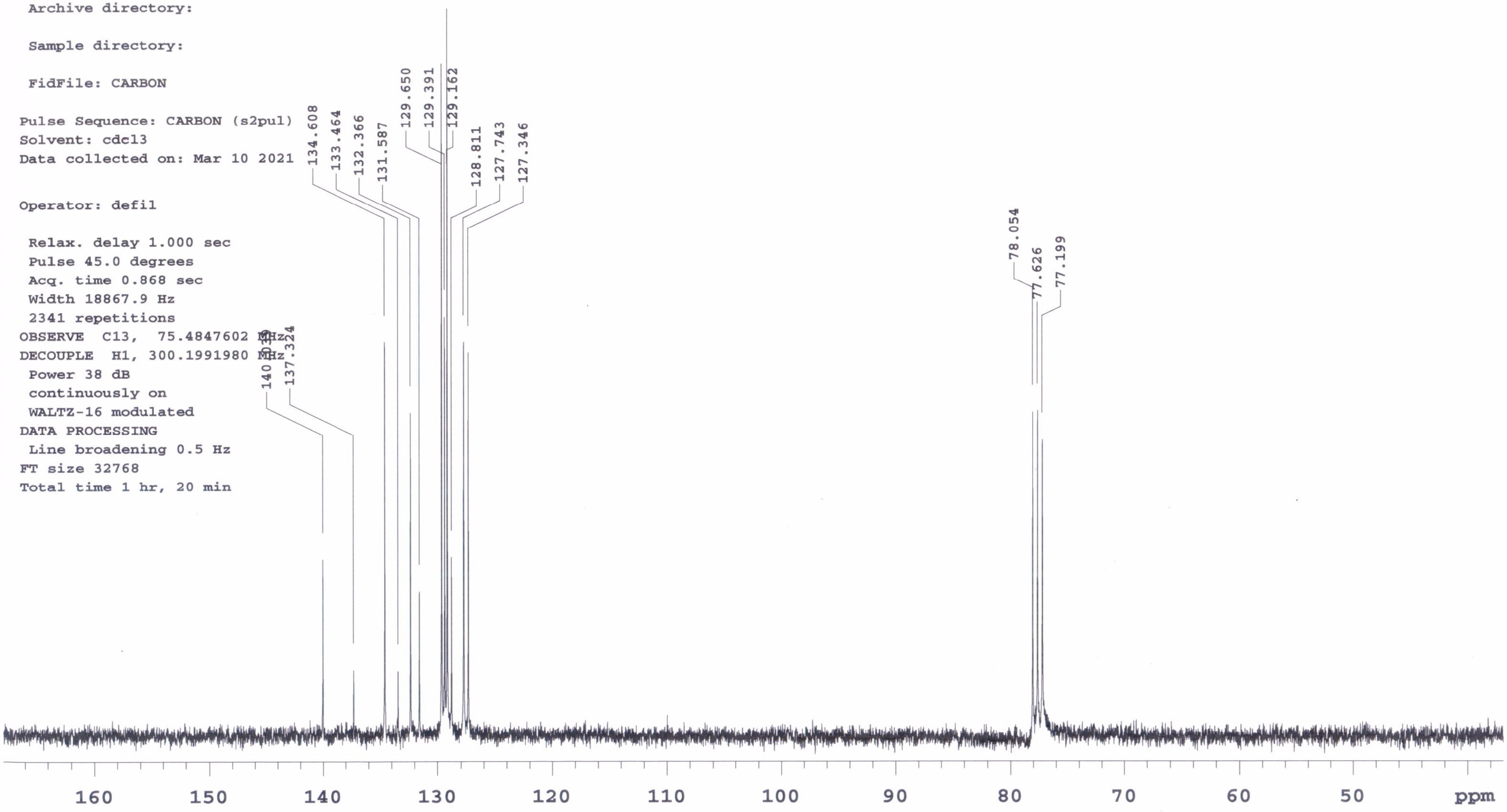

Figure S13*:* ^{1}H NMR and ^{13}C spectrum (300 MHz, C*D*Cl$_3$) of

3-nitro-N-{4-[(E)-2-phenylvinyl]phenyl}benzenesulfonamide ***2b***

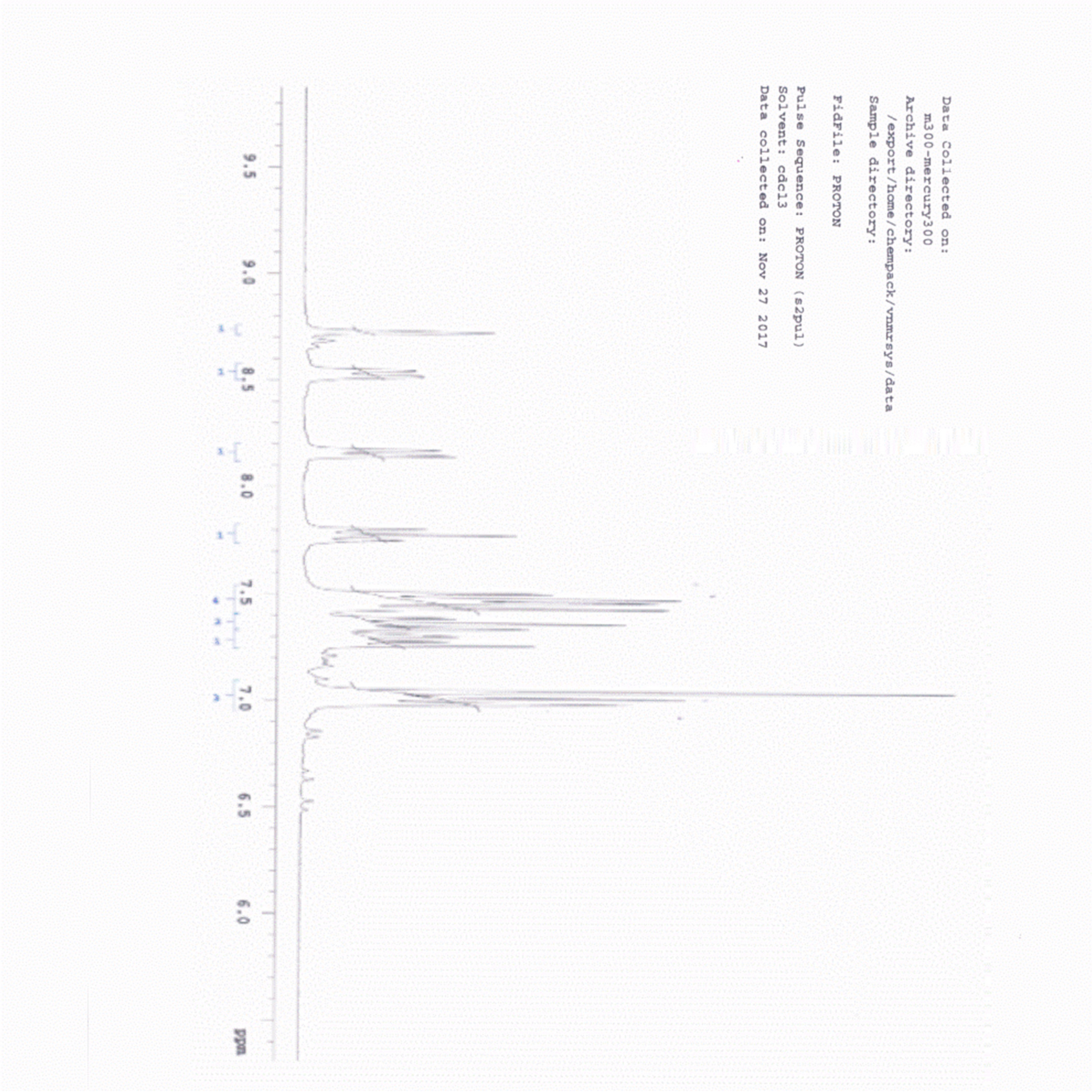

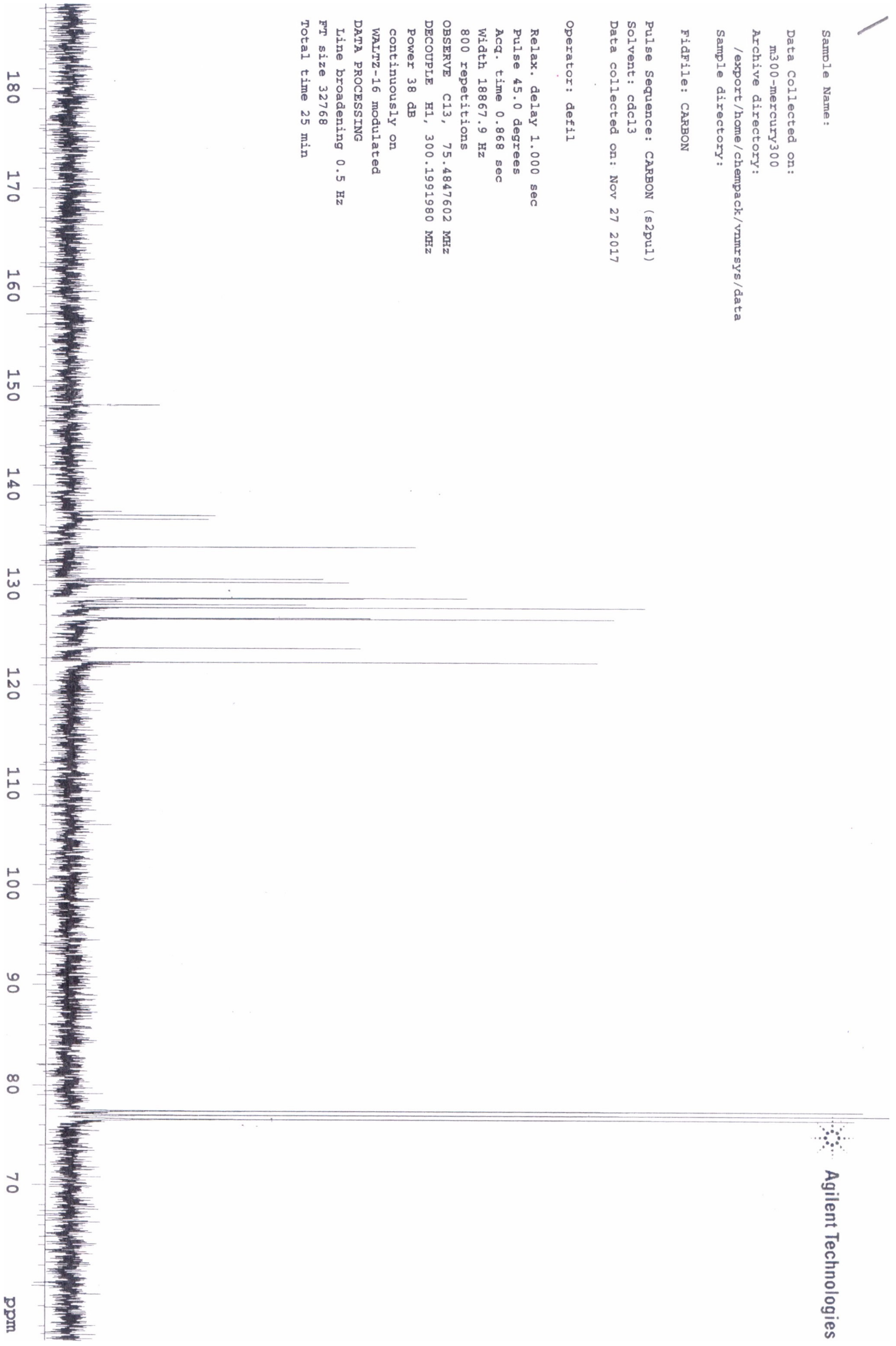
Sample Name:
Data Collected on:
m300-mercury300
Archive directory:
/export/home/chempack/vnmrsys/data
Sample directory:
FidFile: CARBON
Pulse Sequence: CARBON (s2pul)
Solvent: cdcl3
Data collected on: Nov 27 2017
Operator: defil
Relax. delay 1.000 sec
Pulse 45.0 degrees
Acq. time 0.868 sec
Width 18867.9 Hz
800 repetitions
OBSERVE C13, 75.4847602 MHz
DECOUPLE H1, 300.1991980 MHz
Power 38 dB
continuously on
WALTZ-16 modulated
DATA PROCESSING
Line broadening 0.5 Hz
FT size 32768
Total time 25 min
Agilent Technologies
180
170
160
150
140
130
120
110
100
90
80
70
ppm

Figure S14: ^{1}H NMR(300 MHz, C*D*Cl$_3$) and ^{13}C spectra (300 MHz, C*D*Cl$_3$) of

4-methyl-3-nitro-N-{4-[(E)-2-phenylvinyl]phenyl}benzenesulfonamide ***2c***

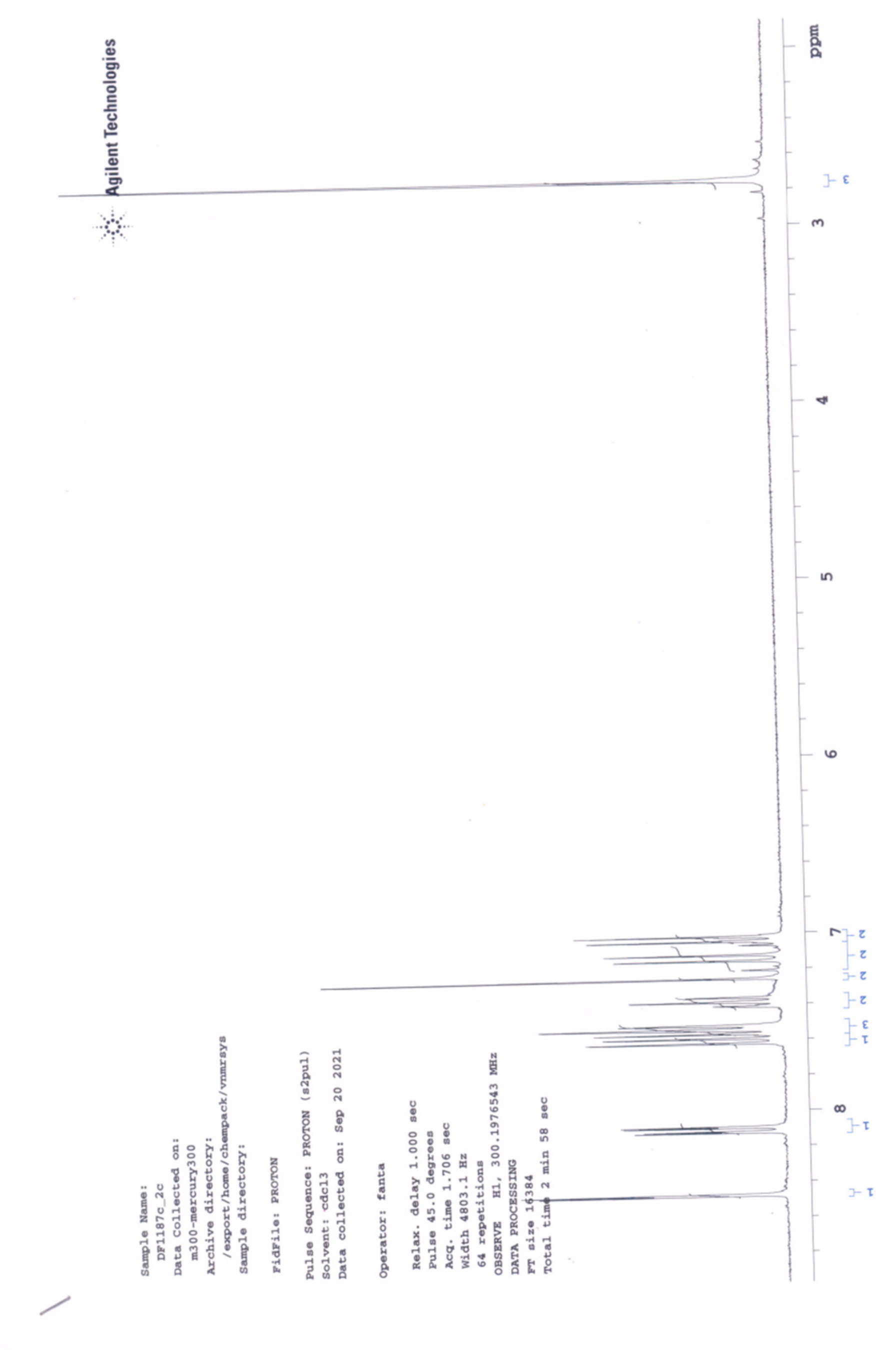

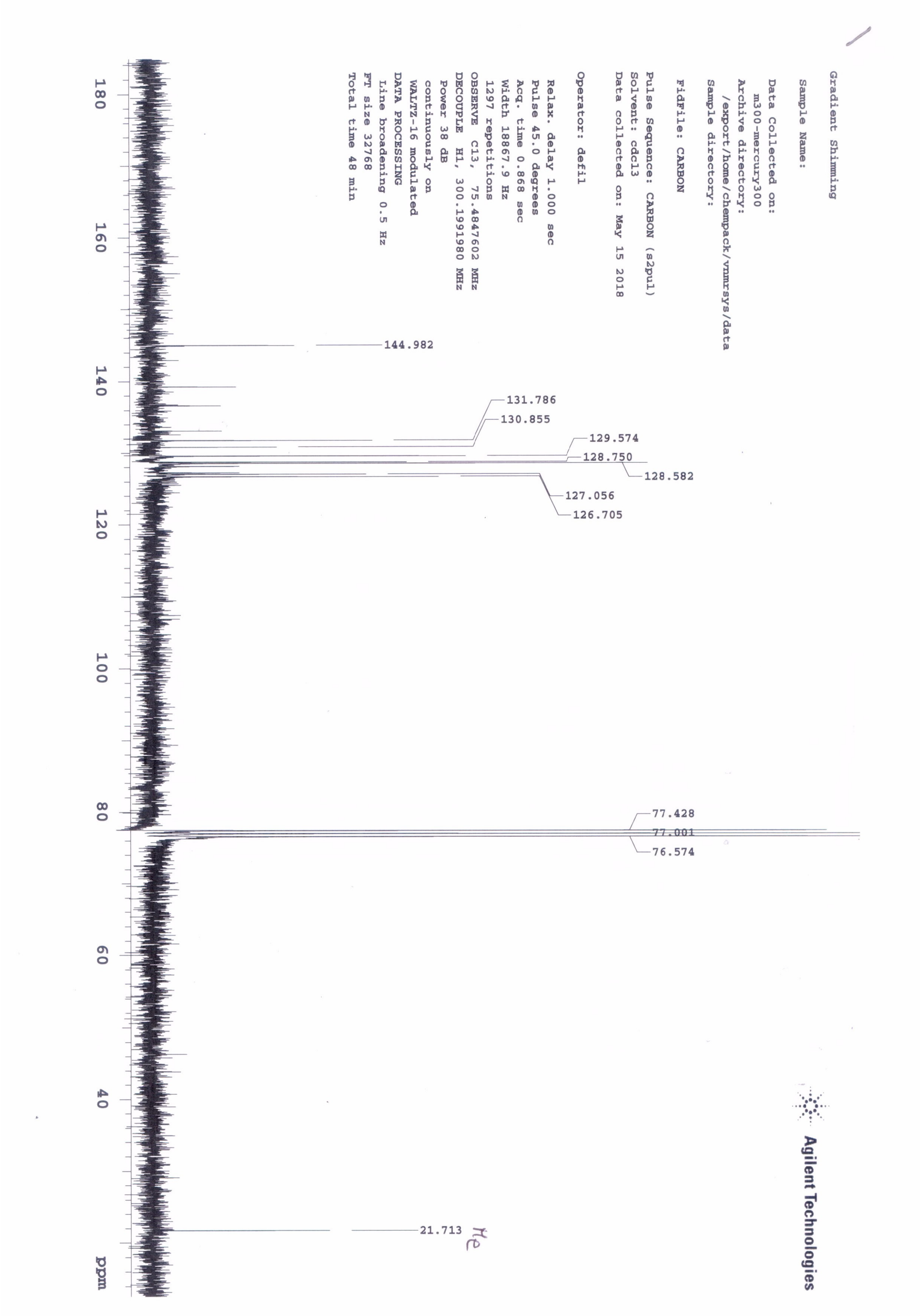
Gradient Shimming
Sample Name:
Data Collected on:
m300-mercury300
Archive directory:
/export/home/chempack/vnmrsys/data
Sample directory:
FidFile: CARBON
Pulse Sequence: CARBON (s2pul)
Solvent: cdcl3
Data collected on: May 15 2018
Operator: defi1
Relax. delay 1.000 sec
Pulse 45.0 degrees
Acq. time 0.868 sec
Width 18867.9 Hz
1297 repetitions
OBSERVE C13, 75.4847602 MHz
DECOUPLE H1, 300.1991980 MHz
Power 38 dB
continuously on
WALTZ-16 modulated
DATA PROCESSING
Line broadening 0.5 Hz
FT size 32768
Total time 48 min
144.982
131.786
130.855
129.574
128.750
128.582
127.056
126.705
77.428
77.001
76.574
21.713
Me
180
160
140
120
100
80
60
40
ppm
Agilent Technologies

Figure S15*:* ^{1}H NMR and ^{13}C spectrum (300 MHz, C*D*Cl$_3$) of *4-methyl-N-{4-[(E)-2-phenylvinyl]phenyl}benzenesulfonamide* ***2d***

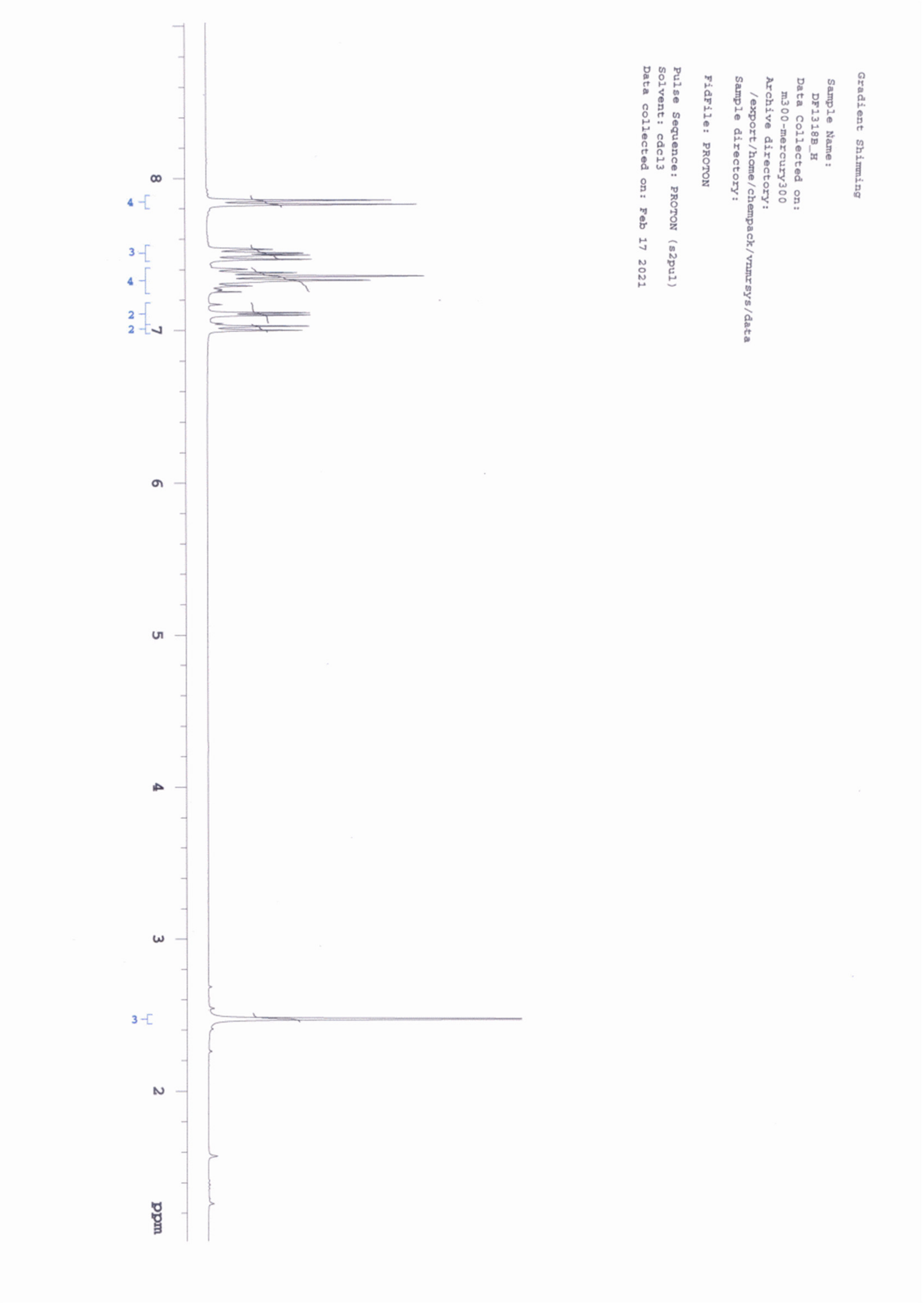

Sample Name:
DF1318B_H
Data Collected on:
m300-mercury300
Archive directory:

Sample directory:

FidFile: CARBON

Pulse Sequence: CARBON (s2pul)
Solvent: cdcl3
Data collected on: Feb 18 2021

Operator: defil

Relax. delay 1.000 sec
Pulse 45.0 degrees
Acq. time 0.868 sec
Width 18867.9 Hz
1500 repetitions
OBSERVE C13, 75.4847602 MHz
DECOUPLE H1, 300.1991980 MHz
Power 38 dB
continuously on
WALTZ-16 modulated
DATA PROCESSING
Line broadening 0.5 Hz
FT size 32768
Total time 48 min

Agilent Technologies

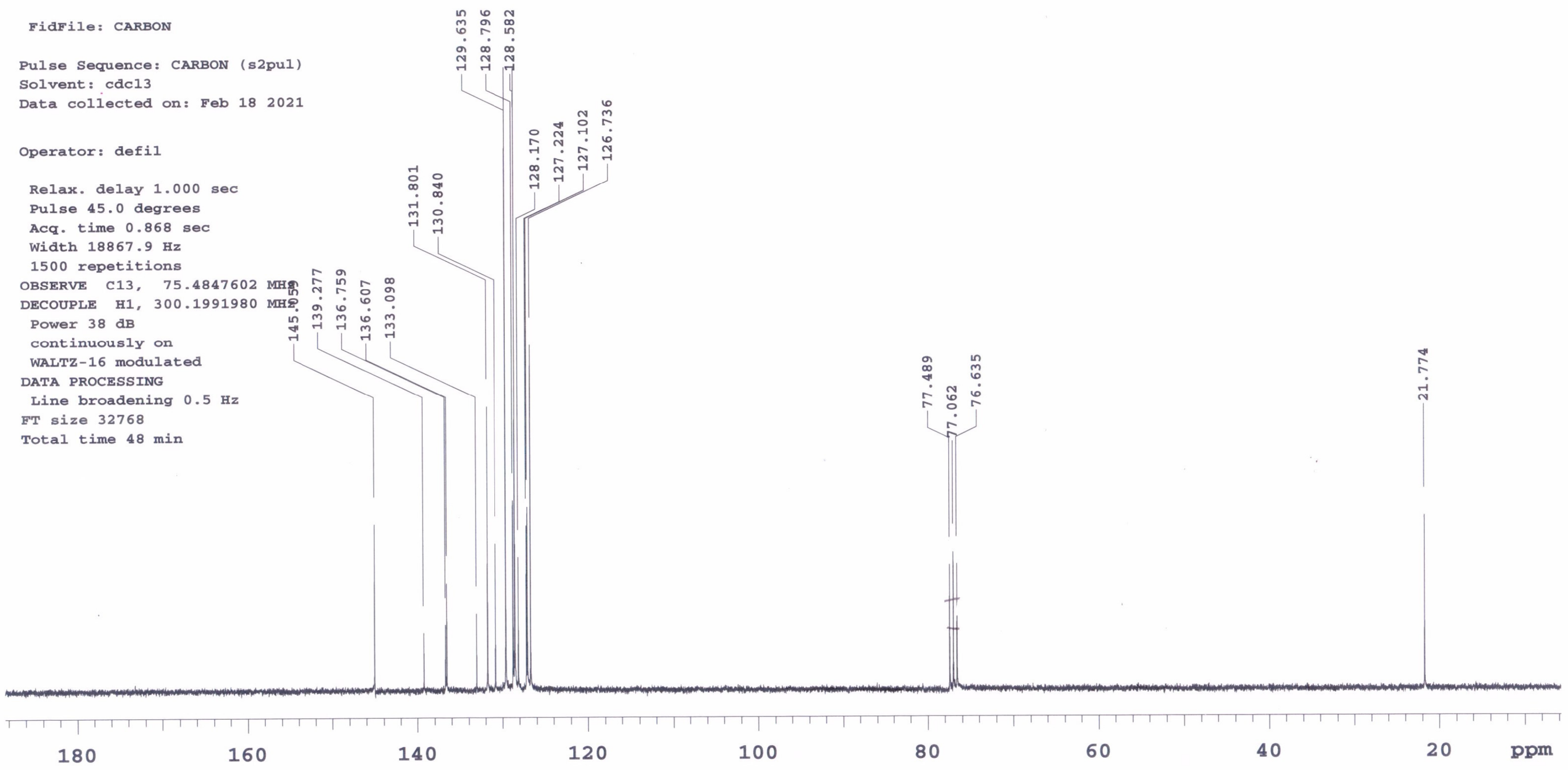

4-cyano-N-{4-[(E)-2-phenylvinyl]phenyl}benzenesulfonamide ***2e***

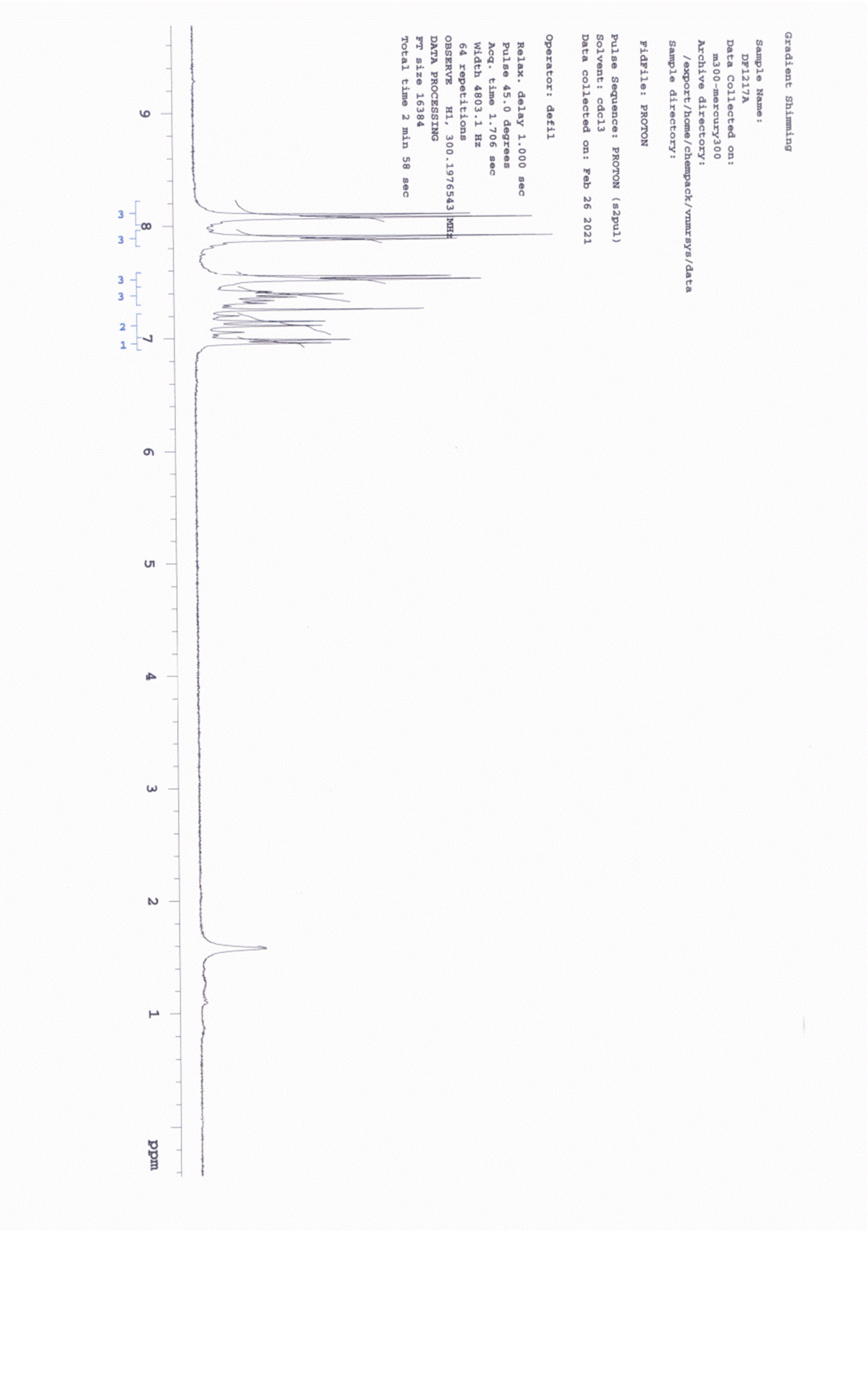

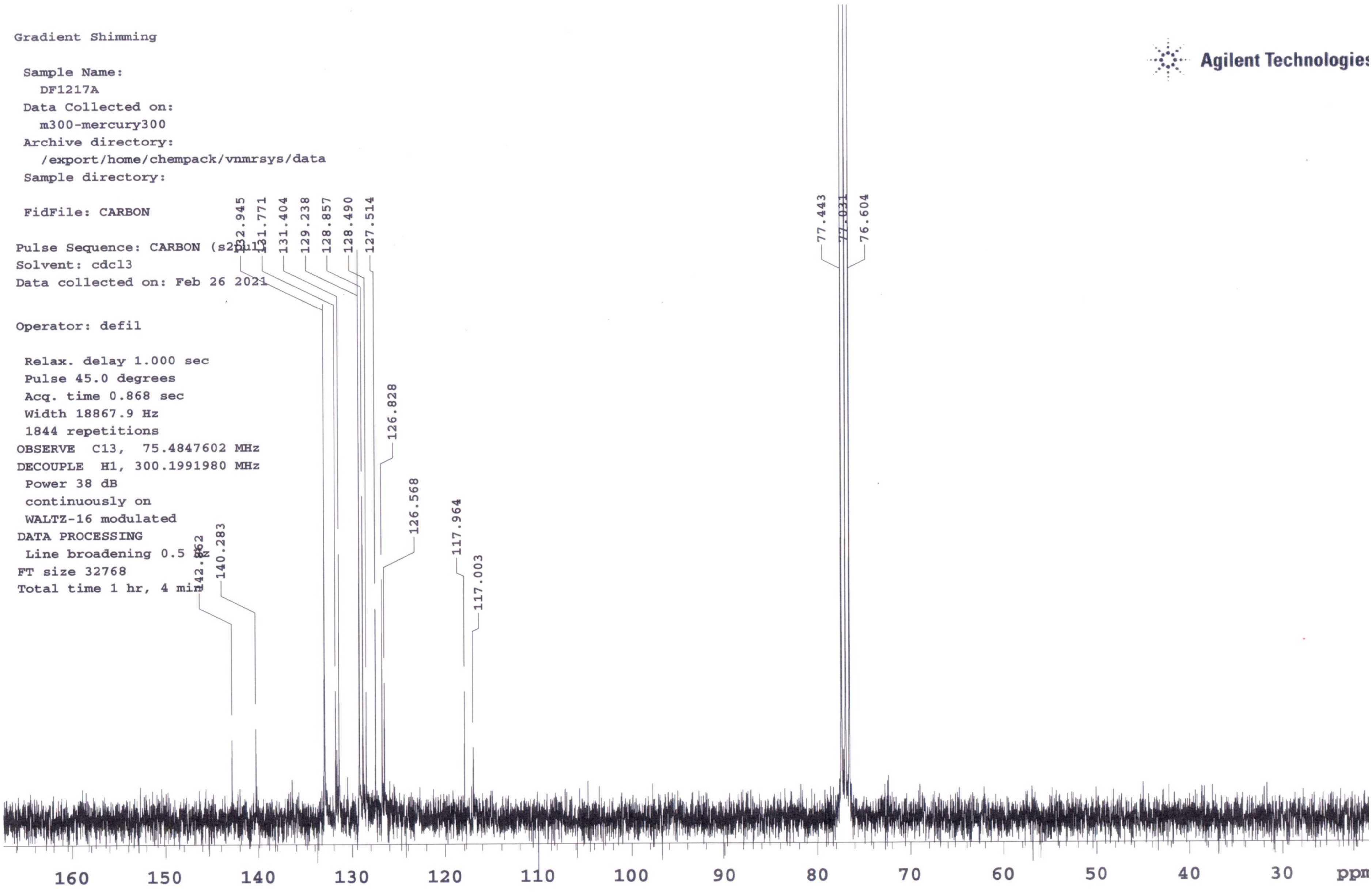
Gradient Shimming
Sample Name:
DF1217A
Data Collected on:
m300-mercury300
Archive directory:
/export/home/chempack/vnmrsys/data
Sample directory:
FidFile: CARBON
Pulse Sequence: CARBON (s2pul)
Solvent: cdcl3
Data collected on: Feb 26 2021
Operator: defil
Relax. delay 1.000 sec
Pulse 45.0 degrees
Acq. time 0.868 sec
Width 18867.9 Hz
1844 repetitions
OBSERVE C13, 75.4847602 MHz
DECOUPLE H1, 300.1991980 MHz
Power 38 dB
continuously on
WALTZ-16 modulated
DATA PROCESSING
Line broadening 0.5 [illegible]
FT size 32768
Total time 1 hr, 4 min
Agilent Technologies
142.[illegible]62
140.283
132.945
131.771
131.404
129.238
128.857
128.490
127.514
126.828
126.568
117.964
117.003
77.443
77.031
76.604
160
150
140
130
120
110
100
90
80
70
60
50
40
30
ppm

Figure S17: ^{1}H NMR (300 MHz, Acetone-*d*) and ^{13}C spectrum (300 MHz, $CDCl_3$) of *N-{4-[({4-[(E)-2-phenylvinyl]phenyl}amino)sulfonyl]phenyl}acetamide* ***2f***

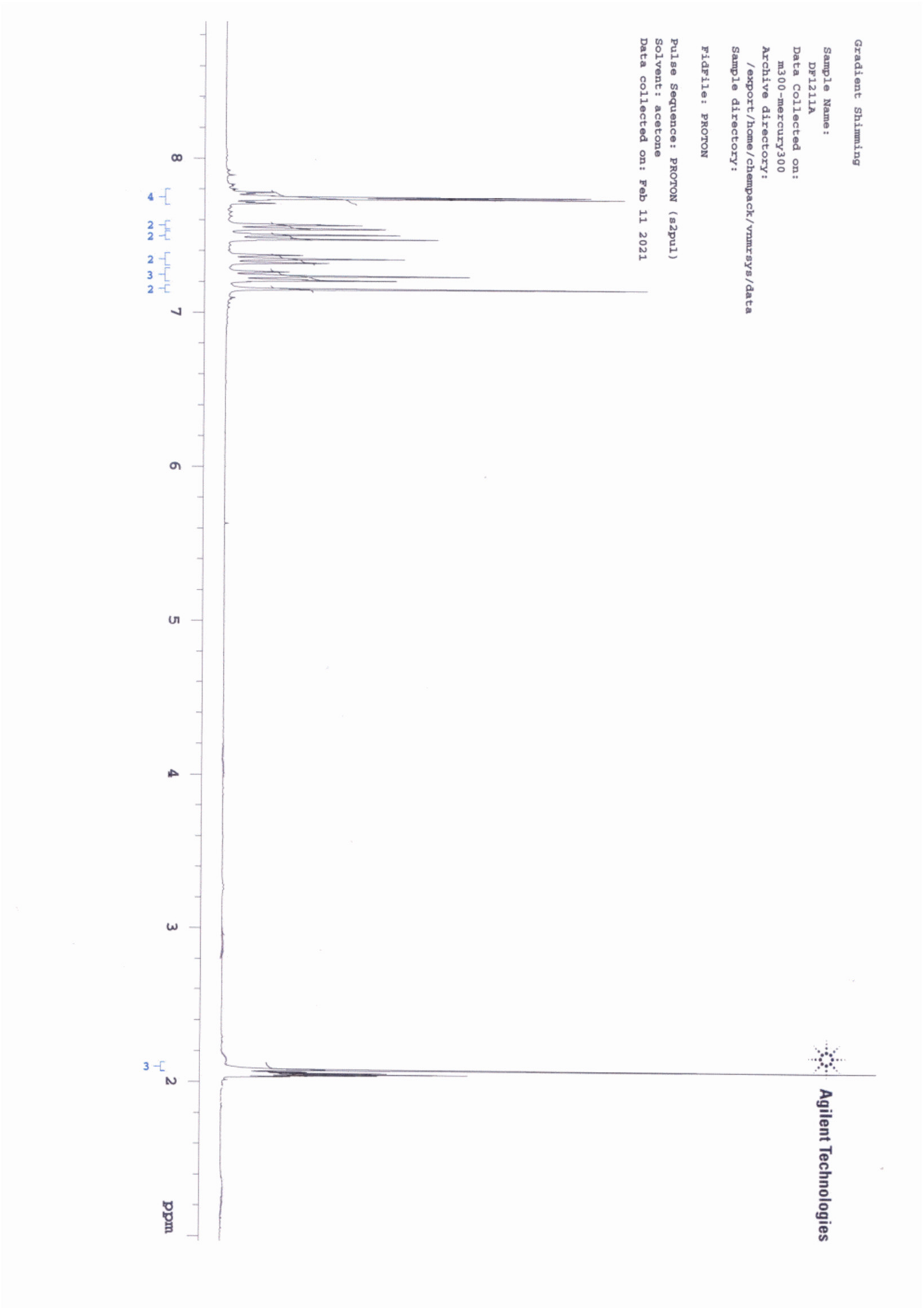

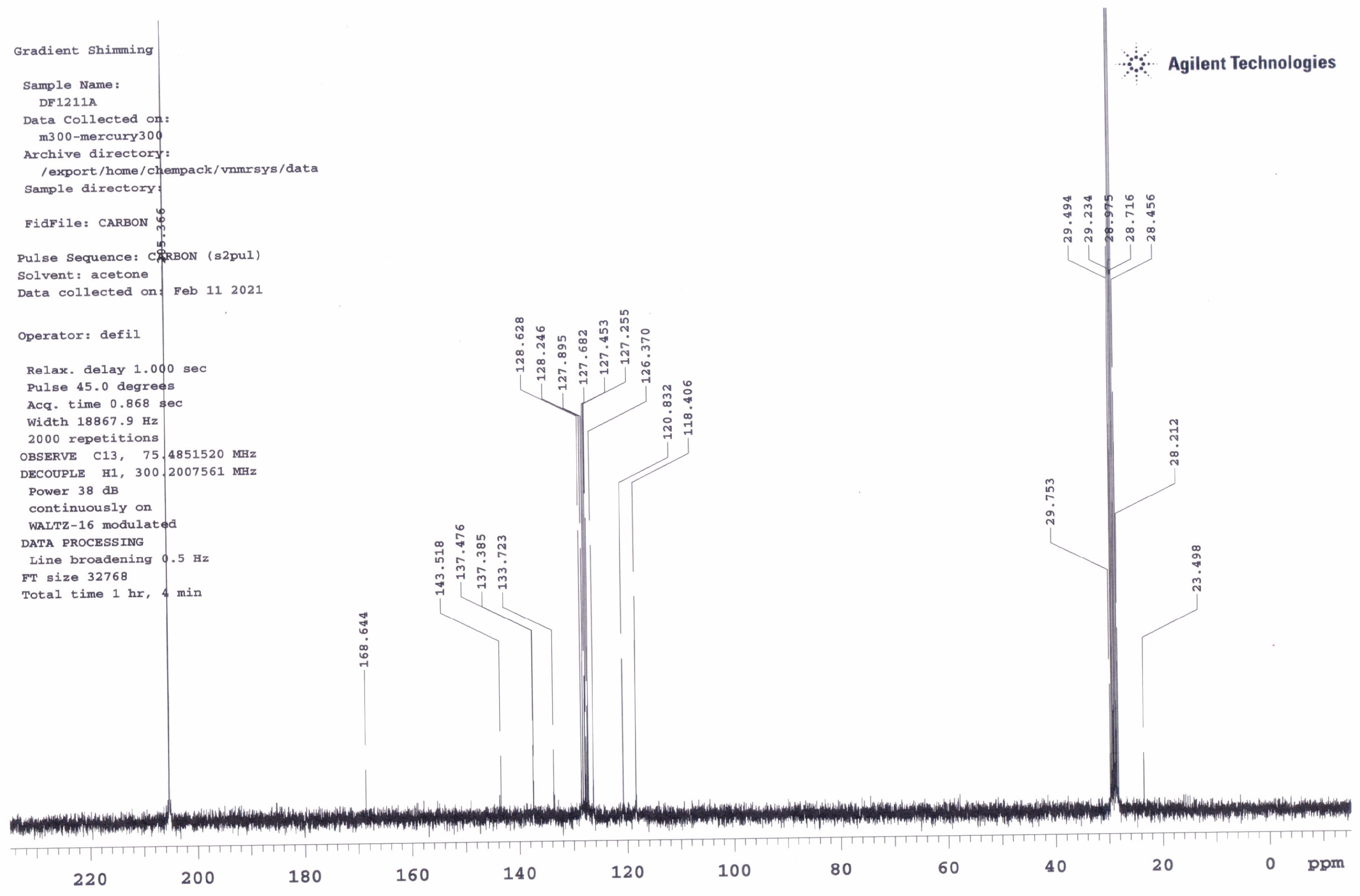
Gradient Shimming
Sample Name:
DF1211A
Data Collected on:
m300-mercury300
Archive directory:
/export/home/chempack/vnmrsys/data
Sample directory:
FidFile: CARBON
Pulse Sequence: CARBON (s2pul)
Solvent: acetone
Data collected on: Feb 11 2021
Operator: defil
Relax. delay 1.000 sec
Pulse 45.0 degrees
Acq. time 0.868 sec
Width 18867.9 Hz
2000 repetitions
OBSERVE C13, 75.4851520 MHz
DECOUPLE H1, 300.2007561 MHz
Power 38 dB
continuously on
WALTZ-16 modulated
DATA PROCESSING
Line broadening 0.5 Hz
FT size 32768
Total time 1 hr, 4 min
Agilent Technologies
205.366
168.644
143.518
137.476
137.385
133.723
128.628
128.246
127.895
127.682
127.453
127.255
126.370
120.832
118.406
29.753
29.494
29.234
28.975
28.716
28.456
28.212
23.498
220
200
180
160
140
120
100
80
60
40
20
0
ppm

Figure S18: ^{1}H NMR and ^{13}C spectrum (300 MHz, $CDCl_3$) of *2,4-dimethoxy-N-{4-[(E)-2-phenylvinyl]phenyl}benzenesulfonamide* ***2g***

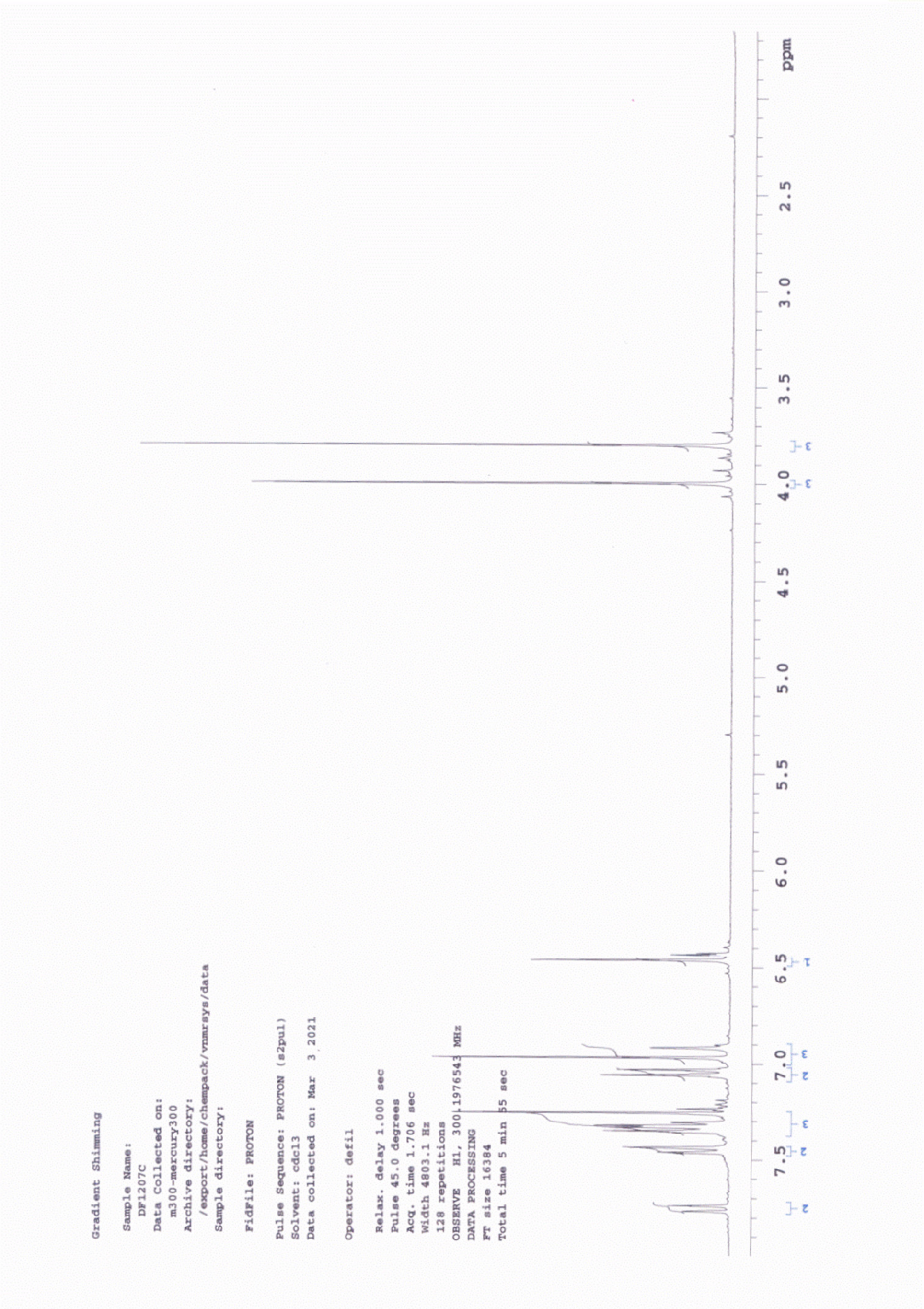

Gradient Shimming

Sample Name:
DF1207C
Data Collected on:
m300-mercury300
Archive directory:
/export/home/chempack/vnmrsys/data
Sample directory:

FidFile: CARBON

Pulse Sequence: CARBON (s2pul)
Solvent: cdcl3
Data collected on: Mar 3 2021

Operator: defil

Relax. delay 1.000 sec
Pulse 45.0 degrees
Acq. time 0.868 sec
Width 18867.9 Hz
2000 repetitions
OBSERVE C13, 75.4847602 MHz
DECOUPLE H1, 300.1991980 MHz
Power 38 dB
continuously on
WALTZ-16 modulated
DATA PROCESSING
Line broadening 0.5 Hz
FT size 32768
Total time 1 hr, 4 min

Agilent Technologies

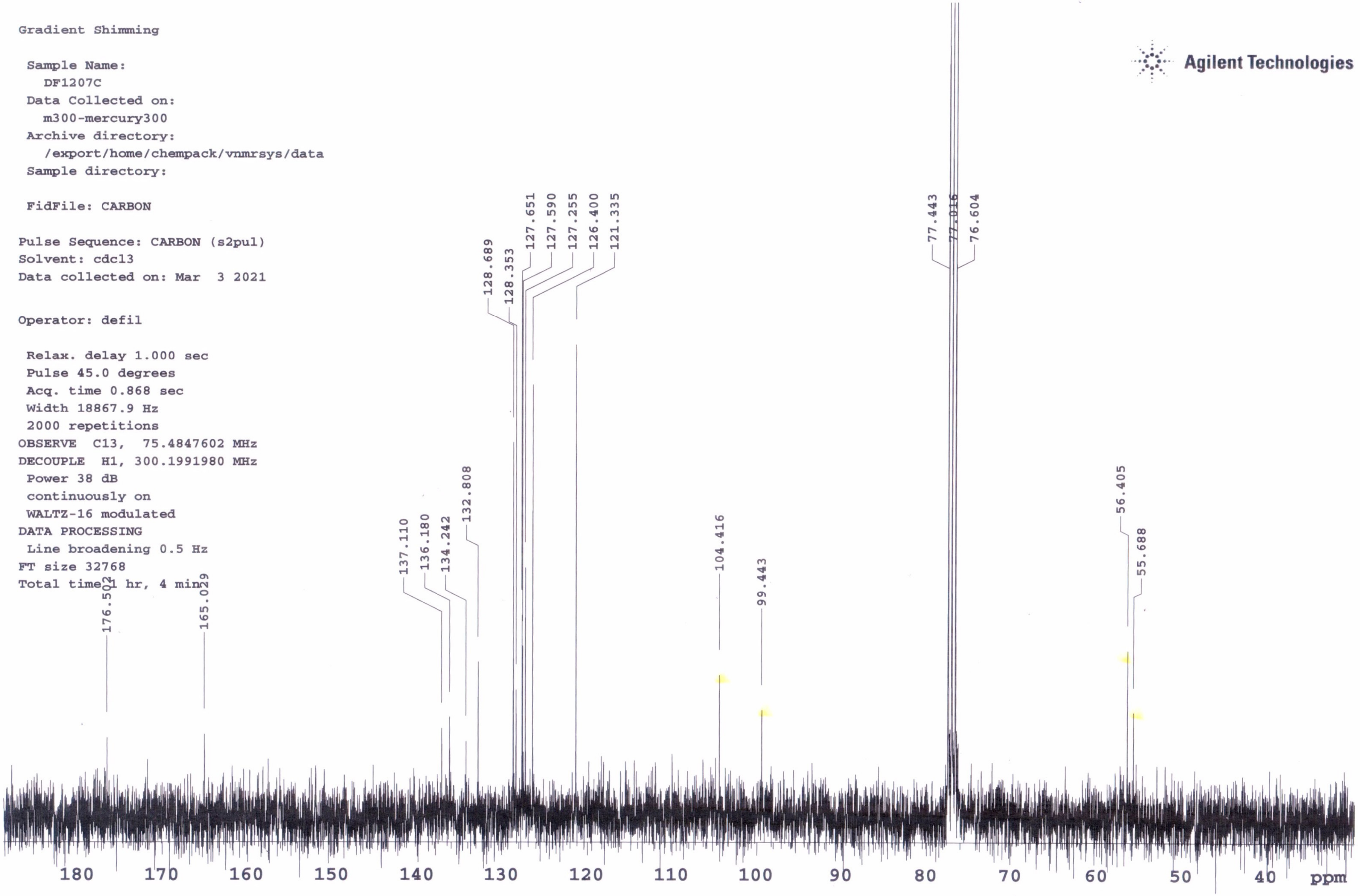

Figure S19: ^{1}H NMR and ^{13}C spectrum (300 MHz, C*D*Cl$_3$) of *N-{4-[(E)-2-phenylvinyl]phenyl}methanesulfonamide* ***2h***

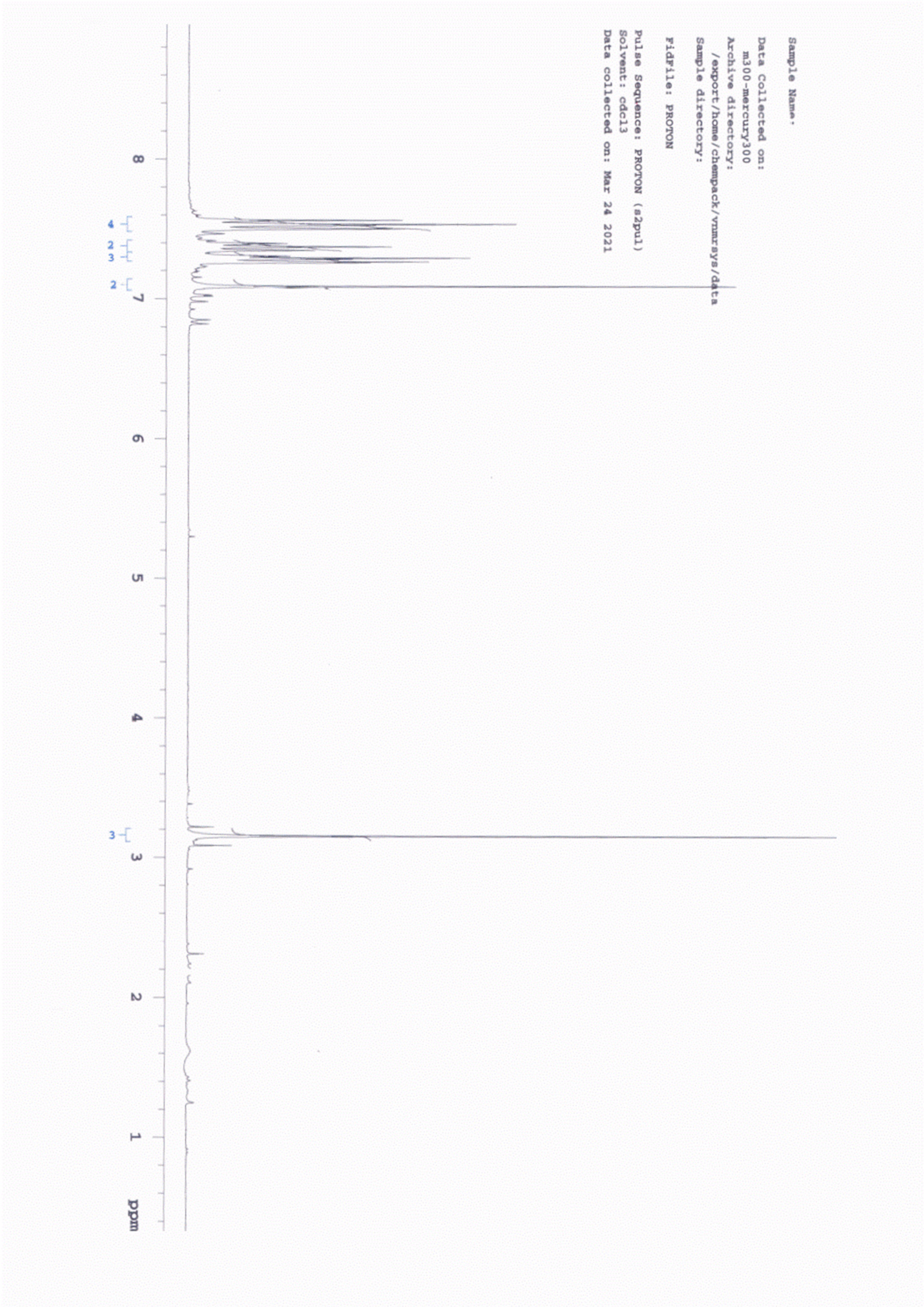

Agilent Technologies

```
Sample Name:
  Df1213A
Data Collected on:
  m300-mercury300
Archive directory:
  /export/home/chempack/vnmrsys/data
Sample directory:

FidFile: CARBON

Pulse Sequence: CARBON (s2pul)
Solvent: cdcl3
Data collected on: Jul  2 2021

Operator: fanta

Relax. delay 1.000 sec
Pulse 45.0 degrees
Acq. time 0.868 sec
Width 18867.9 Hz
2000 repetitions
OBSERVE  C13,  75.4847602 MHz
DECOUPLE  H1, 300.1991980 MHz
Power 38 dB
continuously on
WALTZ-16 modulated
DATA PROCESSING
Line broadening 0.5 Hz
FT size 32768
Total time 1 hr, 4 min
```

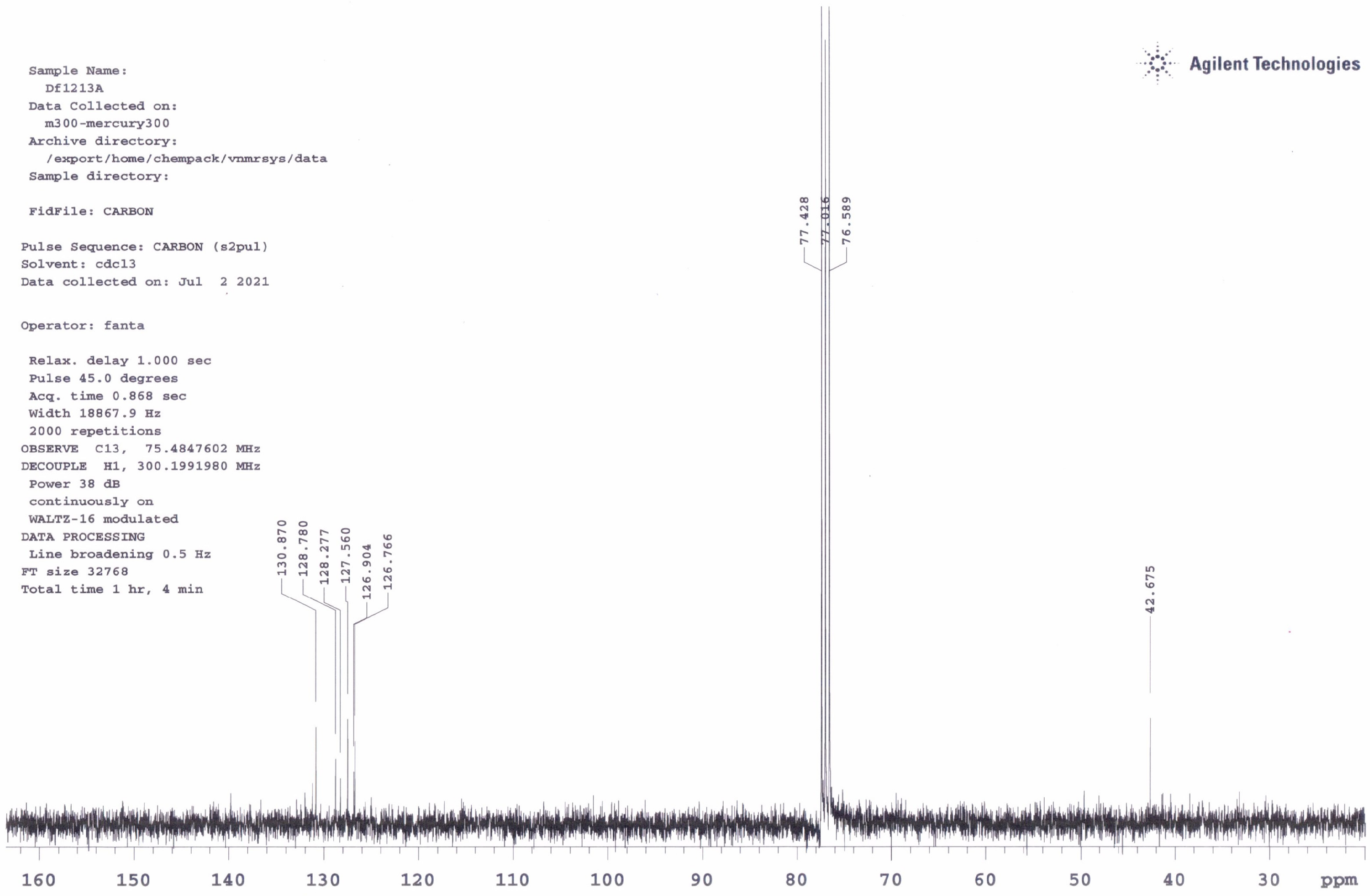

Figure S20: ^{1}H NMR and ^{13}C spectrum (300 MHz, $CDCl_3$) of *N-{4-[(E)-2-phenylvinyl]phenyl}ethanesulfonamide* ***2i***

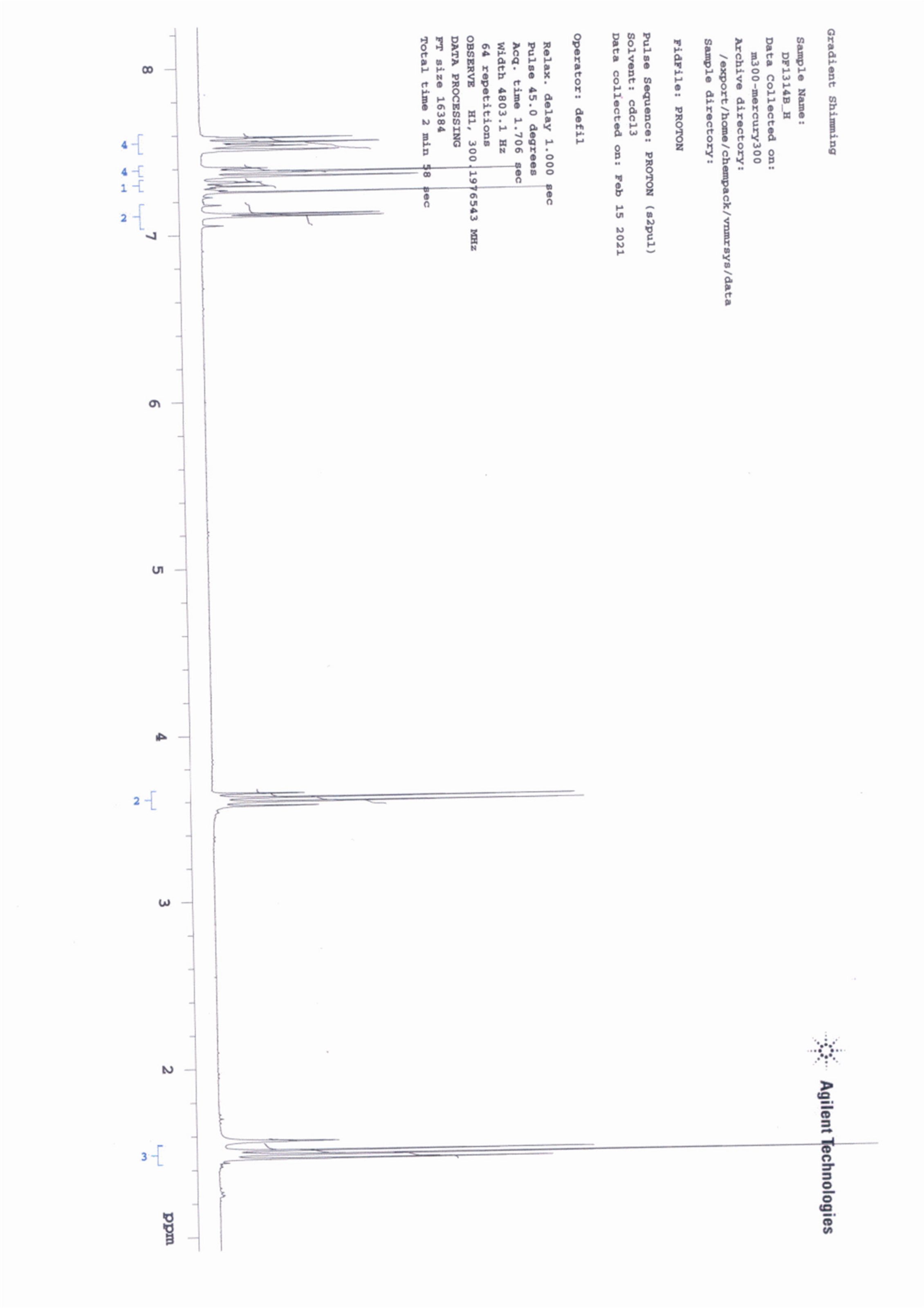

Gradient Shimming

Sample Name:
DF1314B_C
Data Collected on:
m300-mercury300
Archive directory:
/export/home/chempack/vnmrsys/data
Sample directory:

FidFile: CARBON

Pulse Sequence: CARBON (s2pul)
Solvent: cdcl3
Data collected on: Feb 15 2021

Operator: defil

Relax. delay 1.000 sec
Pulse 45.0 degrees
Acq. time 0.868 sec
Width 18867.9 Hz
2048 repetitions
OBSERVE C13, 75.4847602 MHz
DECOUPLE H1, 300.1991980 MHz
Power 38 dB
continuously on
WALTZ-16 modulated
DATA PROCESSING
Line broadening 0.5 Hz
FT size 32768
Total time 1 hr, 14 min

Agilent Technologies

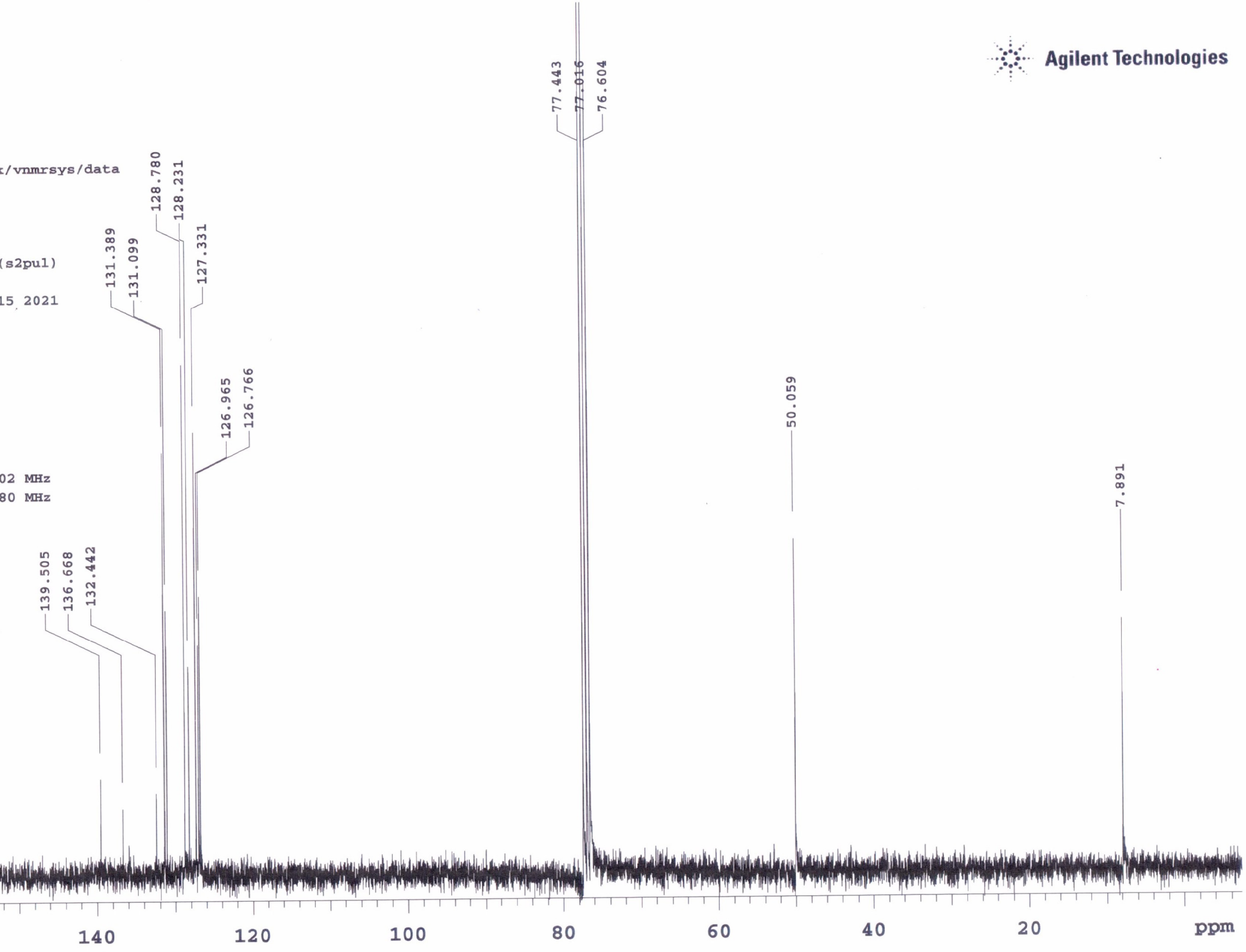

Figure S21: ^{1}H NMR and ^{13}C spectrum (300 MHz, $CDCl_3$) of *1-phenyl-N-{4-[(E)-2-phenylvinyl]phenyl}methanesulfonamide-**2j***

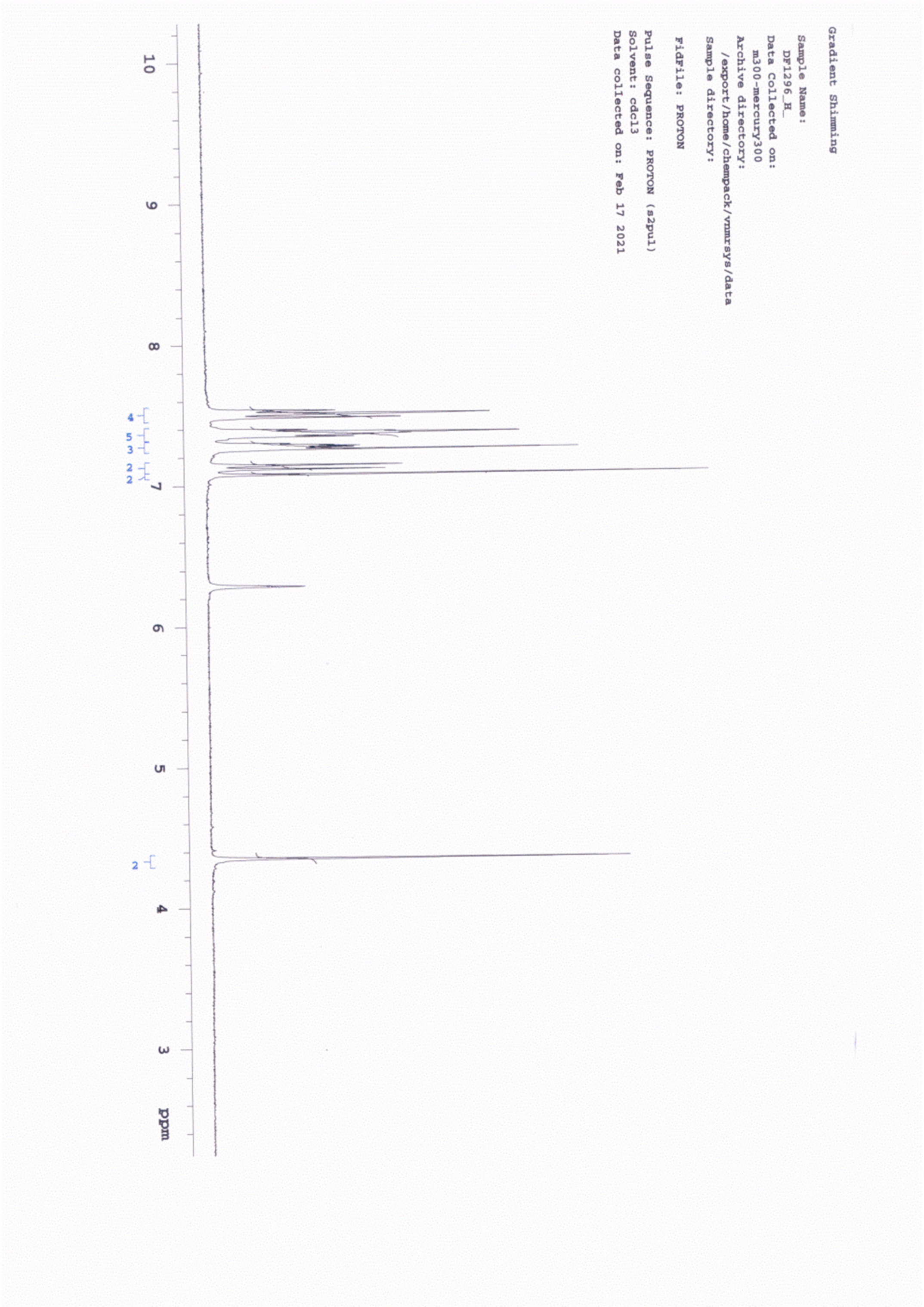

Gradient Shimming

Sample Name:
DF1296_H
Data Collected on:
m300-mercury300
Archive directory:
/export/home/chempack/vnmrsys/data
Sample directory:

FidFile: CARBON

Pulse Sequence: CARBON (s2pul)
Solvent: cdcl3
Data collected on: Feb 17 2021

Operator: defil

Relax. delay 1.000 sec
Pulse 45.0 degrees
Acq. time 0.868 sec
Width 18867.9 Hz
1894 repetitions
OBSERVE C13, 75.4847602 MHz
DECOUPLE H1, 300.1991980 MHz
Power 38 dB
continuously on
WALTZ-16 modulated
DATA PROCESSING
Line broadening 0.5 Hz
FT size 32768
Total time 1 hr, 20 min

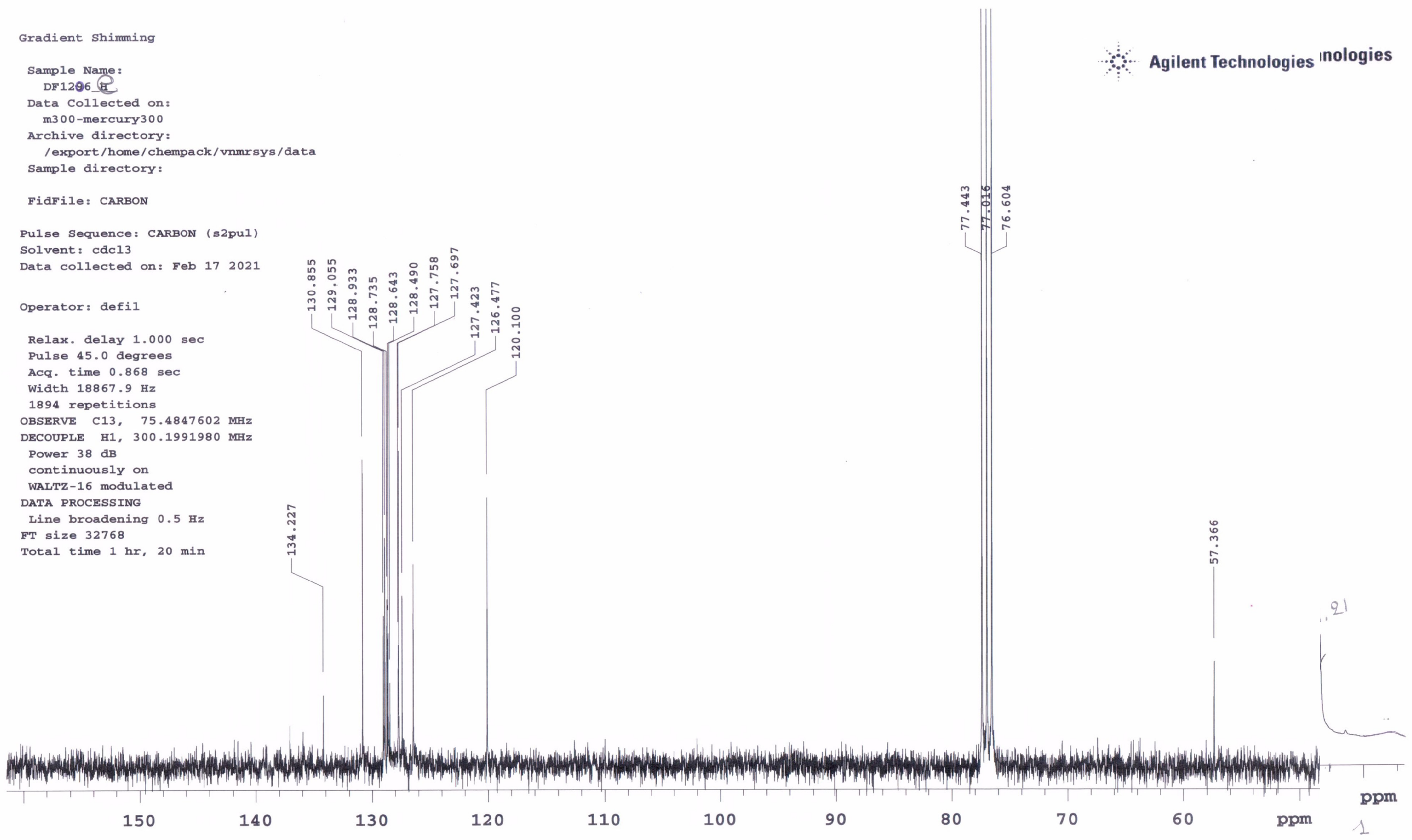

Agilent Technologies

phenylvinyl]phenyl}thiophene-2-sulfonamide ***2k***

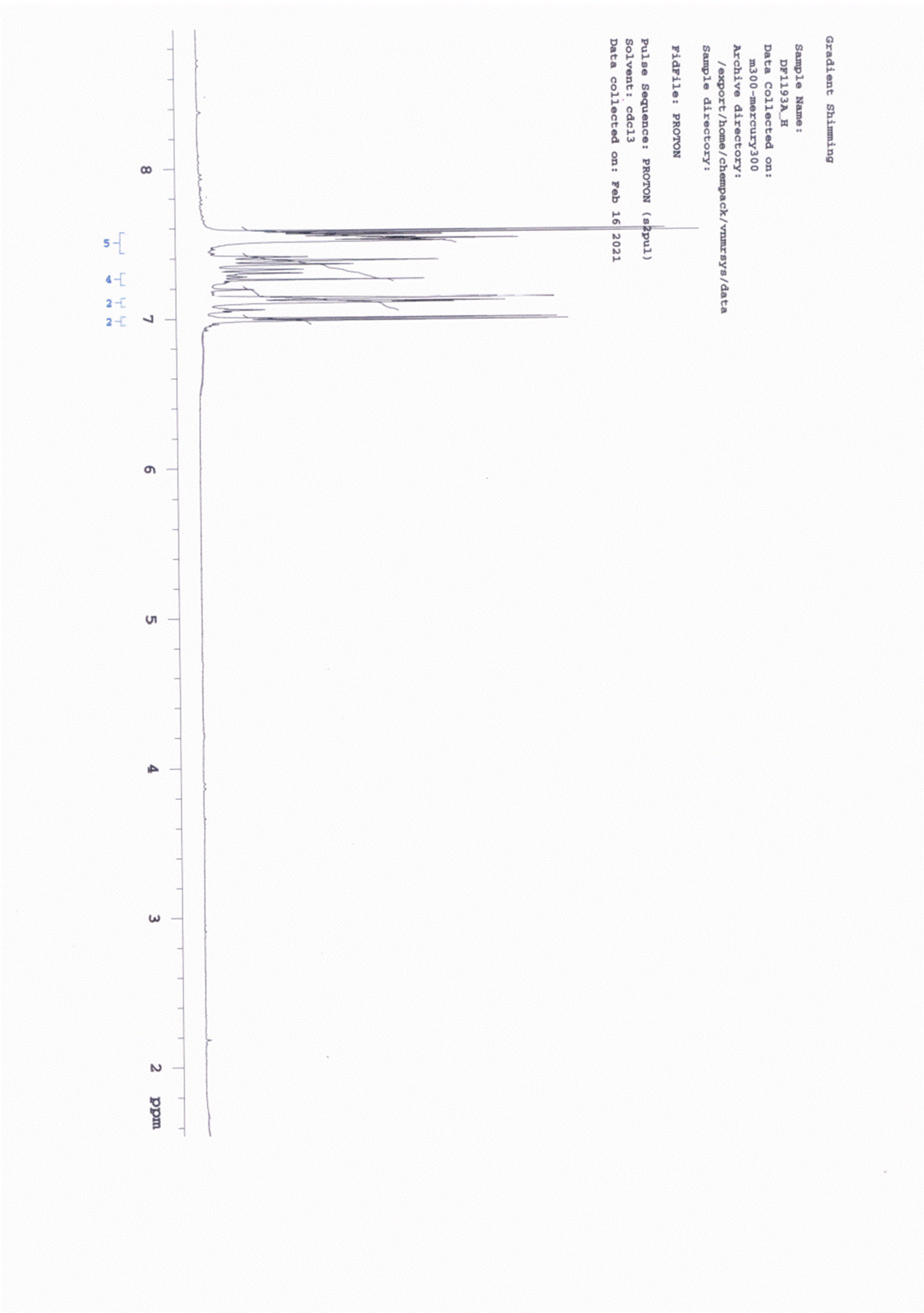

Gradient Shimming

Sample Name:
DF1193A_H
Data Collected on:
m300-mercury300
Archive directory:
/export/home/chempack/vnmrsys/data
Sample directory:

FidFile: CARBON

Pulse Sequence: CARBON (s2pul)
Solvent: cdcl3
Data collected on: Feb 16 2021

Operator: defil

Relax. delay 1.000 sec
Pulse 45.0 degrees
Acq. time 0.868 sec
Width 18867.9 Hz
1408 repetitions
OBSERVE C13, 75.4[illegible]47602 MHz
DECOUPLE H1, 300.1991980 MHz
Power 38 dB
continuously on
WALTZ-16 modulated
DATA PROCESSING
Line broadening 0.5 Hz
FT size 32768
Total time 58 min

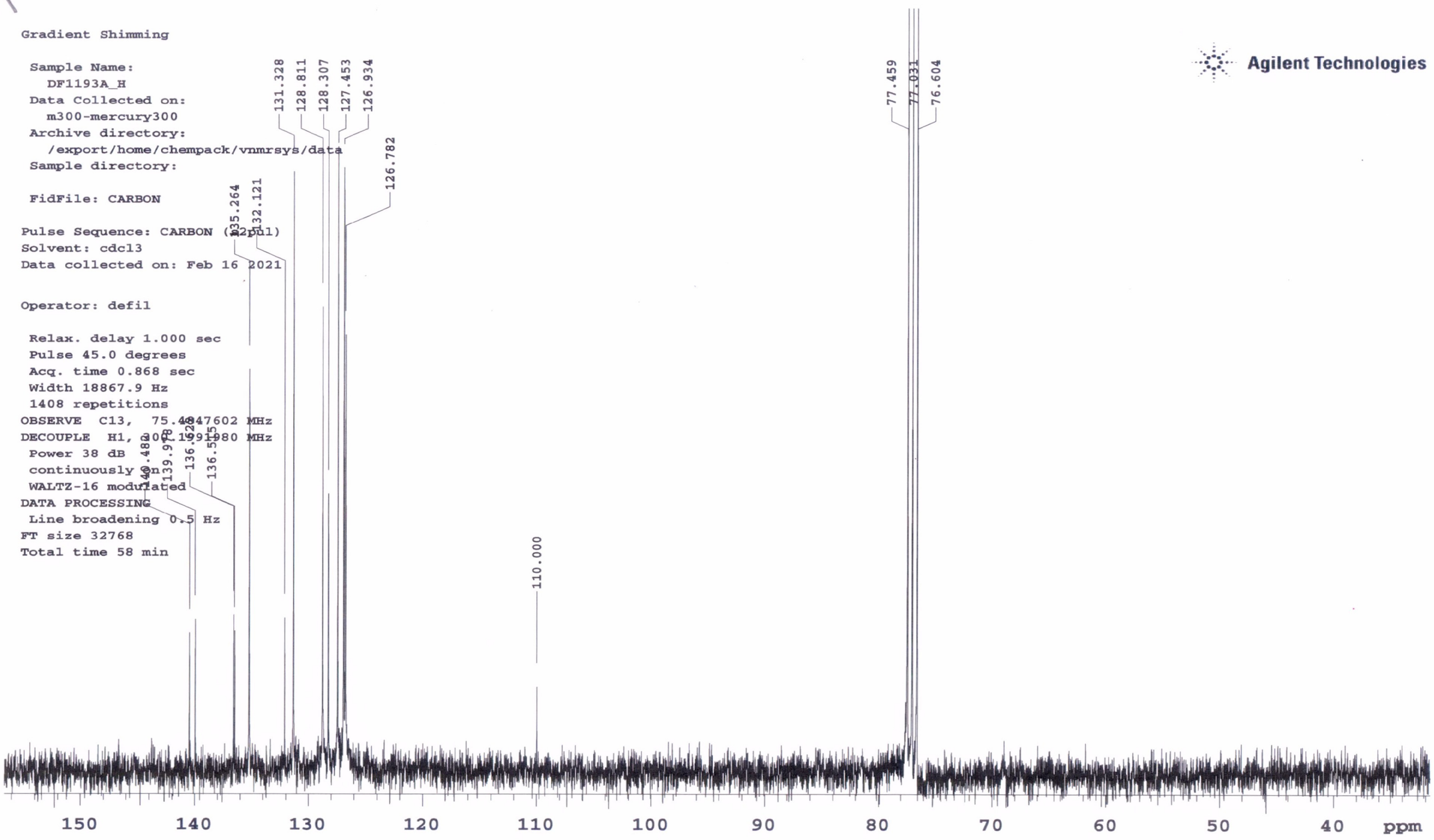

Agilent Technologies

Table S1. Raw data for the IC_{50} calculation related to MCF7 cells in the presence of increasing concentrations of compounds **1b**, **1c**, **1j** and Resveratrol for 24 and 72 h.

Compound 1b (μM)	Viability (% of control) - 24 h	Viability (% of control) - 72 h
10	69.32 ± 14.99	98.33 ± 5.24
25	79.12 ± 9.66	99.99 ± 9.30
50	80.69 ± 4.08	93.39 ± 5.39
100	73.93 ± 1.87	77.31 ± 7.02
250	45.57 ± 3.42	57.01 ± 1.01
500	35.83 ± 2.22	36.11 ± 0.35

Compound 1c (μM)	Viability (% of control) - 24 h	Viability (% of control) - 72 h
10	85.23 ± 1.29	96.62 ± 6.99
25	73.08 ± 2.67	91.88 ± 5.31
50	37.39 ± 0.66	46.19 ± 1.67
100	36.17 ± 1.56	35.22 ± 2.21
250	38.64 ± 1.23	38.30 ± 4.07
500	34.30 ± 1.43	15.08 ± 0.67

Compound 1j (μM)	Viability (% of control) - 24 h	Viability (% of control) - 72 h
10	85.47 ± 6.41	96.53 ± 7.12
25	86.33 ± 14.64	95.30 ± 7.88
50	70.06 ± 3.80	87.57 ± 5.26
100	74.91 ± 9.24	72.67 ± 3.46
250	51.84 ± 3.08	69.54 ± 4.11
500	38.88 ± 5.23	46.31 ± 4.73

Resveratrol (μM)	Viability (% of control) - 24 h	Viability (% of control) - 72 h
10	80.27 ± 3.18	91.92 ± 5.44
25	84.94 ± 6.59	73.93 ± 9.52
50	87.44 ± 1.93	66.54 ± 5.77
100	78.29 ± 4.03	47.75 ± 2.67
250	60.29 ± 0.93	38.05 ± 8.70
500	34.30 ± 5.01	15.33 ± 4.88

Figure S23: Chromatograms of the active compounds **1b-c** and **1j** in neutral conditions:

Capillary column: 100 µm x 15 cm packed with Chromspher C18 dp 3 µm; mobile phase, ACN/H_2O (75 /15 % (v/v) containing a 10 mM sodium hydrocarbonate buffer, pH 8.5; UV detection, 206 nm; the studied compounds at a final concentration of 100 µg/mL were dissolved with a solution of 10 mM phosphate buffer (pH 7.4); injection time 15 s; flow rate, 450 nL/min.

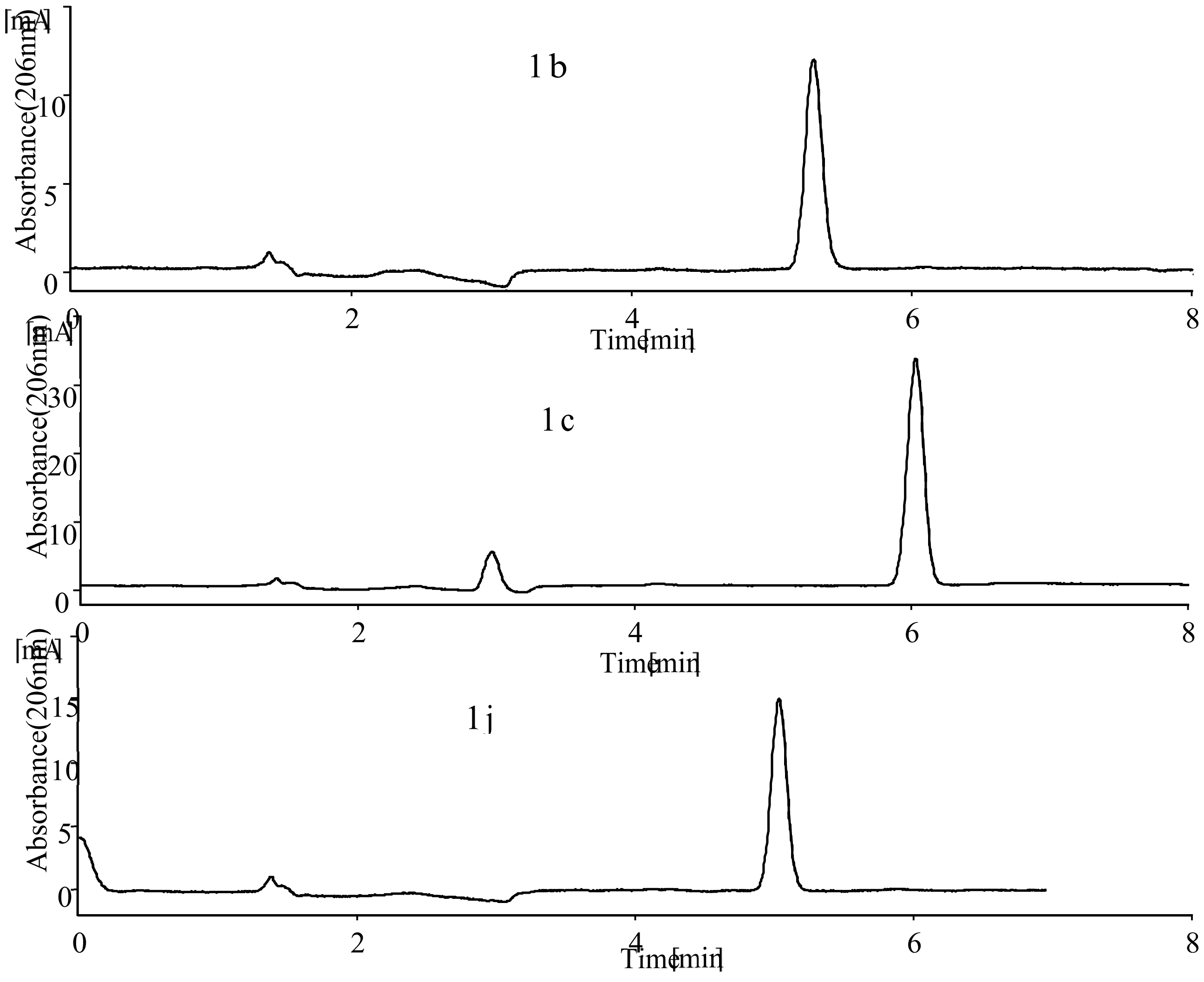

Supplement: Supplementary file 1 [file pharmaceuticals-14-00984-s001.zip › pharmaceuticals-1355781-supplementary.pdf]
